# Personalized Integrated Network Modeling of the Cancer Proteome Atlas

**DOI:** 10.1038/s41598-018-32682-x

**Published:** 2018-10-08

**Authors:** Min Jin Ha, Sayantan Banerjee, Rehan Akbani, Han Liang, Gordon B. Mills, Kim-Anh Do, Veerabhadran Baladandayuthapani

**Affiliations:** 1 Department of Biostatistics, The University of Texas MD Anderson Cancer Center, Houston, TX 77030 USA; 2 Operations Management and Quantitative, Techniques Area at the Indian Institute of Management, Indore, India; 3 Department of Bioinformatics and Computational Biology, The University of Texas MD Anderson Cancer Center, Houston, TX 77030 USA; 4 Department of Systems Biology, The University of Texas MD Anderson Cancer Center, Houston, TX 77030 USA; 5 Oregon Health and Science University, Portland, OR 97239 USA; 6 Department of Biostatistics, University of Michigan, Ann Arbor, MI 48109 USA

## Abstract

Personalized (patient-specific) approaches have recently emerged with a precision medicine paradigm that acknowledges the fact that molecular pathway structures and activity might be considerably different within and across tumors. The functional cancer genome and proteome provide rich sources of information to identify patient-specific variations in signaling pathways and activities within and across tumors; however, current analytic methods lack the ability to exploit the diverse and multi-layered architecture of these complex biological networks. We assessed pan-cancer pathway activities for >7700 patients across 32 tumor types from The Cancer Proteome Atlas by developing a personalized cancer-specific integrated network estimation (PRECISE) model. PRECISE is a general Bayesian framework for integrating existing interaction databases, data-driven *de novo* causal structures, and upstream molecular profiling data to estimate cancer-specific integrated networks, infer patient-specific networks and elicit interpretable pathway-level signatures. PRECISE-based pathway signatures, can delineate pan-cancer commonalities and differences in proteomic network biology within and across tumors, demonstrates robust tumor stratification that is both biologically and clinically informative and superior prognostic power compared to existing approaches. Towards establishing the translational relevance of the functional proteome in research and clinical settings, we provide an online, publicly available, comprehensive database and visualization repository of our findings (https://mjha.shinyapps.io/PRECISE/).

## Introduction

Cancer is a complex disease initiated and characterized by myriad molecular and genomic events, which often occur within a modular and layered architecture of functional or cell signaling pathways^1–3^. These include perturbations of signaling pathways that generate modifications of molecular activities affecting the downstream molecules along specific pathways. The coordinated triggers that regulate the cumulative change in the signaling pathways may result in different phenotypic changes, along with signaling alterations that can be delineated by a deeper understanding of the pathway networks and topologies^4^. These network structures vary widely within and between different tumors and are important factors in characterizing molecular tumor types and developing therapeutic strategies, especially in cancer^5,6^.

Most systematic endeavors for investigating pathways can be broadly classified into two categories based on the hypothesis, approach and outputs: *global* (*across patients*) and *local* (*within patients)*. Global approaches typically generate population-level summaries (across patients) that rely on different distance metrics, such as functional and topological information of the networks, to measure associations between the target genes and known gene/protein sets of interest that represent various cellular processes. For example, methods based on gene set enrichment analysis^7–9^ use functional information that assesses the statistical overrepresentation of genes in a pre-selected list of interest from a reference list of known gene sets without consideration of their topological information in the graph structure. Analogously, several computational methods have been developed that incorporate the known topologic pathways to improve the prioritization of the gene set associations^10,11^. Without defining pathways, *de novo* pathway enrichment analysis^12–14^ has been proposed to identify functional modules/sub-networks enriched in biological entities active in a given experimental dataset by using large-scale interaction network.

The local category of patient-specific approaches has recently emerged within a precision medicine paradigm. It is the treatment and prevention of disease by accounting for individual variability to match the right drugs or intervention to the right patients^15^. This type of approach acknowledges the fact that pathway structures and activity may differ considerably within and between patients as well as across tumor sites. For example, patient-specific pathway or meta-gene (MG) analysis has been proposed to infer the patient-specific activity of a gene/pathway/MG by incorporating known (pathway) interactions among genes and multiple types of molecular data^16–18^ as well as the functional impact of a mutation within gene/protein networks^19–21^. However, these methods only incorporate curated (i.e., existing) interaction database and do not consider *de novo* data-driven causal networks. Moreover, these methods provide patient-specific scores of individual genes/MGs, as opposed to more interpretable cumulative pathway-level summaries that better reflect the complex pathophysiology of tumors.

In this study, we develop a novel and general Bayesian framework that conflates both global and local approaches: personalized cancer-specific integrated network estimation (PRECISE). PRECISE is a multi-scale approach that integrates multiple sources of information: *a priori* existing knowledge databases, data-driven *de novo* causal structures and diverse molecular profiling data at proteomic, transcriptomic (mRNA expression), epigenomic (DNA methylation) and microRNA expression levels. The novelty of our methodology lies in combining causal structure learning, Bayesian graphical models and variable selection at both population (cancer-specific) and patient-pathway levels. PRECISE outputs cancer-specific integrated networks, infers patient-specific networks (PRECISE networks), and elicits interpretable pathway-level signatures (PRECISE scores) by summarizing the patient-specific network as a single score that can be used for tumor stratification as well as constructing pathway-based prognostic models in cohorts of patients.

We delineate the interplay of proteins in signaling pathways in various cancer types and infer patient-specific proteomic pathway activities. While pathways have often been assessed at the DNA and RNA levels, proteomic signaling networks are emerging for two critical reasons. First, the amount and function of proteins are downstream summation of changes that occur at the genomic and transcriptomic levels^22^. Predictions of protein levels from RNA levels are weak (with correlations <=0.5), which can be explained by many factors, for example (i) suppression of translation by miRNAs, (ii) rate of degradation of mRNAs may be different from their protein products, (iii) phosphoproteins are dependent on signaling activity and may not correlate well with their transcripts alone, (iv) in the case of protein complexes, the presence or absence of other proteins in the complex may stabilize the complex or destabilize it resulting in altered protein expression^22^. Furthermore, because the signaling properties of proteins can be modified by post-translational events like phosphorylation, acetylation and ubiquitination, it is difficult to predict their functional impact at the genomic or transcriptomic level^23^.

Second, the signaling properties of proteins can be further modified by post-translational events e.g. through phosphorylation and other processes, which cannot be predicted at the genomic or transcriptomic level^23^. Thus, assessing the cancer-specific topology and structure of proteomic signaling networks and relating them to (prognostic) clinical outcomes are important tasks toward understanding the biological mechanisms behind cancer development and progression and, more importantly, identifying potential therapeutic targets^5^.

We applied PRECISE to a pan-cancer reverse-phase protein array (RPPA) proteomic data set (http://tcpaportal.org) of 7,714 samples from 32 tumor types from The Cancer Genome Atlas (TCGA) where-in we analyze multiple pathways of critical importance to the behavior of tumor cells and response to therapy. From the comprehensive exploration of proteomics-based patient-specific pathway signatures across 32 cancer types, we delineate pan-cancer commonalities and differences in key proteomic signaling pathway activities and prognostic power of the patient-specific scores. We demonstrate the superior performance of PRECISE algorithm as compared to other established and current approaches as well as potential clinical utility of our scores across tumor types. We believe that our study is the largest and most comprehensive network-based modeling and exploration of pan-cancer proteomic pathway activities for tumor stratification and prediction.

## Results

### PRECISE: a Bayesian integrative network model to construct cancer-specific and patient-specific networks/scores

**Figure 1:**
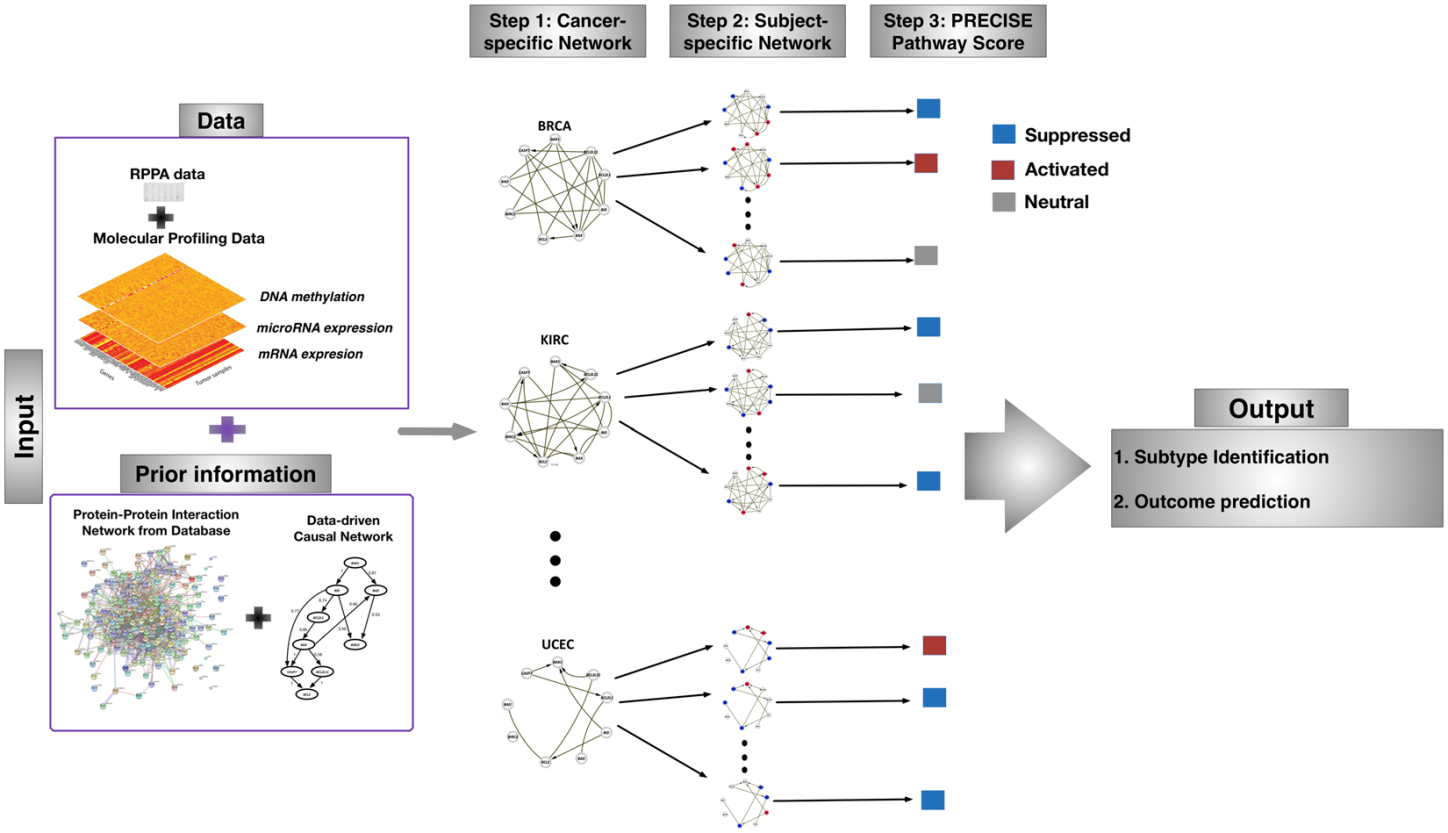
Overview of the Personalized Cancer-specific Integrated Network Estimation (PRECISE) method. PRECISE combines information from multiple platform molecular data and prior existing information from known protein-protein interaction databases. In Step 1, cancer-specific integrated protein networks are estimated by Bayesian regression models for each of signaling pathways using the prior networks and upstream molecular profiling data. In Step 2, PRECISE (subject-specific) networks are constructed by deconvolving proteomic network activity for each patient. In Step 3, PRECISE pathway scores are calibrated by summarizing each of the PRECISE networks for each pathway using the network topological information.

We developed a general Bayesian framework to estimate cancer-specific and patient-specific networks and elicit patient-level pathway scores by integrating data arising from multiple platforms and incorporating existing network information. With Pan-cancer TCGA proteomic, genomic and epigenomic data, PRECISE uses a multi-scale approach (Fig. 1). The implementation of PRECISE on proteomic networks only requires two inputs: (1) data — proteomic data and other (upstream) molecular profiles (e.g., gene expression, microRNA expression, DNA methylation) and (2) prior information — known pathway annotations and PPIs. Briefly, in the first step of PRECISE, a protein-protein causal network is estimated and combined with the prior information (from PPI’s). Other upstream molecular data are then integrated in three sequential steps to produce cancer-specific, patient-specific networks (cancer-specific network with patient-specific node labels) and pathway scores (PRECISE scores), which are subsequently used for tumor subtype classification and clinical outcome prediction.

### Rewiring *de novo* protein signaling pathways across 32 tumor types

We applied PRECISE independently on 32 cancer types from TCGA, which are listed in Table 1. The largest and smallest tumor types were breast cancer (n = 878) and uveal melanoma (n = 12), respectively. We combined colon and rectal tumors into one based on the result that the overall patterns of changes in copy number, CpG island methylator phenotype (CIMP), mRNA and miRNA were indistinguishable between the two tumors^24^. We investigated 12 pathways of critical importance to the behavior of tumor cells and response to therapy: apoptosis, breast reactive, cell cycle, core reactive, DNA damage response, EMT, PI3K/AKT, RAS/MAPK, RTK, TSC/mTOR, hormone receptor, and hormone signaling (breast) as described in the previous TCGA RPPA study for 11 tumor types^22^. The gene/protein membership for each pathway is listed in Supplementary Table S1. For each cancer–pathway combination, we applied our PRECISE method to infer the integrated cancer-specific network and their connectivity. We investigated two axes of information for each combination: *pan-cancer network signaling and rewiring*.

**Table 1:** TCGA tumor types and sample sizes in RPPA data (alphabetical order).

Code	Disease (full name)	Sample size
ACC	Adrenocortical carcinoma	46
BLCA	Bladder Urothelial Carcinoma	343
BRCA	Breast invasive carcinoma	878
CESC	Cervical squamous cell carcinoma and endocervical adenocarcinoma	171
CHOL	Cholangiocarcinoma	30
CORE (COAD + READ)	Colon/Rectum adenocarcinoma	491
DLBC	Lymphoid Neoplasm Diffuse Large B-cell Lymphoma	33
ESCA	Esophageal carcinoma	126
GBM	Glioblastoma multiforme	232
HNSC	Head and Neck squamous cell carcinoma	203
KICH	Kidney Chromophobe	63
KIRC	Kidney renal clear cell carcinoma	469
KIRP	Kidney renal papillary cell carcinoma	217
LGG	Brain Lower Grade Glioma	432
LIHC	Liver hepatocellular carcinoma	184
LUAD	Lung adenocarcinoma	362
LUSC	Lung squamous cell carcinoma	325
MESO	Mesothelioma	61
OV	Ovarian serous cystadenocarcinoma	431
PAAD	Pancreatic adenocarcinoma	122
PCPG	Pheochromocytoma and Paraganglioma	82
PRAD	Prostate adenocarcinoma	351
SARC	Sarcoma	224
SKCM	Skin Cutaneous Melanoma	355
STAD	Stomach adenocarcinoma	392
TGCT	Testicular Germ Cell Tumors	122
THCA	Thyroid carcinoma	380
THYM	Thymoma	90
UCEC	Uterine Corpus Endometrial Carcinoma	439
UCS	Uterine Carcinosarcoma	48
UVM	Uveal Melanoma	12
Total		7714

#### Pan-cancer network signaling

We investigated the extent of cross-signaling (among proteins) for each pathway across lineages. For defining a metric of connectivity in the corresponding integrated cancer-specific network, we use *connectivity score* (CS), which is the constructed as the ratio of the observed number of edges in the network to the total number of possible edges and its permutation p-value (${p}_{cs}$) defined in Section S1.5 (Supplementary Table S2). PRECISE provided robust cancer-specific integrative networks based on the resampling-based validation (Section S1.6). We computed *diversity score*, which is defined by standard deviation of CS values across lineages (Supplementary Fig. S22A). The high diversity score indicates that the levels of connectivity of a given pathway are different across tumor types: the proteins in a pathway are highly connected in some cancers but have few connections in other cancers. The diversity score ranged from 0.10 to 0.29. Importantly, the CS was markedly different in different lineages, not unexpectedly with the greatest variability (diversity score = 0.29) in the hormone signaling breast pathway that captures aspects of hormone response in breast cancer. Surprisingly in contrast, the apoptosis and DNA damage response pathways demonstrated similar levels of connectivity across almost all cancer lineages with diversity scores, 0.10 and 0.11, respectively. Briefly, the CS for the breast reactive pathway was 0.8 with ${p}_{cs}=0.062$ in breast cancer (BRCA), which suggests that the data-driven cancer-specific network aligns well with the *a priori* functional characteristics of this pathway. Analogously, the CSs for the core reactive pathway that was defined on the basis of all lineages were high (>0.7) in multiple lineages including colon/rectum cancers (CORE), BRCA, and thyroid cancer (THCA) while only CORE samples provided its significance with ${p}_{cs}=0.038$. We also found a high level of cross-signaling in the RTK and TSC/mTOR pathways in BRCA, with 0.9 CSs (${p}_{cs} < 0.02$). Among the 12 pathways, Cell cycle, TSC/mTOR and Hormone receptor signaling pathways showed the most significant levels of cross-signaling across cancer types- Hormone receptor pathway showed significance across 13 cancer sites including kidney cancer (KIRC), lung cancers (LUAD, LUSC), CORE, PAAD, STAD, LIHC, MESO, UCS, OV, LGG, ESCA, and DLBC.

#### Pan-cancer network rewiring

**Figure 2:**
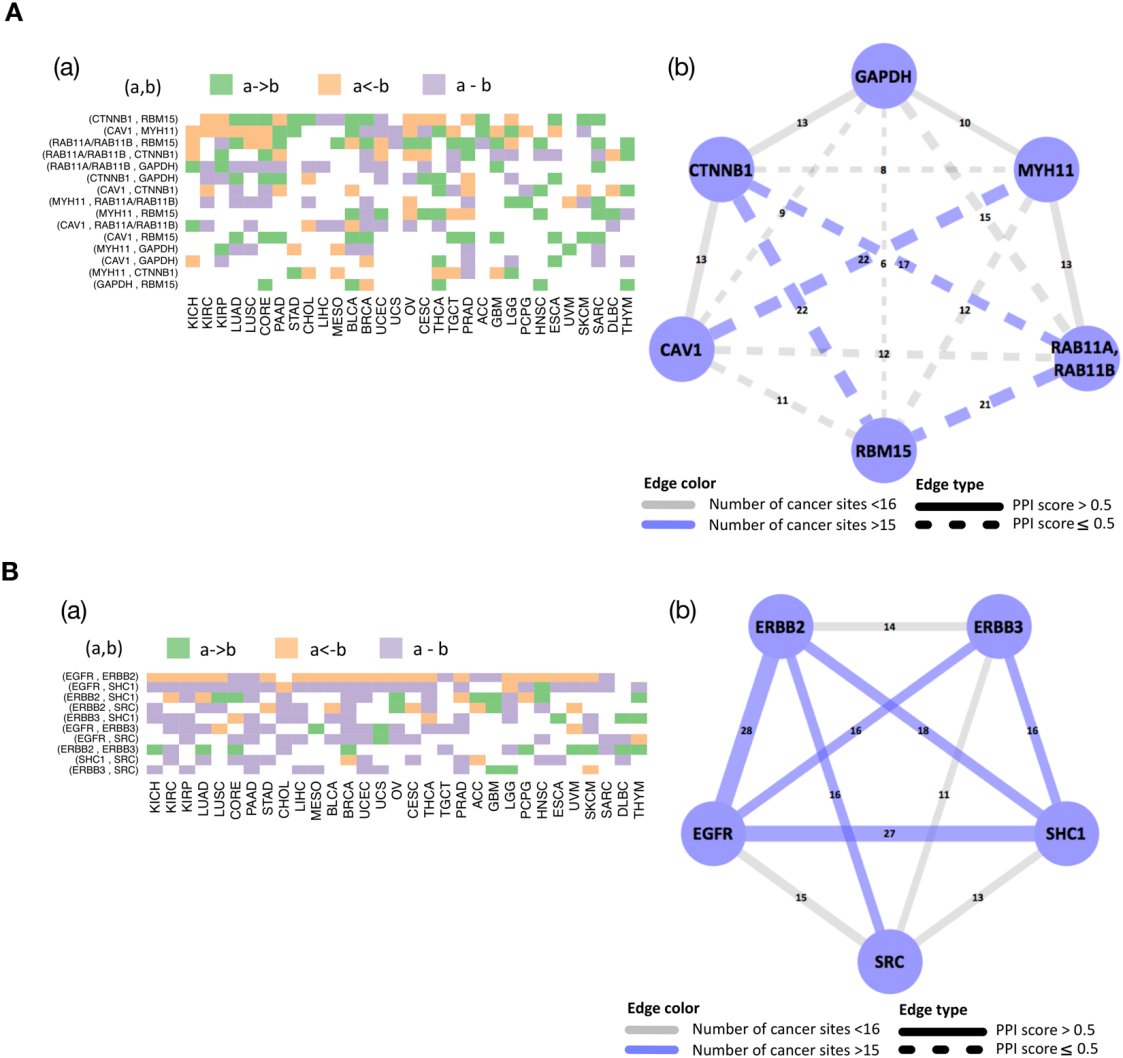
Cancer-specific protein networks for breast reactive (**A**) and RTK (**B**) pathways. (a) Heatmap depicting regulatory (−> or <−) and correlative (−) edges across all tumor lineages. (b) Network, where each of the edges is weighted and labeled by edge consistency (EC), defined as the number of tumor types that hold the particular edge.

A pan-cancer analysis was undertaken to determine the extent to which edges (both regulatory and correlative) in each of the pathways are shared across tumor types. To this end, we used the *edge consistency (EC)*, which for a given edge is defined as the number of tumor types that hold the particular edge. We compared the EC to the known protein-protein interaction (PPI) score to corroborate with existing evidence as well as find new ones. Figure 2 and Supplementary Figs S1–S5 display heat maps for edges across all cancer sites (panel (a)) and networks, where each of the edges is weighted and labeled by the EC (panel (b)). Overall, the frequencies of edges across tumor sites and the PPI scores were consistent in that high edge frequency tended to be associated with PPI score greater than 0.5 (e.g. RTK pathway in Fig. 2B-(b)). However, some edges that had high frequencies had PPI scores less than 0.5 (e.g. dashed blue edges in breast reactive pathway in Fig. 2A-(b)) suggesting that there are intermediary molecules in the signaling pathway that are however, not rate limiting. All the 12 pathways had links that are shared across more than 20 lineages and those findings are classified into known (PPI score > 0.5) and new (PPI score ≤ 0.5) findings (Supplementary Table S3). For example, the breast reactive pathway is evaluated on the basis of TCGA RPPA breast cancer data, and all highly shared edges are new findings. We found that this breast reactive network for breast cancer patients has the highest number of edges, which is along expected lines but was markedly diverse in other cell lineages (Fig. 2A-(a)). Interestingly, we also found highly conserved directions among known edges with PPI score > 0.5: the regulatory edges CCNB1->CCNE1 in cell cycle pathway (Supplementary Fig. S1B), AKTS1->AKT1/AKT2/AKT3 in PI3K/AKT pathway (Supplementary Fig. S3B), MAPK8->JUN in RAS/MAPK pathway (Supplementary Fig. S3A), ERBB2->EGFR (Fig. 2B), PGR->ESR1 in hormone receptor pathway (Supplementary Fig. S2A) were conserved for more than 18 lineages.

### Robust network-based Pan-cancer stratification using PRECISE

**Figure 3:**
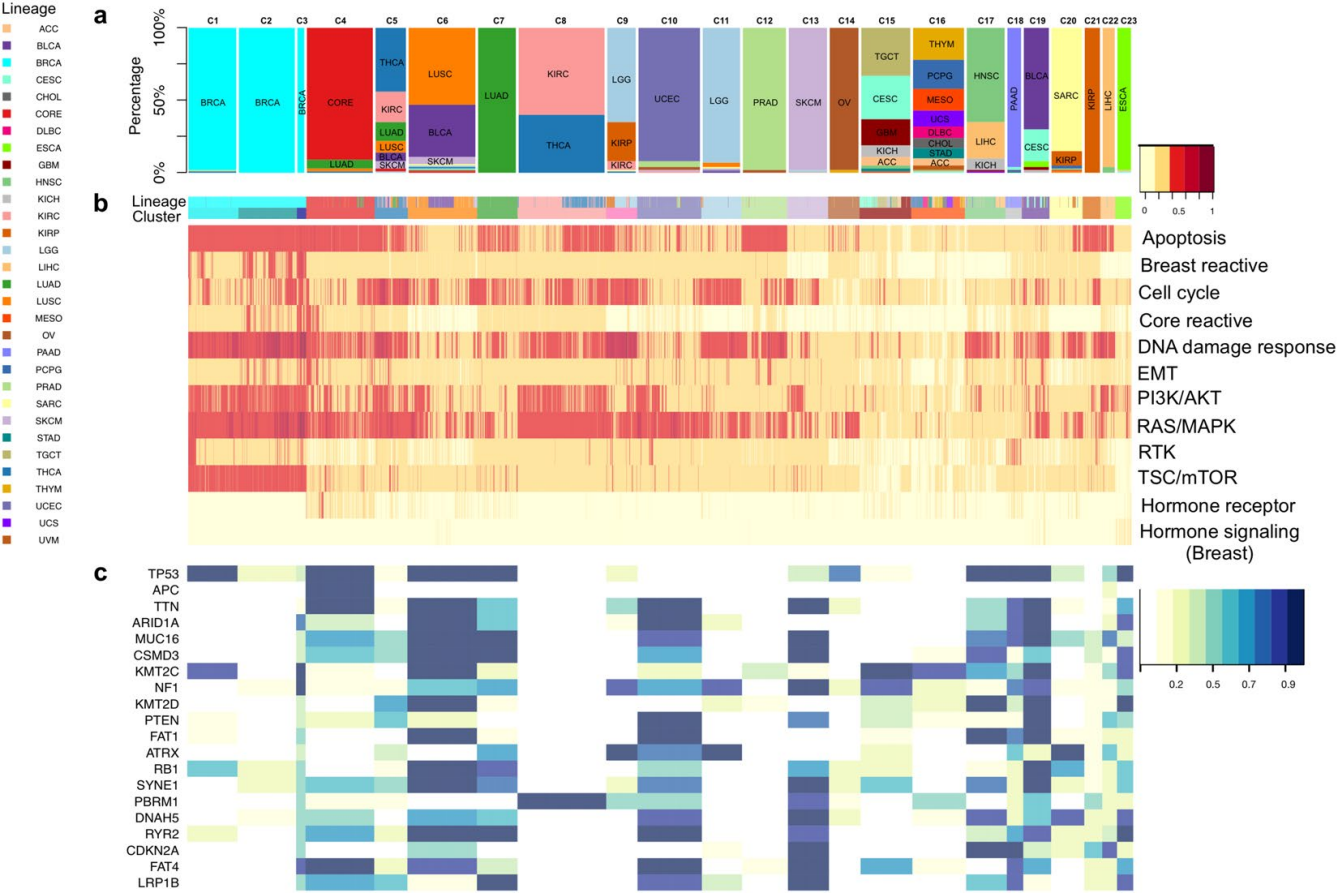
Network-based subtype stratification using PRECISE. (**a**) Proportions of tumor types represented in each cluster. (**b**) Heatmap depicting combined activated and suppressed PRECISE scores after unsupervised hierarchical clustering of the score matrix consisting of 6,844 patients across all cancer lineages and 12 proteomic signaling pathways. The scores are indicated on low-to-high scale (yellow-to-red). 23 clusters are defined. Annotation bars include tumor types. (**c**) Heatmap depicting enrichment probabilities (EP) of mutations for most frequently mutated 20 genes (across all clusters). The probabilities are indicated on low-to-high scale (yellow-to-blue).

To identify subtypes for the pan-cancer patients and pathway determinants of those subtypes, we developed a tumor stratification algorithm for all of the tumor samples in the 32 lineages based on the PRECISE scores. The PRECISE scores were computed by deriving summary measures from the PRECISE networks (patient-specific), indicating that the entire pathway is suppressed, neutral or activated. For the clustering analysis, we combined the two positive values for the suppressed and activated scores by summation- the combined score for a pathway and patient- thus represents a global perturbation level including activated or suppressed directions of the pathway for the patient. After matching all four platforms, RPPA, mRNA expression, microRNA expression, and DNA methylation, we had 6,844 patients across the 32 lineages. For all patients and all pathways, we constructed a 6,844 × 12 suppressed and activated PRECISE score matrix, with each row corresponding to a patient and each column corresponding to a pathway. This matrix was the input for our clustering analysis, which identified 23 optimal clusters of patients (Fig. 3, Supplementary Table S4 and Supplementary Fig. S6). We conclude that core signaling pathways show similar activity levels for patients with the same tumor origin and known molecular subtype within cancer. We found a high degree of concordance between the PRECISE clusters and tumor types, suggesting that the intrinsic pathway activity patterns in a given lineage are key drivers of the PRECISE scores (Fig. 3(a,b)). Note that the unsupervised clustering analysis on the raw RPPA and mRNA expression data showed decreased tissue-specific signals (Supplementary Figs S7 and S8) with normalized mutual information (NMI) scores (Section S1.7) of 0.63, 0.70, and 0.81 for mRNA, RPPA, and PRECISE, respectively (Section S1.7). Supplementary Figs S27–S57 display the combined activated and suppressed scores for each of the cancer types. We further investigated the enrichment of the 23 clusters to the mutational profiles, and known clinical subtypes.

#### Concordance with known molecular subtypes of breast cancer

**Figure 4:**
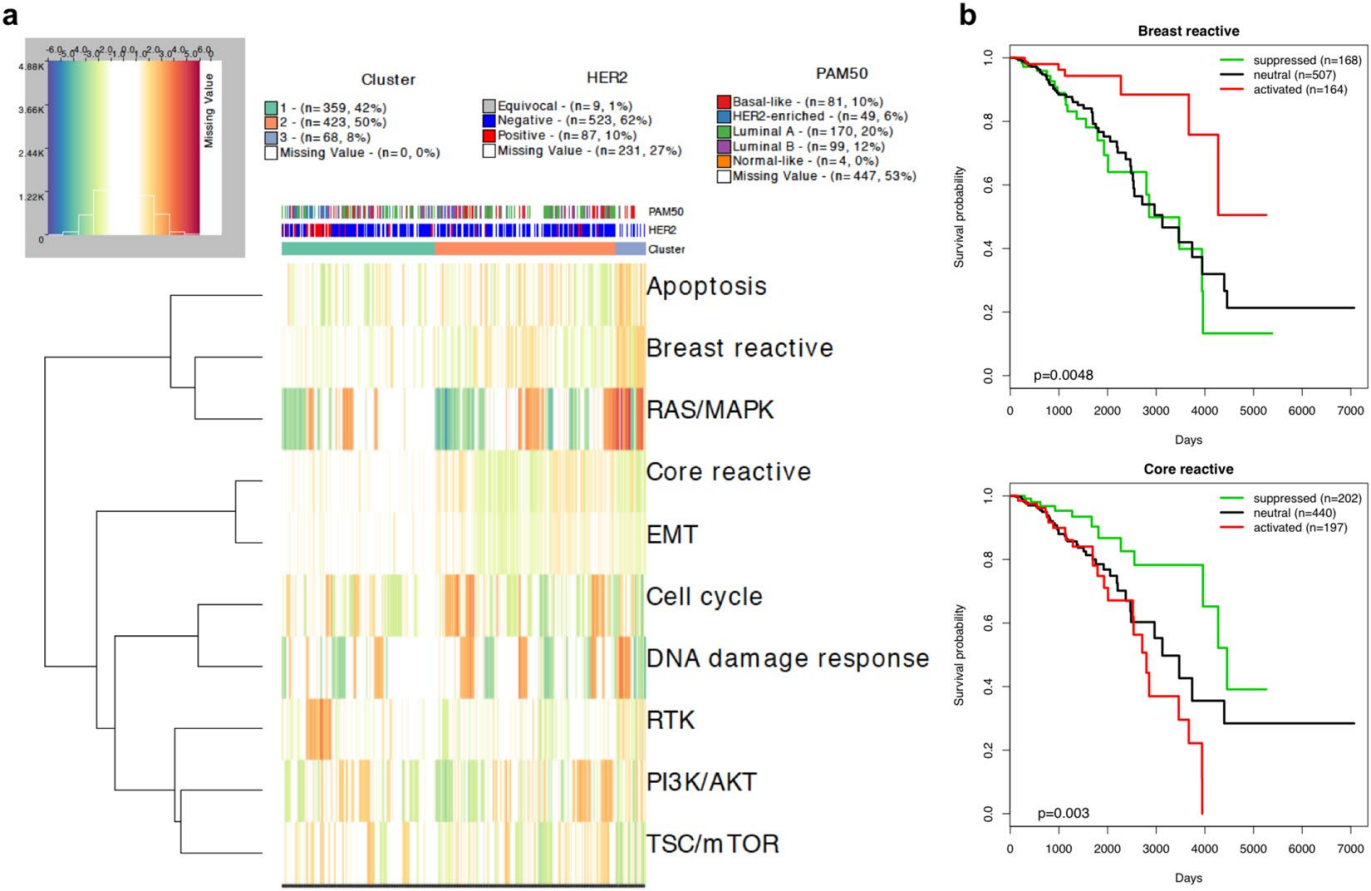
Concordance with known clinical subtypes of breast cancer (BRCA). (**a**) Heatmap depicting combined activated and suppressed PRECISE scores across 878 BRCA patients located in clusters C1, C2, and C3. The combined scores are signed by PRECISE statuses, -1 (suppressed), 1 (activated), or 0 (neutral). The signed scores are indicated on a suppressed-neutral-activated scale (blue-white-red). (**b**) Kaplan-Meier curves for the three groups of BRCA patients according to the PRECISE statuses, suppressed (green), neutral (black), and activated (red) for breast reactive and core reactive pathways.

The samples of BRCA were almost exclusively located in clusters C1, C2 and C3 (Fig. 3a,b). To further establish the robustness of the clusters, we computed the *enrichment probability (EP)*, which is defined as the posterior probability of enrichment in patients within a particular clinical subtype (e.g., breast cancer subtypes according to ER, PR and HER2 statuses) in a given cluster, with EP close to 1 if the proportion of patients from the subtype in the cluster is significantly higher than the proportion of patients from outside the subtype in the cluster by random chance (EP close to 0). The detailed information on computing EP is in Section S1.3. The DNA damage response and RAS/MAPK pathways were highly activated or suppressed for all BRCA samples, and most samples showed activation or suppression for the apoptosis, PI3K/AKT, and TSC/mTOR pathways (Supplementary Table S5). Cluster C1 includes 80% (EP = 1) of the HER2 protein-enriched breast cancers and 74% (EP = 1) of HER2-positive breast cancers. Of the patients with HER2-positive breast cancer in cluster C1, 75% showed activation of the RTK pathway that includes the HER2 (ERBB2) protein (Fig. 4a). Furthermore, we also confirmed that HER2 + BRCA samples (as determined by IHC) have high PRECISE RTK scores compared to other BRCA samples (Supplementary Fig. S10). Cluster C2 includes 60% (EP = 0.99) of patients with the basal-like breast cancer subtype, 50% (EP = 0.61) of those with luminal A subtype, and 52% (EP = 0.71) of those with luminal B subtype. Cluster C2 also includes 53% (EP = 1) of the HER2-negative breast cancers. The cell cycle pathway was activated for 78% of the patients with basal-like breast cancer in C2 (Fig. 4a). More importantly, although the C3 cluster is small (n = 68), all the samples clustered in C3 represented HER2-negative breast cancer (with EP = 0.99). In addition, the samples in cluster C3 showed enrichment for the basal-like and luminal A subtypes, with EP = 0.97 and 0.72, respectively. Moreover, most BRCA samples in C3 had high scores in all pathways, with the exception of the RTK, hormone receptor, and hormone signaling (breast) pathways (Supplementary Table S5). More specifically, 66%, 82%, and 72% of the samples in C3 showed activation of the apoptosis, breast reactive, and RAS/MAPK pathways, respectively. Suppression of the core reactive, EMT, and TSC/mTOR pathways was respectively found in 75%, 60%, and 55% of the samples in cluster C3 (Fig. 4a). Together, this indicates that the network-based clustering approach captures the distinct information content in breast cancer compared to other standard clustering approaches, evidenced by unsupervised hierarchical clustering results based on mRNA data and RPPA data only (see Supplementary Figs S7 and S8, and Section S1.1). This is emphasized by the marked differences in patient outcomes between the three clusters (p = 0.0046), with patients associated with C1, C2, and C3 having 5-year survival rates (95% confidence interval) of 0.71 (0.62–0.82), 0.84 (0.78–0.92), and 0.96 (0.91–1), respectively. In contrast, the clustering pattern based on raw RPPA data and mRNA expression data have not such prognostic power (Supplementary Figs S7 and S8): the top three BRCA clusters using RPPA data in terms of sample sizes, C15 (n = 555), C8 (n = 96), and C7 (n = 68) had 5-year survival rates (95% confidence interval) of 0.81 (0.74–0.87), 0.72 (0.58–0.88), and 0.83 (0.66–1), respectively with p-value of 0.2679, and the top three BRCA clusters using mRNA expression data, C14 (n = 548), C9 (n = 111), C3 (n = 57) had 5-year survival rates (95% confidence interval) of 0.80 (0.73–0.88), 0.74 (0.61–0.90), and 0.91 (0.80–1), respectively with p-value of 0.0862 (Supplementary Fig. S9).

In summary, we focus on constructing pathway-based prognostic index and test for its potential prognostic value in breast cancer, as opposed to METABRIC study^25^ that obtained biological subgroups by using integrative clustering based on CNA and gene expression data and then projected the molecular profiles of the subgroups onto pathways to examine possible biological themes among breast cancer subgroups.

In other tumor types, the kidney renal clear cell carcinoma (KIRC) and thyroid carcinoma (THCA) samples were both located primarily in clusters C5 and C8 (Fig. 3a,b). KIRC metastases to the thyroid gland have been reported in the literatures^26–28^. Most samples of KIRC and THCA in C5 and C8 showed high levels of activation or suppression of the apoptosis, cell cycle, DNA damage response, PI3K/AKT, and RAS/MAPK pathways (Supplementary Table S5). In particular, differential activation of PI3K/AKT and RAS/MAPK pathways was reported in the THCA study^29^. However, samples in cluster C5 showed stronger signals than those in C8, especially for the cell cycle and DNA damage response pathways (Supplementary Fig. S11). Low-grade glioma (LGG) was located in 8 clusters, with C9 and C11 as the main clusters and including 145 and 269 samples, respectively (Fig. 3a,b). Almost all LGG samples in C9 and C11 showed high levels of activity in the cell cycle, DNA damage response, and RAS/MAPK pathways (Supplementary Table S5). The LGG samples in C9 showed activity in the apoptosis pathway, which discriminates the LGG samples in cluster C9 from those in cluster C11.

#### Concordance with mutational analyses

We further investigated the relationship of the PRECISE-based clusters to the mutation status of known cancer genes. For each of the most frequently mutated 20 genes (across all clusters found by PRECISE), we computed the enrichment probability (EP), which is defined as the posterior probability that the proportion of samples with a mutation in a cluster is higher than the proportion of samples with a wild-type modification in the cluster (Fig. 3c). The most frequently mutated gene across all clusters was TP53, and its mutations were enriched only in C1 among the clusters for BRCA (EP > 0.9). APC was the second most commonly mutated gene, and was mostly located in CORE samples in C4 (82%) with EP > 0.9 (Fig. 3c and Supplementary Table S6). More importantly, the PBRM1 gene was reported as a novel target for KIRC^30^ and, in our study, the mutations were found in KIRC and THCA samples in C8 (60%), compared to no mutations in KIRC and THCA samples in C5 (Fig. 3c). This indicates that our clustering method based on PRECISE effectively classified subtypes across cancers.

### Prognostic utility of PRECISE scores

**Figure 5:**
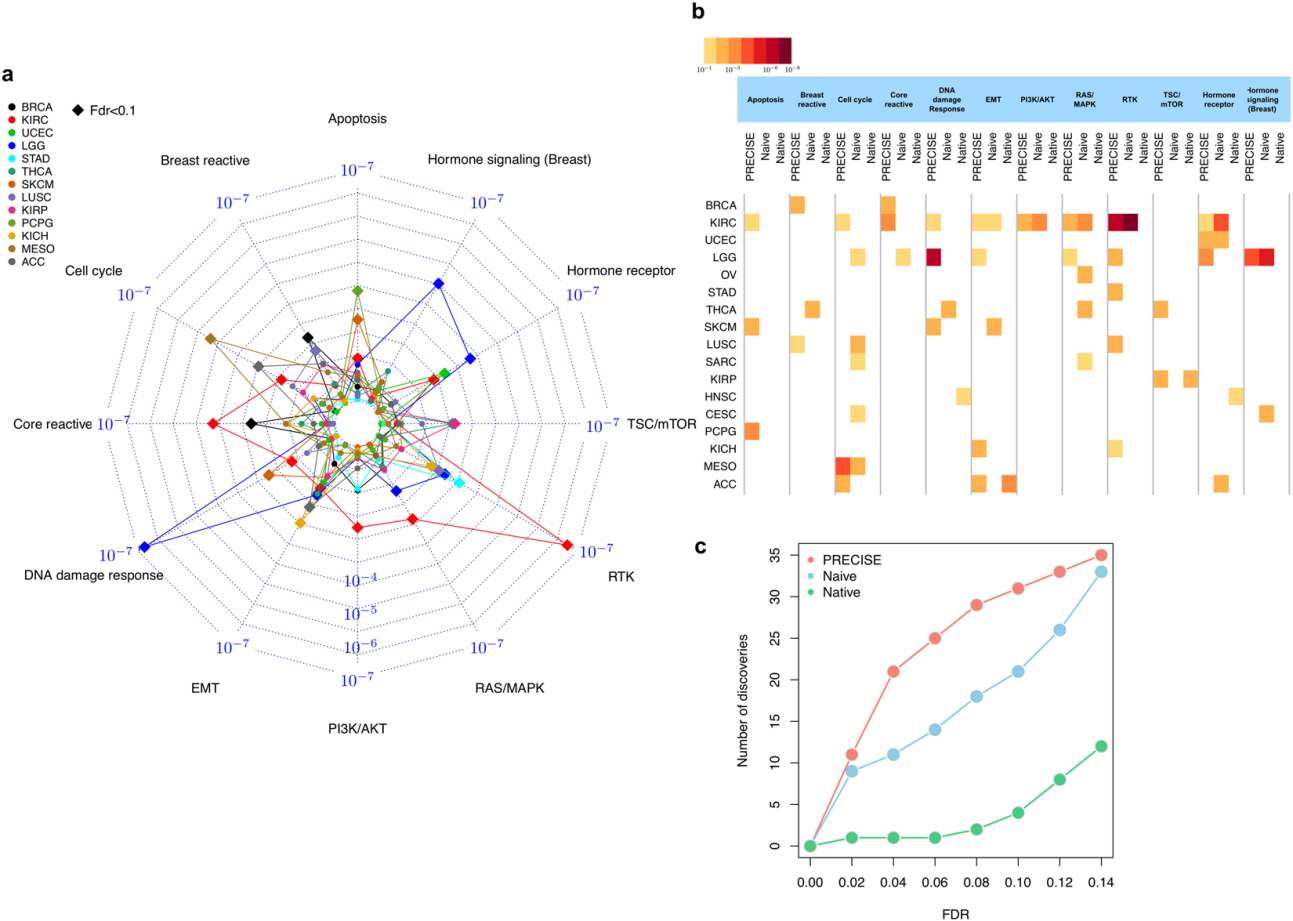
Supervised learning with survival times based on PRECISE scores. (**a**) Radar plot for p-values for PRECISE statuses (suppressed, neutral, or activated) using log-rank test for survival times. (**b**) Heatmap for p-values (high-to-low) with FDR < 0.1 across all 17 cancer types that provides p-values with FDR < 0.1 for at least one pathway and all the pathways, and three different methods, PRECISE, naïve and native. (**c**) The number of discoveries for the three different methods according to various FDR cutoffs.

We further investigated the prognostic potential of the PRECISE framework on clinical outcomes, namely, the survival times of patients in unsupervised manner. Our PRECISE method provides three categories of PRECISE scores, the probability of suppression, neutral effect, and activation of the 12 pathways for each patient and each pathway. Thus, for a given pathway and each patient, the *PRECISE status*—which indicates that the pathway is suppressed, neutral, or activated for the patient, can be decided by the maximal probability. To investigate the prognostic power of the PRECISE status, we performed Kaplan-Meier survival analysis with a log-rank test to determine the difference in survival probabilities among the three groups according to the PRECISE statuses. The direction of the association with PRECISE statuses varied in different tumors, indicating that the effects of the pathways were dependent on the intrinsic characteristics of the tumor, either in terms of overall prognosis or intrinsic proteomic pathway expression patterns (Fig. 5a). Some key results are highlighted below. Among patients with KIRC, those for whom the RTK pathway showed activation had better survival times than those for whom the pathway showed suppression (Supplementary Fig. S12). The PRECISE status for the DNA damage response pathway was the most predictive for LGG: the patients who had suppressed PRECISE status in the DNA damage response pathway had worse survival than those who showed neutral or activated statuses (Supplementary Fig. S13B). We hypothesize that the suppression of the DNA damage response pathway occurs through the activation of MDM4, which was shown to be activated in the IDH wild-type subtype of LGG^31^. Patients with that subtype had poor survival and the tumor sample resembled GBM from a molecular perspective, which shows low DNA damage activity (Fig. 3b). Patients with BRCA had better survival when the breast reactive pathway was activated, as previously reported^22^ and the core reactive pathway was suppressed (Fig. 4b,c). The cell cycle pathway showed strong prognostic power among patients with mesothelioma (MESO): they showed better survival when the level of suppression increased (Supplementary Fig. S13A). The very strong association with prognosis suggests that these pathways play a major role in the clinical outcomes of patients with these diseases, and that targeting specific pathways in different tumors may improve patient outcomes.

To further demonstrate the potential clinical utility of our PRECISE scores, we investigated the role of Epithelial-mesenchymal transition (EMT) and Receptor tyrosine kinases (RTK) pathways – which have established and critical roles in cancer development and metastasis across multiple tumor type. We correlated PRECISE EMT activated/suppressed score with epithelial tumor types such as Ovarian (OV), Lung Adenocarcinoma (LUAD), and Stomach Adenocarcinoma (STAD), and mesenchymal tumor types such as brain tumors, Glioblastoma Multifome (GBM) and Low Grade Gliomas (LGG). The mesenchymal tumor types such as brain tumors, GBM and LGG tend to have higher PRECISE EMT scores than the epithelial tumors such as OV, LUAD and STAD (Supplementary Fig. S14).

### External and internal validation of PRECISE pathway signatures

We extensively validated the prognostic potential of PRECISE using internal and external validation strategies within and across multiple cancer types, existing methods and its prospective prediction performance.

#### Internal validation

We used a diluted version of our PRECISE method without using prior calibration from existing databases and *de novo* causal network, and computed the pathway scores across the 12 pathways for BRCA patients. We used breast cancer since they showed the clearest patterns of prognostic proteomic signatures (as shown before) and thus enable a fair comparison. Using prior knowledge from protein causal structure learning and existing database helps to improve its prognostic power for most pathways. The difference was the largest in the breast reactive pathway that were defined based on the breast cancer patients: 14 folds increase in p-values when we use no prior (Supplementary Fig. S15). We also analyzed the BRCA data after we left the upstream platforms out from the PRECISE algorithm and without prior information. The procedure provided empty networks with no edges for the Hormone receptor and Hormone signaling pathways. On the other hand, PRECISE algorithm provided integrated networks (Supplementary Fig. S16). Integration procedure of PRECISE in the network estimation helps to calibrate patient-specific pathway signatures by borrowing strength across multiple platforms and prior knowledge when the pathway is small and the correlations in RPPA data are weak within pathways. We also observed superior performance of the PRECISE network based clustering algorithm to detect tumor-specific signals to that of the unsupervised clustering method on RPPA and mRNA expression data only (Supplementary Figs S7 and S8). We computed normalized mutual information (NMI) scores to evaluate the dependence between clusters and tumor types (Section S1.7). The mRNA clusters provided NMI score of 0.63 that is the lowest value compared to 0.70 for RPPA clusters and 0.81 for PRECISE clusters. PRECISE showed the highest level of tumor specificity.

#### External validation

We compared the prognostic potential of PRECISE with that of published/random pathway scores and metrics. We considered four pathway scores: (1) *naïve pathway score*, which is defined as the cumulative sum of all protein expression in a particular pathway, (2) *native pathway score*, which is defined as the sum of all positive regulatory components minus that of the negative regulatory components in a particular pathway as used and reported by recent literatures^22,32,33^, (3) *PARADIGM score*, which measures the patient-specific genetic activities incorporating curated pathway interactions among genes using expression^16^, and (4) Random protein signatures obtained by the method following^34^ (see Section S1.1). The naïve method gives all proteins equal weight (thus ignoring network information) in estimating the pathway-level score; whereas the native method takes directionality (+/−) into account to yield overall pathway-level scores. PARADIGM uses pathways from the National Cancer Institute Pathway Interaction Database (NCI-PID) and assigns an integrated pathway level (IPL) reflecting the activity of each node by computing patient-specific log-likelihood ratio. The major difference between these methods and our PRECISE algorithm is that we estimate and construct *de novo* cancer-specific networks (model building) to calibrate the patient-level pathway signatures by exploiting multiple sources of information obtained from cancer-specific multi-platform data, causal structure learning and existing interaction database. It is well established that sub-networks within signaling pathways and their products undergo changes in response to different conditions^4,6,35^. Such *network rewiring* in cancers are manifested at the level of signaling networks and is not currently well understood^5^. For FDR < 0.1, PRECISE, naïve and native scores were prognostic for 31, 21, and 4 numbers of tests across all the 12 pathways and lineage combinations (Fig. 5b). There were 21 pathways across cancer types for which only the PRECISE score was predictive of survival times: for example, patients with KIRC, SKCM, and PCPG for the apoptosis pathway. Overall for different cutoffs of FDR, our PRECISE method had superior prognostic power in predicting survival times as compared to the naïve and native signatures (Fig. 5c). For KIRC patients, we evaluated the prognostic power of PARADIGM scores (Section S1.1) and PRECISE performed better than PARADIGM to predict patients’ survival times in unsupervised manner (Supplementary Fig. S17).

Next, we compared the PRECISE scores with the native and PARADIGM scores in the same context of the recent paper^32^ across four tumor types. UCEC, OV, SARC, and UCS – to assess shared/differentiated pathway-based features. There was clear evidence that our PRECISE method preserves tumor-specific signals (94% of UCS, 99% of UCEC, 99% of SARC and 98% of OV samples were separately clustered) better than native and PARADIGM scores and showed more evident pattern of activities across different tumor types (Supplementary Figs S18, S19 and Section S1.1).

Finally, we compared the survival outcome association of PRECISE statuses to that of 1000 random protein signatures constructed from the first principal component of randomly selected proteins of identical size to the number of proteins in a given pathway (Section S1.1). We use the proportions of random signatures that work better than those obtained from PRECISE as another measure of significance; we refer those proportions as *p-values from random signatures*. At the cutoffs of the p-values, 0.1 and 0.05, we found 39 and 23 pathway-based signatures respectively across all 31 tumor types. The two approaches by controlling FDR and by using random signatures provide distinct information on the prognostic power (Supplementary Table S8). Using this random signature approach, there were additional new findings: for example, signatures of Core reactive, TSC/mTOR and Hormone receptor pathways were selected for a rare cancer, Lymphoid Neoplasm Diffuse Large B-cell Lymphoma (DLBC), that were not found by the FDR-adjustment (Supplementary Fig. S21 and Supplementary Table S8).

#### Prospective predictive performance

To predict a patient-specific protein expression, PRECISE uses distinct sources of upstream information, known PPI, data-driven regulators, and other molecular profiling data. We further evaluated the prediction accuracy of the protein expression level using the PRECISE framework compared to the observed protein levels. In order to verify the accuracy of protein prediction by the PRECISE method, we defined the *concordance score* between the predicted value using the PRECISE method and the observed value for each protein in a given sample (Section S1.4). This score takes values between 0 and 1, with a high value indicating that the predicted value has concordance with the observed value. Almost all lineages had concordance scores greater than 0.9; however, uveal melanoma (UVM) had large variations below 0.9, which was likely due to the small sample size of 12 (after matching all four platforms, RPPA, mRNA expression, microRNA expression, and DNA methylation) (Supplementary Fig. S22B). Thus, integrating information from multiple molecular levels is superior to the predictive utility of protein levels alone.

## Discussion

We develop a general Bayesian framework, called PRECISE, for inferring cancer-specific network structures and topologies of signaling pathways and inferring patient-specific pathway activities. We report the discovery that, through model-based integration of multiple sources of information such as existing protein-protein interactions databases, data-driven *de novo* molecular networks, and diverse upstream molecular profiling data, we can delineate pan-cancer commonalities and differences in network biology, robustly stratify tumors that are both biologically and clinically informative, and have better prognostic and predictive performance as compared to other standard approaches that do not incorporate these distinct axes of information. PRECISE provides an accurate and principled quantification of proteomic networks in a manner that is more robust, efficient and powerful than existing approaches. Our work fills a major gap in the literature which lacks holistic and coherent methodology to (1) explain tumor-specific pathway networks and connectivity; (2) develop patient-specific pathway networks and scores that are reflective of the molecular underpinnings in various tumor types; (3) are well-calibrated to identify tissue-specific signals, prognostic and clinical utility across multiple tumor types and; (4) general enough to be applied to any modern high-throughput technology that generated complex multivariate data.

Our method is motivated by and applied to a pan-cancer RPPA-based proteomic and genomic data set from TCGA samples across 32 tumor types from TCGA, where-in we investigate multiple pathways of critical importance in tumor initiation and progression. We reconstruct the networks relationships, identifying both regulatory (causal/directional) and correlative (non-directional) edges between the proteins in the pathways. Through Bayesian sparse regression modeling, our approach is able to produce sparse tissue-specific and pathway-specific networks and the incorporation of *a priori* knowledge leads to sparser and more biologically interpretable edges. We found a high degree of variation in pathway activity across cancer types, with some signaling pathways showing higher connectivity in certain tumor types than others (e.g., the breast reactive pathway showed high connectivity in breast cancer), which suggests that the data-driven cancer-specific network aligns well with the *a priori* functional characteristics of this pathway. The RTK pathway and the TSC/mTOR pathway showed high cross-signaling across most major tumor types. The pan-cancer analyses of edges to determine network relationships (both regulatory and correlative) revealed that several network/edge relationships were highly conserved across tumor types, some of which confirmed existing knowledge while others represented novel links with significantly high probability which might warrant further experimental and functional validation (Fig. 2, Supplementary Figs S1–S5 and Supplementary Tables S2, S3).

Using Bayesian posterior predictive modeling, we de-convolve the patient-specific networks from the global tissue-specific ones and derive network-based scores reflective of the underlying patient-specific pathway-level activities. Using these PRECISE scores in an unsupervised clustering algorithm to assess cross-tumor heterogeneity across all tumor types, we find a high fidelity between the optimal clusters and the tumor lineages, e.g., 23 clusters, 14 of which were enriched with single type of tissue at the site of tumor origin with markedly different pathway activation status, which can help delineate pathway-based therapeutic targets. We found three clusters that represent the 3 subtypes of breast cancer, luminal, HER2 positive and basal, and both of the three clusters and pathway scores for breast reactive and core reactive pathways were significantly associated with patients’ survival times. Patients who have KIRC and THCA tumors were mostly classified into two PRECISE clusters and the PBRM1 gene that is a novel drug target for KIRC was found in patients located in one of the clusters, which indicates that PRECISE effectively classified subtypes across cancers.

We also investigated different aspects of PRECISE by performing external and internal validations and showing potential clinical utility. For objective external validation of the PRECISE algorithm, we compared PRECISE scores with that of published as well as scores obtained by random (unstructured) perturbations of the data. We found clear evidence that our PRECISE method preserves tumor-specific and clinical subtype-specific signals better than other existing methods and has much higher prognostic power in detecting pathway-specific prognostic signals between and across tumor types as compared to existing methods and random signatures. For the internal validation, we used a diluted version of our PRECISE method without using prior calibration from existing databases and *de novo* causal networks, and computed the pathway scores across the multiple pathways for breast cancer patients that showed the clearest patterns of prognostic proteomic signatures and thus enable a fair comparison. We find that using prior knowledge from protein causal structure learning and existing database helps to improve its prognostic power for most pathways. The difference was the largest in the breast reactive pathway that were defined based on the breast cancer patients: 14 folds increase in p-values when we use no prior knowledge.

For demonstrating potential clinical utility, we investigated the role of epithelial-mesenchymal transition (EMT), which has established critical roles in cancer development and metastasis across multiple tumor type. The mesenchymal tumor types, low-grade gliomas and glioblastoma multiforme tend to have higher PRECISE EMT scores as compared to epithelial-type tumors such as ovarian, lung and stomach adenocarcinomas, which shows PRECISE preserves and accentuates the actual biological mechanism of these pathways in relevant tumor types. Furthermore, we also confirmed that HER2+ breast cancer samples (as determined by immunohistochemistry, IHC staining) have high PRECISE RTK scores compared to other breast samples and can potentially be used as a complementary proteomics surrogate for determining HER2- status. Our rationale for using RPPA-based proteomics data rather than mRNA’s, which have been shown to be poor surrogates for estimating protein expression^22^. Therefore, direct estimates of RTK pathway activity using proteomics data would potentially be more predictive of tumor response. Importantly, the PRECISE pathway scores, while developed in the absence of clinical data, were robustly able to predict outcomes across patient lineages.

There are several refinements and generalizations possible for the PRECISE methodology. In this article, we only focused on patient tumor samples. An interesting next step would be to investigate the functional proteome of the cancer cell lines that has recently been collected^33^. This will not only shed light on some of the fundamental cancer mechanisms but also aid drug development and screening. Moreover, integrative analysis of patient and cell-line data, and identification of commonalities and differences in network biology could reveal insights into the specific roles of tumor microenvironment in cancer development and progression. From a methodological perspective, we note that our model assumes a linear relationship between the proteins for simplicity and interpretability. It is possible to extend this approach to allow for more flexible and non-linear dependence structures^36^.

To the best of our knowledge, this is the first comprehensive exploration of pathway-specific network signatures for stratification and prediction using the cancer functional proteome. A more robust tumor stratification and pathway selection will emerge as more comprehensive set of functional pathway information from more mature proteomic data are generated across the entire proteome. Our PRECISE framework is a step towards the major goal of precision medicine — to find a disease treatment and its optimal targets while taking into account individual variability. Another contribution of this method is toward establishing the translational relevance of the functional proteome in research and clinical settings. We have also developed an R package for PRECISE and developed an online data and visualization repository, that compiles a comprehensive database of networks, scores, and pathway signatures that are easily accessible to the scientific and research community (see *availability of data and material* section below).

## Methods

### Pan-cancer multiple omics data

We used a pan-cancer reverse-phase protein array (RPPA) proteomic data set of 7,714 samples from 32 tumor types from The Cancer Genome Atlas (TCGA) (Supplementary Table S1). Using TCGA-assembler^37^, we downloaded upstream molecular profiling data, mRNA expression, microRNA expression, DNA methylation data, and clinical data from TCGA Data Coordinating Center for the matched TCGA pan-cancer samples. For mRNA expression data, RNASeqV2 data (generated by illumina GA or illumina HiSeq) were downloaded and preprocessed using ‘DownloadRNASeqData’ and ‘ProcessRNASeqData’ functions for gene-level expression. For microRNA expression data, miRNASeq data (generated by illuminaga or illuminahiseq) were downloaded using the ‘DownloadmiRNASeqData’ function. Using the ‘ProcessmiRNASeqData’ function, we processed the miRNASeq data, using the Hg19 reference genomes to map the reads. Then we annotated the microRNAs to genes using the ‘microRNA’ package in R version 3.2.0. DNA methylation data (generated by Illumina Infinium HumanMethylation 27 K or 450 K) were downloaded and preprocessed using ‘DownloadMethylationData’, ‘DownloadMethylation27Data’ and ‘DownloadMethylation450Data’.

ComBat allows users to adjust for batch effects in datasets where the batch covariate is known. In our case study, microRNA expression data were generated by either illuminaga or illuminahiseq and DNA methylation data are generated by Illumina Infinium HumanMethylation 27 K or 450 K. For cancer types that use both datatypes for microRNA, we combine the samples from illuminaga or illuminahiseq and adjust the combined data with illuminaga/illuminahiseq as batch covariate. Similarly, for DNA methylation data, we combined the datasets from Illumina Infinium Human Methylation 27 K and 450 K using the ComBat function with the known batch covariate (27 K vs. 450 K). As an illustrative example, Supplementary Fig. S23 displays PCA plots of microRNA expression and Methylation datasets for 124 READ samples. We could observe that the samples from two batches are mixed after adjusting for the batch effects for both cases of microRNA and Methylation data. Similar results were noted for other cancer types. The pathways and the members used in our preliminary data (Supplementary Table S2) are selected based on recent literatures on RPPA for various cancer types^22,32,33^.

### PRECISE framework

We developed a general Bayesian framework to estimate cancer-specific and patient-specific networks and elicit patient-level pathway scores by integrating data arising from multiple platforms and incorporating existing network information. With Pan-cancer TCGA proteomic, genomic and epigenomic data, PRECISE uses a multi-scale approach (Fig. 1). The implementation of PRECISE on proteomic networks only requires two inputs: (1) data — proteomic data and other (upstream) molecular profiles (e.g., gene expression, microRNA expression, DNA methylation) and (2) prior information — known pathway annotations and PPIs. Briefly, in the first step of PRECISE, a protein-protein causal network is estimated and combined with the prior information (from PPI’s). Other upstream molecular data are then integrated in three sequential steps to produce cancer-specific, patient-specific networks and pathway scores (PRECISE scores), which are subsequently used for tumor subtype classification and clinical outcome prediction.

Briefly, we use a Bayesian regression model, in which each protein is regressed on covariates that include the protein regulators learned from the data-driven causal network and the PPIs (as prior information), and features from upstream data. The selected regulators for all proteins constitute cancer-specific integrated network. Given the cancer-type-specific network, we deconvolve the patient-specific PRECISE scores, using the status of each protein (node) of the networks as suppressed, neutral, or activated. We use these PRECISE scores for patient stratification and clinical outcome prediction. PRECISE is a general framework to achieve patient-level and pathway-level signatures by sequential estimations of cancer-specific (global) and patient-specific (local) networks. The novelty of our methodology lies in conflating causal structure learning, Bayesian graphical models and variable selection at both population (cancer-specific) and patient-pathway levels. The integrative modeling strategy involves smooth calibration of the prior by applying a causal structure learning method on resampled data, defining a patient-specific network using cancer-specific network with patient-specific labels on the nodes (proteins) with the estimated activation statuses of proteins, and subsequently estimating patient-specific pathway and network scores.

#### Step 1: Bayesian estimation of integrated cancer-specific networks

We aimed to estimate integrated cancer-specific networks using Bayesian regression methods on each of the proteins with other molecular profiling data. For regulatory relationship among proteins, let ${w}_{ij}$ be the weight from protein *i* to protein *j*, where protein *i* is a regulator of protein *j* or protein *j* is a target of protein *i*. We decide the weights ${w}_{ij}$ by calibrating *prior inclusion probabilities* for the protein regulators based on data-driven causal structure and known PPI scores (discussed in the next section). Because both ${w}_{ij}$ and ${w}_{ji}$ can be nonzero, the directional relation can be uncertain and the strength in the weight reflects the confidence of such relations. Suppose ${{\rm{y}}}_{{\rm{i}}}$ is the $n\times 1$ vector that includes the expression values of protein *i* for *n* patients and includes K_i_ covariate vectors ($n\times 1$) for *n* patients, gathered from upstream platforms, mRNA expression (modulated by DNA methylation or independent of DNA methylation) and microRNA expression. For protein *i*, the $n\times 1$ expression vector ${{\rm{y}}}_{{\rm{i}}}$ (centered with its mean) is modeled as^13^

$${{\rm{y}}}_{{\rm{i}}}={\sum }_{{\rm{j}}\in {\rm{upa}}({\rm{i}})}{{\rm{\beta }}}_{{\rm{ij}}}^{(p)}{y}_{{\rm{j}}}+\,{\sum }_{{\rm{k}}=1}^{{{\rm{K}}}_{{\rm{i}}}}{{\rm{\beta }}}_{{\rm{ik}}}^{(c)}\,{x}_{{\rm{ik}}}+{\epsilon }_{{\rm{i}}}={Z}_{{\rm{i}}}{\beta }_{{\rm{i}}}+{\epsilon }_{{\rm{i}}},$$

where $${\epsilon }_{{\rm{i}}} \sim N(0,{\sigma }_{i}^{2})$$ and $$upa(i)$$ is the set of possible protein regulators of protein $$i$$ such that $$\{t:{w}_{ti} > 0\}.$$$$\{{\beta }_{ij}^{(p)}:j=1,\ldots ,\,|upa(i)|\}$$ are the regression coefficients for the protein regulators, $$\{{\beta }_{ij}^{(c)}:\,j=1,{K}_{i}\}$$ are the coefficients for other covariates, and $${\beta }_{i}$$ is the coefficient vector including all the coefficients. We employ Zellner’s g-prior on $${\beta }_{i}$$:

$${\beta }_{i}|g \sim N(0,{{\rm{\sigma }}}_{{\rm{i}}}^{2}{(\frac{1}{g}{Z}_{{\rm{i}}}^{T}{Z}_{{\rm{i}}})}^{-1}).$$

The hyper-parameter *g* reflects the prior on ${\beta }_{i}=0$. A higher value of *g* implies deviation from ${\beta }_{i}=0$, and we assigned $g=n,\,$which is the unit information prior on Zellner’s g. We also set the prior $p({\sigma }_{i})\propto {\sigma }_{i}^{-1}$. Then we performed full enumeration using the Markov chain Monte Carlo algorithm. After performing all node-wise regressions following the above model, we select the median probability model^38^ to infer the posterior network. Specifically, for each regression of protein *I*, its integrated cancer-specific regulators that have edges directed toward the protein *i* are defined by the proteins or other covariates that have a posterior inclusion probability (the sum of the posterior model probabilities for all models, where a covariate was included) greater than 0.5. Therefore, among proteins, the network includes both directed *regulatory* edges - when regulator proteins of a protein are not targets of the protein and *correlative* edges- where-in both proteins in a link are regulators and targets.

#### Step 2: Constructing PRECISE (patient-specific) networks

A PRECISE network is the integrated cancer-specific network with patient-specific labels on the nodes (proteins). Specifically, the activation statuses of the nodes are evaluated by estimating the posterior predictive density of each of the proteins for each patient. To determine the activation status of a protein *i* for a patient *j* (${y}_{ij}$), we computed the posterior probabilities of the protein to lie in the $\delta$-interval around zero (${p}_{ij}^{0}$), to be greater than $\delta$ (${p}_{ij}^{+}$), or less than $-\delta$ (${p}_{ij}^{-}$). Then, we decided whether a protein is neutral, activated, or suppressed, depending on the maximum of the three posterior probabilities, $max\{{p}_{ij}^{0},{p}_{ij}^{+},\,{p}_{ij}^{-}\}$. Thus, patients with the same tumor type have different node labels, suppressed, neutral or activated while the structure of the networks is the same. Using $\delta =0.5$, we calculated PRECISE networks across all patients with 32 tumor types.

#### Step 3: Calibrating PRECISE (patient-specific) scores

To compute pathway activity scores for each patient, we derive summary measures from the PRECISE networks obtained from the Step 2, indicating that the entire pathway is suppressed, neutral and activated. Under the PRECISE networks, the number of nodes that have edges from protein $i$, $|\{j|i\to j\,or\,i\leftrightarrow j\}|$ is denoted by ${C}_{i}$. For a given pathway with *p* genes, the pathway activity scores for a patient *j* is given by

$${\kappa }_{j}^{+}=\frac{1}{p}{\sum }_{i=1}^{p}{p}_{ij}^{+}({C}_{i}+1),{\kappa }_{j}^{-}=\frac{1}{p}{\sum }_{i=1}^{p}{p}_{ij}^{-}({C}_{i}+1),\,{\rm{and}}\,{\kappa }_{j}^{0}=\frac{1}{p}{\sum }_{i=1}^{p}{p}_{ij}^{0}({C}_{i}+1),$$

for activated, suppressed, and neutral PRECISE scores. Note that these patient-specific pathway scores are weighted averages of the posterior probabilities for suppressed $({p}_{ij}^{-})$, neutral $({p}_{ij}^{0})$, and activated $({p}_{ij}^{+})$ statuses of proteins by the number of target or correlative proteins. Therefore, hub proteins in the pathway, that exercise more control over the network through higher target or correlative proteins are given higher weights towards determining the cumulative network score. For a given pathway and each patient, the *PRECISE pathway status*- which indicates that the pathway is suppressed, neutral, or activated for the patient, can be decided by maximum of the three PRECISE pathway scores, ${\rm{\max }}\,\{{\kappa }_{j}^{+},{\kappa }_{j}^{-},\,{\kappa }_{j}^{0}\}$ for each patient *j*.

#### Prior calibration and causal structure learning

To decide the prior inclusion probabilities for protein regulators (${w}_{ij}$), we estimate the cancer-specific protein causal network that determines the protein regulators by the directions of the edges and the contribution to the target proteins by the weights of the edges. We use this weighted causal structure to determine the priors for the regression in combination with the known PPIs. The edge-wise average values of the weights in the data-driven causal structure and the PPI scores were used for the prior inclusion probabilities (${w}_{ij}$) for the protein regulators.

Using RPPA data in each cancer type, we constructed the causal structure of proteins using the PC algorithm^39^ which relies on conditional independence tests with a certain significance level $\alpha .$ The causal structure is represented by a graph with directed (→ or ←) or bi-directed (↔) edges. A bi-directed edge means that the direction is not identifiable from the data. In this analysis, we chose a relatively large value for the tuning parameter $\alpha =0.01$, so that the resulting graph would include a large number of edges. Then we measured the weights of the edges by investigating the stability under a subsampling procedure. From the stability selection^40^, the stability is defined by the relative frequency of the occurrence of edges under the sub-sampling scheme.

The causal structure for proteins is estimated as follows:

- Step 1.1. Given $\alpha =0.1$, fit the PC algorithm and denote the estimated edge set by $\hat{E}$.
- Step 1.2. Draw a random sub-sample (denoted by *I*) of $\{1,\ldots ,n\}$ of size $\lfloor n/2\rfloor$ without replacement, where $\lfloor \,\cdot \,\rfloor$ is the greatest integer function.
- Step 1.3. Fit the PC algorithm for $\alpha$ values 100 grid points in $(0,0.1)$ on the sub-sample *I*. After iterating step 1.2 through step 1.3 100 times, each edge in $\hat{E}$ has $100\times 100$ different directions across all $\alpha$ values and sub-samples *I*. The *selection probability* for an edge in $\hat{E}$ is calculated as the likelihood of it being in the edge sets with respect to the random sub-sampling. In the results, each edge in $\hat{E}$ has 100 selection probabilities for all $\alpha$ values, and the maximum values of the selection probabilities across different $\alpha$ values are used for the edge weights for the causal structure. Those maximum selection probabilities over the different tuning parameters are used for variable selection and graphical models, enabling the control of the rate of false positive findings^40–42^.

In combination with the data-driven causal structure, we collected the PPIs from the STRING database (http://string-db.org), which provides known and predicted PPIs. Each interaction is associated with a combined confidence score that integrates the various types of evidence such as genomic context, high-throughput experiments, co-expression, and previous knowledge. STRING v10 data were downloaded. As the weighted prior network, we collected all PPIs in the database, where each PPI has a score ranging from 0 to 1. A higher score means higher confidence. Note that the PPI network from STRING database only serves as a prior since the PPI scores in the STRING DB, ranged from 0 to 1 are used in combination with the *de novo* cancer-specific PPI scores, also ranged from 0 to 1 from the causal structural learning described above in each type of cancers.

Our PRECISE estimation method for cancer-specific networks conflates Bayesian variable selection and causal structure learning. Thus, if the data does not support a particular a priori network structure, it will exhibit low posterior probabilities in the final estimands and will thus be naturally filtered out. Instead of performing hard-thresholding (e.g. PARADIGM^16^) on the known network, we use the soft-thresholding approach by calibrating the priors of regressions based on the average interaction scores from data-driven *de novo* cancer-specific causal structure and existing global PPI information. Finally, our methodology is general enough to be combined with any PPI database that is relevant to scientific context.

### Clustering analysis using PRECISE scores

The PRECISE method provides processed and highly interpretable pathway-level functional summaries that compress the regulatory relations from DNA methylation, mRNA/microRNA expression, and protein regulators. Using the resulting PRECISE score matrix as an input data, we obtain robust pan-cancer stratification. For a pathway with *p* genes and a patient *j*, we combined the activated and suppressed PRECISE scores, ${\kappa }_{j}^{+}+{\kappa }_{j}^{-}=\frac{1}{p}{\sum }_{i=1}^{p}({p}_{ij}^{+}+{p}_{ij}^{-})({C}_{i}+1)$. After computing the activated and suppressed PRECISE scores for all patients and all 12 pathways, we constructed a 6844 × 12 data matrix (after matching samples across platforms), with each row corresponding to a patient and each column corresponding to a pathway. Based on the Euclidian distance of the score matrix, we applied hierarchical clustering using Ward’s method. To determine the number of clusters, we used the gap statistic^43^.

## Electronic supplementary material

Supplementary Information

## Data Availability

PRECISE R package is available on https://github.com/MinJinHa/PRECISE. We also have created an online repository, https://mjha.shinyapps.io/PRECISE, that compiles a comprehensive database of networks, scores, and pathway signatures and includes PRECISE R package and all the pan-cancer input data made available in a format that can be directly used in the PRECISE R package, and pan-cancer survival data used for prognostic validation.
