# Supplementary material for: Personalized Integrated Network Modeling of the Cancer Proteome Atlas
Source: Sci Rep. 2018 Oct 8;8:14924. doi: 10.1038/s41598-018-32682-x (PMC6175854; doi:10.1038/s41598-018-32682-x)
Supplement: Supplementary file 1 — Supplementary Information [file 41598_2018_32682_MOESM1_ESM.pdf]

SUPPLEMENTARY INFORMATION FOR

Personalized Integrated Network Modeling of The Cancer Proteome Atlas

Min Jin Ha[1], Sayantan Banerjee[2], Rehan Akbani[3], Han Liang[3], Gordon B. Mills[4], Kim-Anh Do [1], and Veerabhadran Baladandayuthapani[1,*]

[1] Department of Biostatistics, The University of Texas MD Anderson Cancer Center, Houston, TX 77030, USA

[2] Department of Operations Management and Quantitative Techniques at the Indian Institute of Management, Indore, India

[3] Department of Bioinformatics and Computational Biology, The University of Texas MD Anderson Cancer Center, Houston, TX 77030, USA

[4] Department of Systems Biology, The University of Texas MD Anderson Cancer Center, Houston, TX 77030, USA

Section S1. Supplementary Note

Section S1.1 External validation

We performed external validation that clearly demonstrate the superior performance of our PRECISE algorithm as compared to other established and current approaches for obtaining patient-specific pathway scores across multiple tumor types. For objective external validation of the PRECISE algorithm, we compared PRECISE scores with two established and previously published scores: a proteomics-based pathway score obtained using the same RPPA data reported by Akbani et al (Akbani et al., 2014) -- which we call “native” score and the PARADIGM (Vaske et al., 2010) -- which measures the patient-specific genetic activities incorporating curated pathway interactions among gene using expression data. We compared their prognostic power and ability to detect tissue-specific signals (as detailed below).

External methods (Methodological comparison)

The ***Naïve pathway score*** was defined as the cumulative sum of all protein expression in a particular pathway. This method gives all proteins equal weights in estimating the pathway-level score, thus ignore existing and de novo network information.

The ***Native pathway score*** was defined by the sum of all positive regulatory components minus that of the negative regulatory components in a particular pathway as reported by Akbani et al, 2014 and also has been used in a recently published TCGA paper for uterine carcinosarcoma (UCS), Cherniack et al., 2017. This method takes prior directionality (+/-) into account to yield pathway scores while de novo networks are not used.

PARADIGM uses pathways from the National Cancer Institute Pathway Interaction Database (NCI-PID). They refer to molecular entities as “concepts”, which include gene products such as proteins and miRNAs, small molecules, protein complexes, and abstract concepts. Each concept is represented as “node” in the PARADIGM graphical model. The PARADIGM algorithm assigns an integrated pathway level (IPL) reflecting the activity of a concept. The significance of the IPL for each concept in each patient sample is assessed using a permutation analysis. We used the PARADIGM analysis (RNAseq and Copy number) results from http://firebrowse.org. [1] This method uses known pathway information but no de novo networks.

The major difference from a methodological perspective between these methods and our PRECISE algorithm is that we estimate and construct de novo cancer-specific networks to calibrate the patient-level pathway signatures by exploiting multiple sources of information obtained from cancer-specific multi-platform data, causal structure leaning and existing interaction database. It is well established that sub-networks within signaling pathways and their products undergo changes in response to different conditions. The Naïve, Native and PARADIGM methods do not use the cancer-specific de novo networks.

Such *network rewiring* in cancers are manifested at the level of signaling networks and is not currently well understood (Lee et al., 2012, Creixell, et al., 2015). Thus, assessing the cancer-specific topology and structure of proteomic signaling networks as well as relating them to (prognostic) clinical outcomes are important task toward understanding the biological mechanisms behind cancer development and progression and importantly, therapeutic implementation.

Power to detect tumor-specific signal

We compared the PRECISE scores with the Native and PARADIGM scores in the same context of the recently published paper (Cherniack et al, 2017; Cancer Cell) across four tumor types: Uterine corpus endometrial carcinoma (UCEC, n=439), ovarian serous cyst adenocarcinoma (OV, n=431), Sarcoma (SARC, n=224) and uterine carcinosarcoma (UCS, n=48) – to assess shared/differentiated pathway-based features. For PARADIGM analysis, we selected 985 concepts in 18 significant pathways that are a union set of significant pathways using cutoff 0.15 of the significance ratio (avg. Num. Perturbations/Cohort size) for each tumor type (Vaske et al., 2010). Our native scores were computed as described in the main article and do not explicitly include network information.

The clustered heatmaps for the native, PRECISE and PARADIGM scores are shown in Supplementary Figure S17. There is clear evidence that our PRECISE method preserves tumor-specific signals (94% of UCS, 99% of UCEC, 99% of SARC and 98% of OV samples were separately clustered) better than native and PARADIGM scores. In particular, the clusters assessed by both native and PARADIGM contained samples with mixed tumor types. To further investigate how much the specific pathway scores vary across cancer types, we plot the boxplots of combined (activated and suppressed) scores from the PRECISE for all the 12 pathways in Supplementary Figure S19. In comparison to the boxplots with Figure 7 B in Cherniack et al., 2017, PRECISE produced much lower p-values across pathways and showed more evident pattern across different tumor types (Supplementary Figure S18). UCS samples showed much lower activity/suppressed levels in Apoptosis, Breast reactive, cell cycle, RAS/MAPK, TSC/mTOR pathways than UCEC, OV, and SARC. UCS and SARC samples showed similar patterns in core reactive and hormone receptor pathways. Collectively, this suggests PRECISE has much higher prognostic power in detecting pathway-specific signals across tumor types.

Prognostic power

There were 449 matched KIRC samples in the PARADIGM score matrix from 454 patients in our RPPA dataset. Using the cutoffs of -0.25 and 0.25 (as used in Vaske, et al. 2010) of the IPL (Integrated pathway level) scores, we classified the patient-level pathway scores into suppressed, neutral and activated. The apoptosis was measured as nodes (concepts) within 10 different PARADIGM pathways. From the Cox-proportional hazards model, all the p-values for the 10 PARADIGM apoptosis concepts were greater than 0.08. For the Paradigm pathway with the lowest p-value, the Kaplan-Meier curves are shown in Supplementary Figure S17. The PRECISE method performs better than PARADIGM and native methods to predict patients' survival times in unsupervised manner. We also compared the native method and PRECISE method across all 31 cancer types and 12 pathways. The PRECISE scores were significantly associated with patients' survival times for the 30 tests with FDR at 0.1, while the native score provided only 5 significant associations (Figure 4 c).

Next, we compared the survival outcome association of our protein-based pathway signatures of the 12 pathways to that of 1000 random protein signatures. For a given pathway, we obtain a random signature as follows: (1) randomly select proteins of identical size to the number of proteins in the pathway; (2) compute the first principal component (PC1) using the selected proteins and (3) split the patient cohort into three groups according to 30 and 70 percentiles of the PC1. Venet et. al., 2011 suggested the PC1 method because it yields more stable stratification than using K-means or hierarchical clustering. We use the proportions of random signatures that work better than those obtained from PRECISE as another measure of the significance with a False-discovery rate (FDR) adjusted p-values. At the cutoffs of proportions, 0.1 and 0.05, we found 39 and 23 pathway-based signatures respectively across all 31 tumor types. While using FDR=0.1, we found 31 discoveries (Figure 5); 15 discoveries met both of the criteria. Using this random signature approach, there

were additional new findings: for example, signatures of Core reactive, TSC/mTOR and Hormone receptor pathways were selected for a rare cancer, Lymphoid Neoplasm Diffuse Large B-cell Lymphoma (DLBC) by using the criteria, that were not found by our previous FDR-adjustment (see Supplementary Figure S21 for boxplots of the p-values of associations of 1000 random signatures). Because the two approaches provide distinct information on the prognostic power, we proved an additional table for the union set of findings using FDR and random signature approaches (Supplementary Table S9).

Section S1.2 Pan-cancer multiple `omics data and Preprocessing

Using TCGA-assembler [1], we downloaded mRNA expression, microRNA expression, DNA methylation data, and clinical data from TCGA Data Coordinating Center. For mRNA expression data, RNASeqV2 data (generated by illuminaga or iluminahiseq) were downloaded and preprocessed using `DownloadRNASeqData' and `ProcessRNASeqData' functions for gene-level expression. For microRNA expression data, miRNASeq data (generated by illuminaga or illuminahiseq) were downloaded using the `DownloadmiRNASeqData' function. Using the `ProcessmiRNASeqData' function, we processed the miRNASeq data, using the Hg19 reference genomes to map the reads. Then we annotated the microRNAs to genes using the `microRNA' package in R version 3.2.0. DNA methylation data (generated by Illumina Infinium HumanMethylation 27K or 450K) were downloaded and preprocessed using `DownloadMethylationData', `DownloadMethylation27Data' and `DownloadMethylation450Data'. For cancer types that had both types of assays, the overlapping features between both assay types were used. Then we applied ComBat [2] to adjust for the known batch effects using an empirical Bayes framework.

We assumed that DNA methylation affects protein expression levels by influencing mRNA expression. For a gene, we selected a CpG site by computing the absolute correlations between the mRNA expression and CpG sites outside the `gene body' region. We decomposed the expression changes (G) of each gene into two components, which are explained by methylation (M) and mechanisms other than methylation:

$$G = G^M + G^{\widetilde{M}}, G^M = \beta M,$$

where β is a regression coefficient that represents the `gene methylation' effect. A CpG site corresponding to a gene is chosen by regression on the combined data, including data from 6,844 patients across all 32 lineages.

Section S1.3 Enrichment analysis of clusters

To investigate our PRECISE clusters in relation to clinical outcomes and mutation data, we defined an *enrichment probability (EP)*. For demonstration purposes, we take as example of breast cancer analysis. The same procedure can be applied for the enrichment analysis of mutation data. We define an EP for each subtype of breast cancer and each of the clusters. We have breast cancer subtypes of PAM50, basal-like, HER2-enriched, luminal A, and luminal B [3]. The BRCA patients were located in three clusters, C1, C2 and C3. For each subtype, we defined a 3-dimensional vector $\boldsymbol{P} = (p_1, p_2, p_3)^{\boldsymbol{T}}$, where p_i is the enrichment probability for cluster i. This probability vector indicates whether the patients in the subtypes are grouped together. For each subtype, we followed a Bayesian hypothesis testing framework to test whether the proportion of patients from the subtype in cluster i is significantly higher than the proportion of patients from outside the subtype in cluster i. Then, we considered the following two-way table with the three clusters and a subtype:

	C1	C2	C3	Total
Subtype (-)	n_{01}	n_{02}	n_{03}	N_0
Subtype	n_{11}	n_{12}	n_{13}	N_1

We denote the number of patients from outside the subtype in cluster i by $n_{0i}, i = 1,2,3$ and the number of patients from the subtype in cluster i by $n_{1i}, i = 1,2,3$. The total numbers of patients from the subtype and from outside the subtype are denoted by N_0 and N_1, respectively. We assume our model by

$(n_{j1}, n_{j2}, n_{j3}) \sim Multino\square ial(N_j, \theta_{j1}, \theta_{j2}, \theta_{j3})$ and $(\theta_{j1}, \theta_{j2}, \theta_{j3}) \sim Dirichlet(1,1,1)$.

The posterior distribution of $(\theta_{j1}, \theta_{j2}, \theta_{j3})$ is $Dirichlet(n_{j1} + 1, n_{j2} + 1, n_{j3} + 1)$. For each cluster i, we calculate the posterior probability that $\theta_{0i} < \theta_{1i}$,

$$p_i = \Pr(\theta_{0i} < \theta_{1i} | Data),$$

which can be computed using Monte Carlo methods.

Section S1.4 Calculation of concordance score

For each cancer type, we divided the whole data set into two parts, a training data set and a test data set. Using the test data, we predicted the protein expression value for each node under the model evaluated from the training data. We computed the concordance score as follows: for each cancer and each pathway,

$$\frac{1}{n_{test}} \frac{1}{p} \sum_{j=1}^{p} \sum_{i}^{n_{test}} I\left(y_{ij} \in \left(\hat{y}_{ij} - 2 \times se(\hat{y}_{ij}), \hat{y}_{ij} + 2 \times se(\hat{y}_{ij})\right)\right),$$

where n_{test} is the number of samples in the test data, p is the number of proteins (nodes) in the pathway, and y_{ij} is the observed value for the ith test sample and j^{th} protein. We based our analysis on 10-fold cross-validation.

Section S1.5 Calculation of permutation p-values for network connectivity scores

We investigated on the relationship between sample sizes and the connectivity score (CS), which was the constructed as the ratio of the observed number of edges in the network to the total number of possible edges (see Supplementary Figure S24 (left panel)). We did find a marginal positive correlation between sample size and CS with Spearman correlation, 0.55 -- which indicates that CS values are (somewhat) driven by the sample sizes, although there is some variability. This is not surprising given the fact that we have more statistical power to detect true network edges as sample size increases.

To investigate this further, we generated the null CS distribution for a given pathway and tumor type as follows. Briefly, for each tumor type and pathway, we randomly select the same number of proteins in a given pathway while the covariates from upstream platforms (mRNA, microRNA and Methylation) are matched to the this randomly selected set of proteins. After constructing networks from 1000 random permutations of the proteins, a null distribution of CS is obtained for the pathway and cancer type. For the hypothesis, that a pathway shows high level of cross-signaling than expected at random, we compute the permutation p-value, $p_{cs} = \sum_{i=1}^{1000} I(\widetilde{CS_\iota} > CS)/1000$, where $I(\cdot)$ is an indicator function, $\widetilde{CS_\iota}$ is the CS value obtained from ith permutation and CS is the CS value from the data. These p_{cs} values were not correlated with sample size (Supplementary Figure S24, right panel) and thus provide an inherent sample size adjustment.

Section S1.6 Robustness of cancer-specific integrative networks

We have validated the robustness of our cancer-specific integrative networks by comparing with a recent method OncoPPI (Li et al., 2017) and a resampling method. The OncoPPI network (Li et al., 2017) expands the lung-cancer associated protein interaction landscape for discovery of novel cancer targets and connects tumor suppressors to available drugs, offering an experimental resource for exploitation of PPI-mediated cancer vulnerabilities. In their analysis, 83 genes were selected based on frequency of alterations in lung cancer and known involvement in cancer signaling pathways and only 12 genes were overlapped with the genes used for

our analysis. We applied OncoPPI (http://oncoppi.emory.edu) to the 12 overlapping genes (see Supplementary Figure S25 for OncoPPI sub-network for the 12 genes). The PRECISE networks for LUAD and LUSC are displayed in Supplementary Figure S26 and showed the same edge structure between the two tumor types, except for the directed and correlative edges between AKT1, AKT2, AKT3 and PTEN in LUAD and LUSC samples, respectively. The OncoPPI network had two edges AKT1-RAF1 and AKT1-MAPK14 that were not included in the PRECISE network (PRECISE network had a correlative edge between RAF1 and MAPK14). We could conclude that the two approaches provide distinct networks and highlight several differences in the methodologies:

(1) We estimate PPI network from observational data integrating upstream data such as mRNA expression, microRNA expression and DNA methylation. Thus, the PPI network we obtain is a conditional network after adjusting for regulators from upstream data as well as protein regulators in a pathway. On the other hand, OncoPPI is based on a PPI detection platform that identifies marginal direct interactions.

(2) Our approach uses pathway-based procedure with the two main reasons: (1) for conditional networks (see item (1)), adjusting for only the regulators within pathways is an efficient strategy to use prior knowledge; and (2) we have a final aim of measuring individual-specific pathway activities and finding pathway-based prognostic models. Note that OncoPPI selected a set of 83 genes based on frequency of alterations in lung cancer and known involvement in cancer signaling pathways, and obtained marginal interaction data for each pair of the proteins.

(3) OncoPPI uses the previously reported PPIs by hard-thresholding, edge inclusion/exclusion (Figure 1c of Li et al., 2017). Our PRECISE estimation method for cancer-specific networks conflates Bayesian variable selection and causal structure learning. Thus, if the data does not support a particular a priori network structure, it will exhibit low posterior probabilities in the final estimands and will thus be naturally filtered out. In addition, instead of performing hard-thresholding (e.g. OncoPPI) on known PPIs, we use soft-thresholding approach by calibrating the priors of regressions based on the combined interaction scores from data-driven de novo causal structure and existing PPI information.

For assessing robustness of our estimated protein network, we selected TSC/mTOR pathway for LUAD patients that had a significant connectivity score (CS), 0.8 with the permutation p-value, 0.044 with 7 correlative edges and one directed edge. To make a sub-sample set, 285 samples (80%) are randomly sampled from the 356 LUAD samples after matching RPPA, mRNA expression, miRNA expression and DNA methylation data. For each sub-sample set, we built LUAD-specific TSC/mTOR pathway using our PRECISE algorithm. We investigate the stability of an edge in the LUAD-specific TSC/mTOR network across 100 sub-samples by computing the proportion of the sub-sample sets that produced the network with the edge (Supplementary Table S9). 6 out of 8 edges were kept across all 100 sub-sample sets. For the correlative edge, RB1-RPS6KB1 was conserved across 48 sets, 23 had the directed edge, RB1-> RPS6KB1 (23 sets selected RB1 as a covariate of RPS6KB1 and did not select RPS6KB1 as a covariate of RB1 in the Bayesian regressions), and other 29 sets selected no edges between RB1 and RPS6KB1. The directed edge RPS6->RB1 is conserved for 88 sub-sample sets.

Section S1.7 Normalized mutual information

To compare the clustering performance, we computed the normalized mutual information (NMI) scores to evaluate the dependence between clusters and tumor types for n samples. Given two partitions, $A = \{A_1, \ldots, A_R\}$ and $B = \{B_1, \ldots, B_R\}$, the contingency table that represents the overlap between A and B is denoted by $C = \left(C_{ij}\right)_{R\times S}$

, where C_{ij} is the number of samples that the clusters A_i and B_j share. The NMI score between A and B are defined as follows:

$$\mathrm{NMI(A,B)} = \frac{-2\sum_{i=1}^{R}\sum_{j=1}^{S} C_{ij}\log\left(\frac{C_{ij}n}{C_{i\cdot}C_{\cdot j}}\right)}{\sum_{i=1}^{R} C_{i\cdot}\log\left(\frac{C_{i\cdot}}{n}\right) + \sum_{j=1}^{S} C_{\cdot j}\log\left(\frac{C_{\cdot j}}{n}\right)}.$$

NMI is a quantitative measure of the overlap between two clusters, taking values from 0 to 1 -- with values close to 0, when the two clusters are totally dissimilar, and 1 where they are identical.

References

1. Zhu, Y., Qiu, P. & Ji, Y. TCGA-assembler: open-source software for retrieving and processing TCGA data. *Nature methods* **11**, 599-600 (2014).
2. Johnson, W.E., Li, C. & Rabinovic, A. Adjusting batch effects in microarray expression data using empirical Bayes methods. *Biostatistics* **8**, 118-127 (2007).
3. Cancer Genome Atlas, N. Comprehensive molecular portraits of human breast tumours. *Nature* **490**, 61-70 (2012).

Section S2. Supplementary Figures and Tables

A

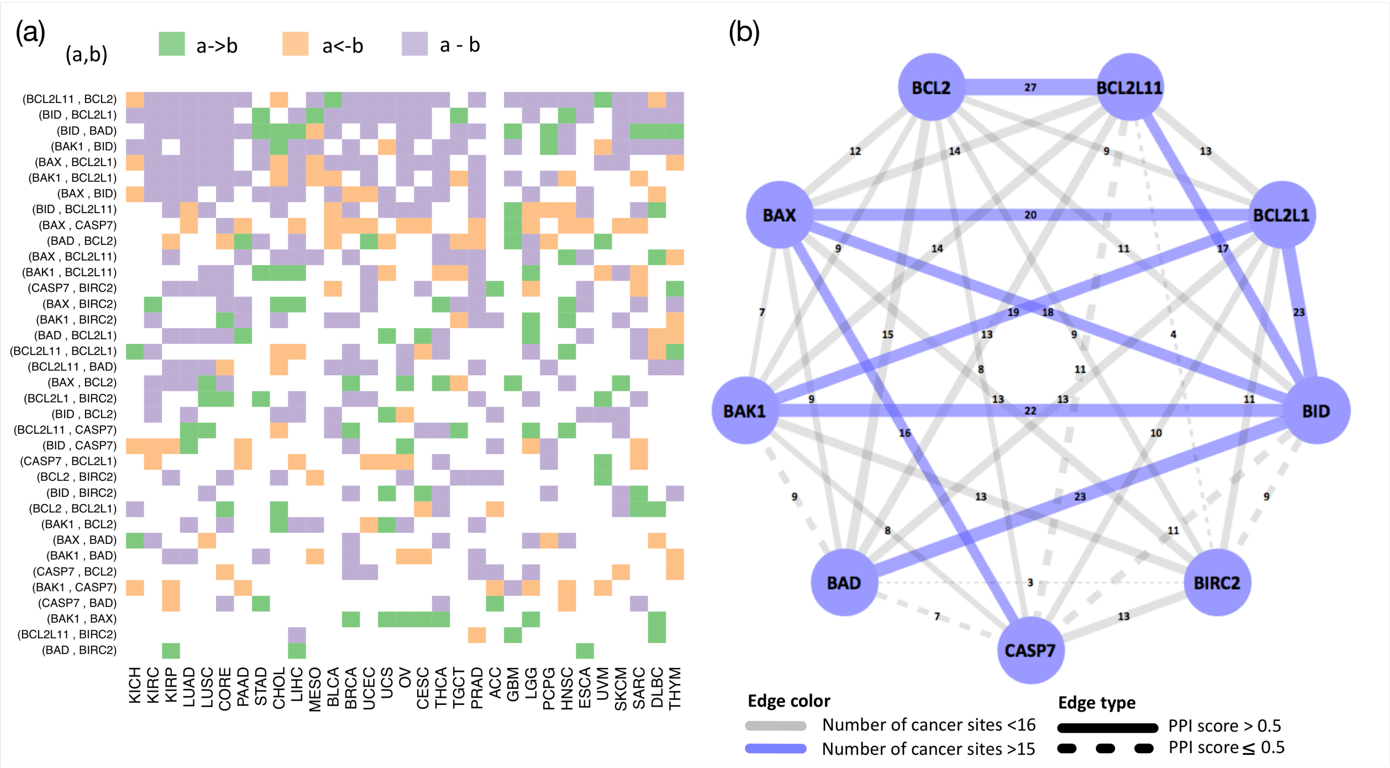

B

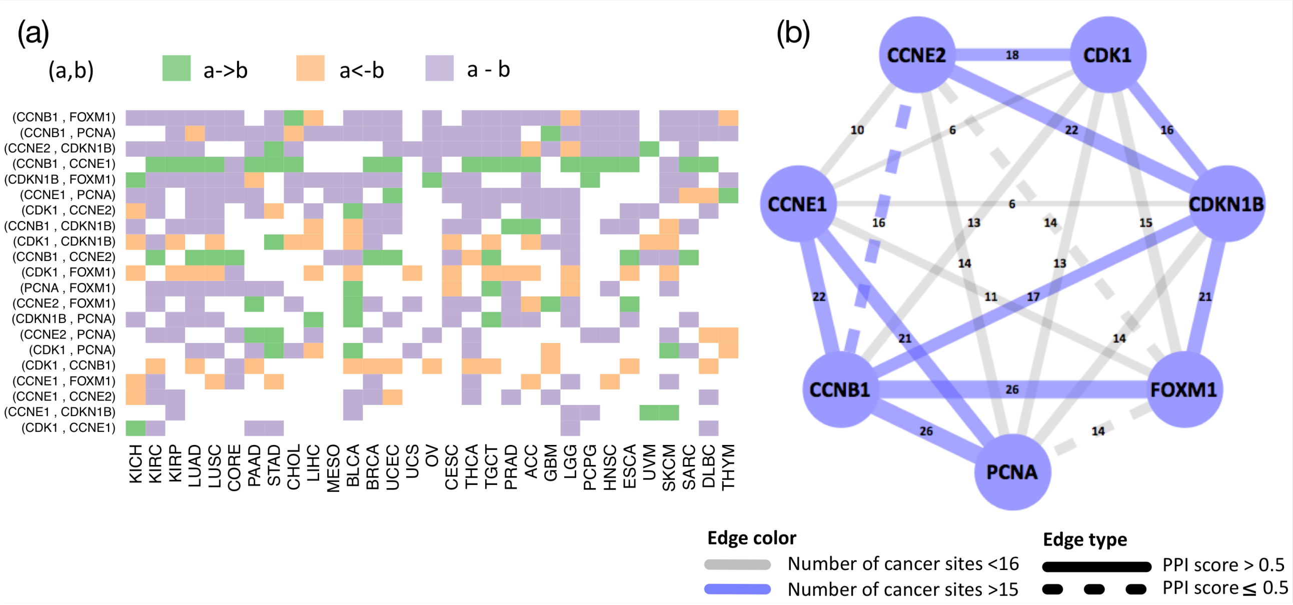

[Supplementary Figure S1] Cancer-specific networks for apoptosis (A) and cell cycle (B) pathways. (a) Heatmap depicting regulatory (-> or <-) and correlative (-) edges across all tumor lineages. (b) Network, where each of the edges is weighted and labeled by edge consistency (EC), defined as the number of tumor types that hold the particular edge.

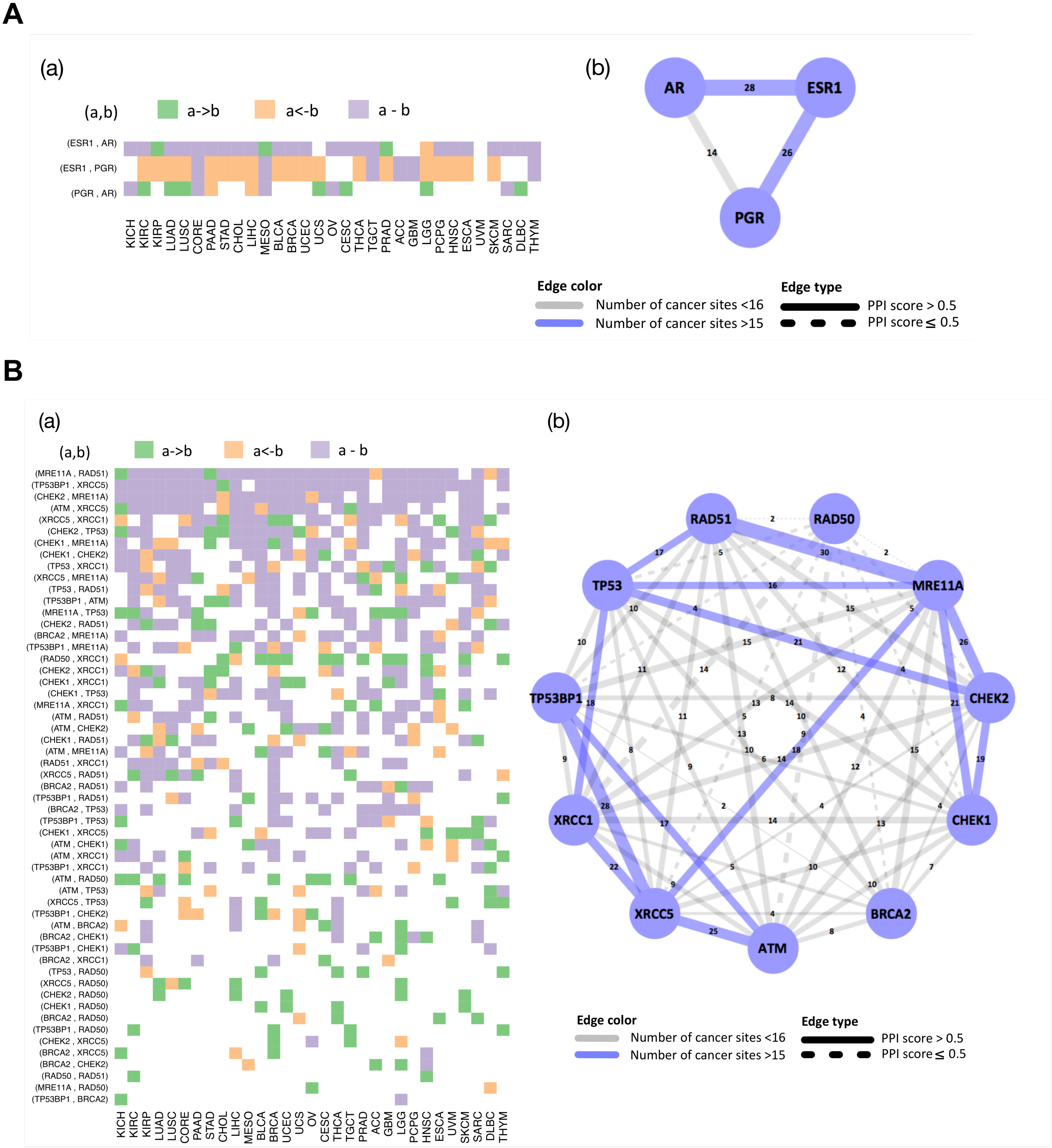

[Supplementary Figure S2] Cancer-specific networks for hormone receptor (A) and DNA damage response (B) pathways. (a) Heatmap depicting regulatory (-> or <-) and correlative (-) edges across all tumor lineages. (b) Network, where each of the edges is weighted and labeled by edge consistency (EC), defined as the number of tumor types that hold the particular edge.

A

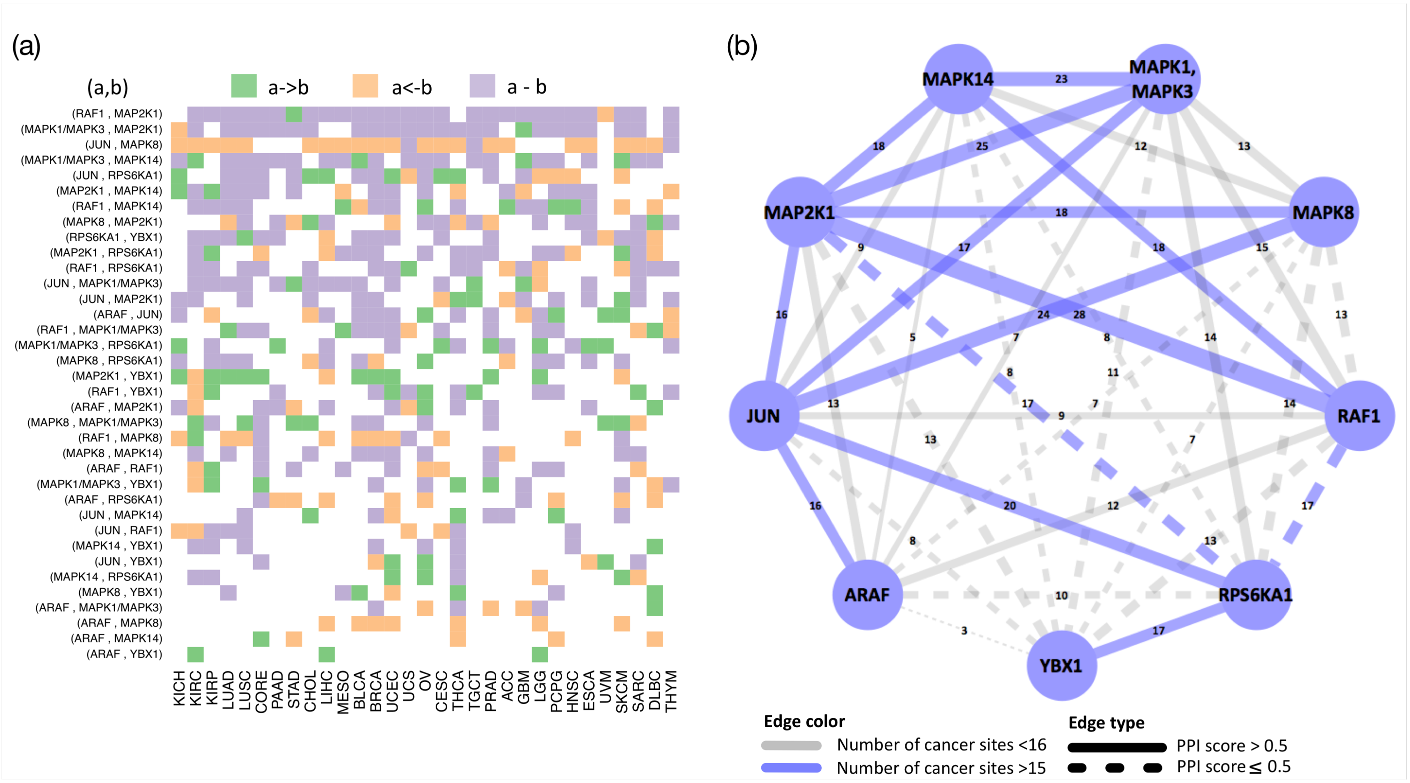

B

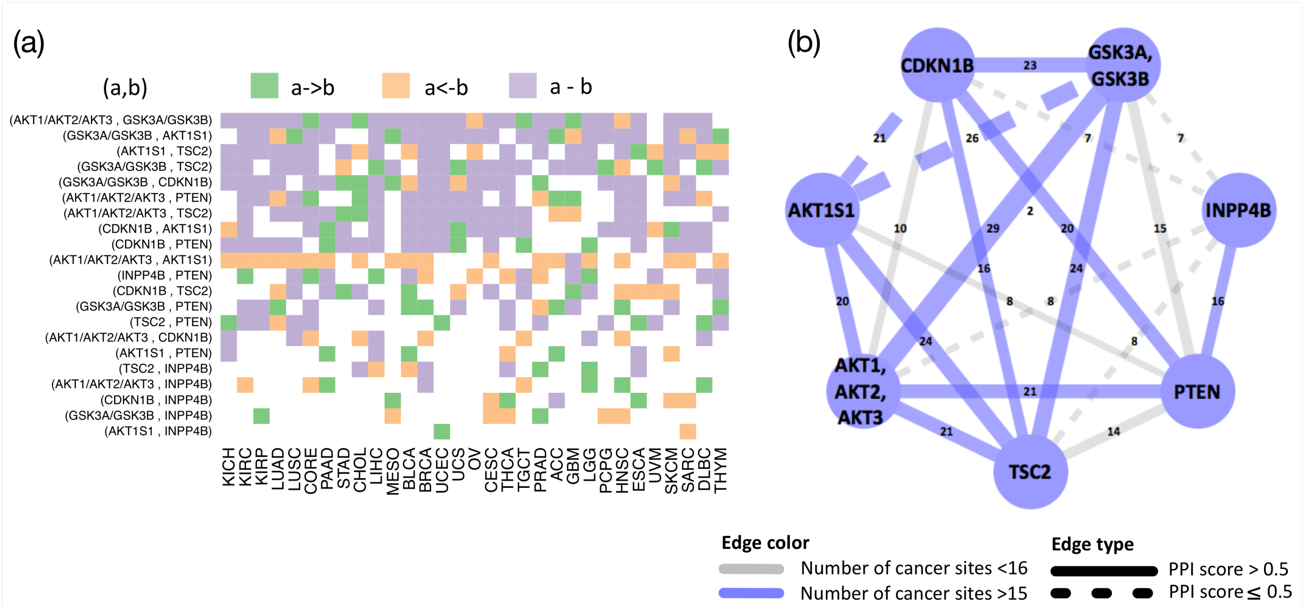

[Supplementary Figure S3] Cancer-specific networks for RAS/MAPK (A) and PI3K/AKT (B) pathways. (a) Heatmap depicting regulatory (-> or <-) and correlative (-) edges across all tumor lineages. (b) Network, where each of the edges is weighted and labeled by edge consistency (EC), defined as the number of tumor types that hold the particular edge.

A

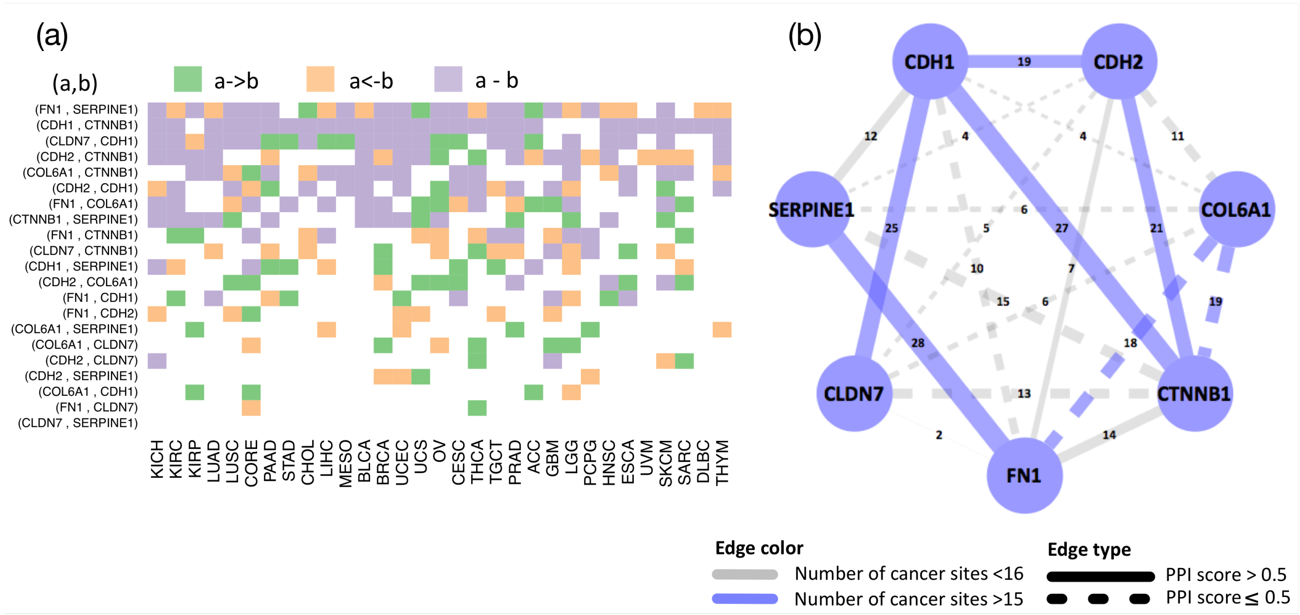

B

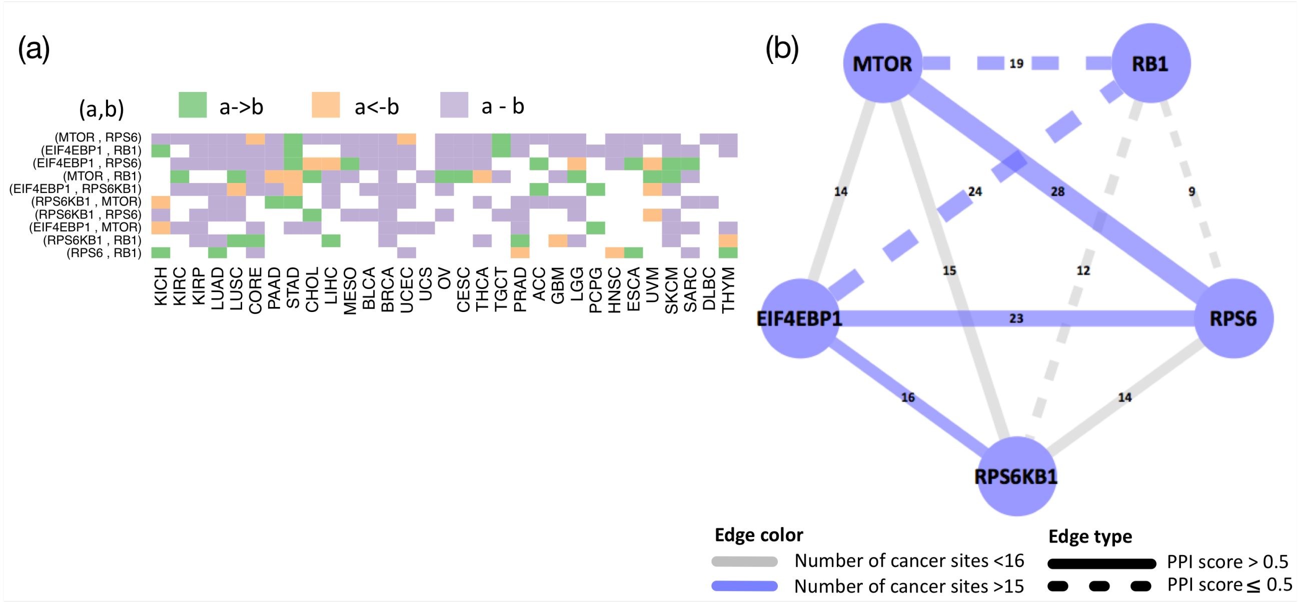

[Supplementary Figure S4] Cancer-specific networks for EMT (A) and TSC/mTOR (B) pathways. (a Heatmap depicting regulatory (-> or <-) and correlative (-) edges across all tumor lineages. (b) Network, where each of the edges is weighted and labeled by edge consistency (EC), defined as the number of tumor types that hold the particular edge.

A

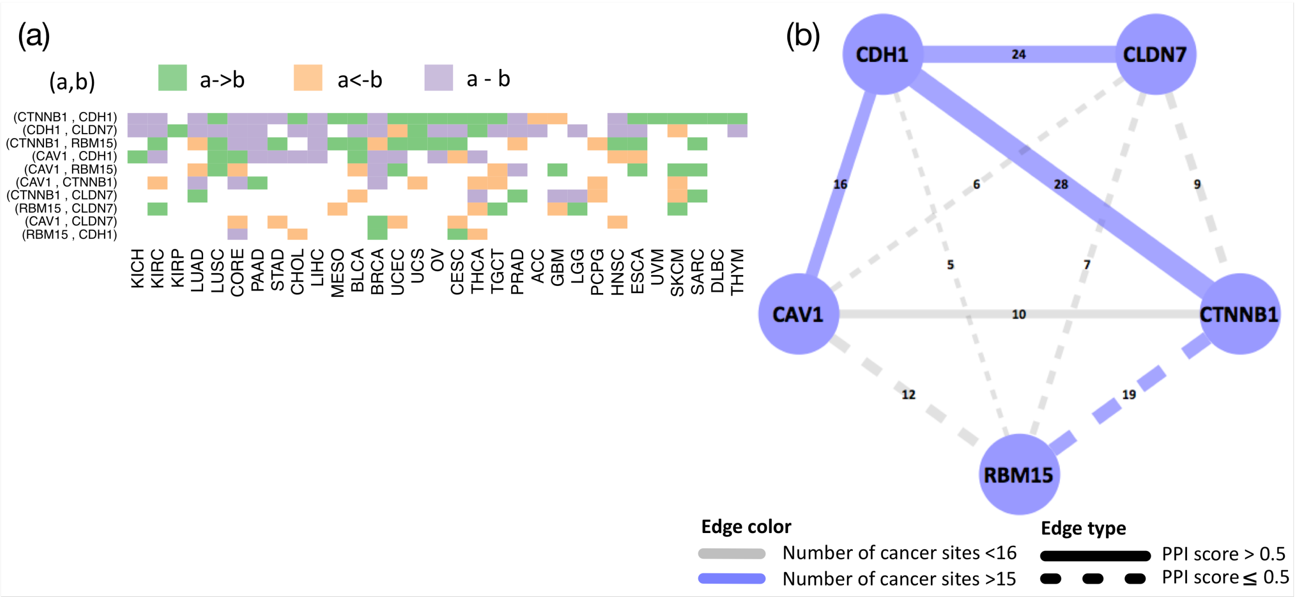

B

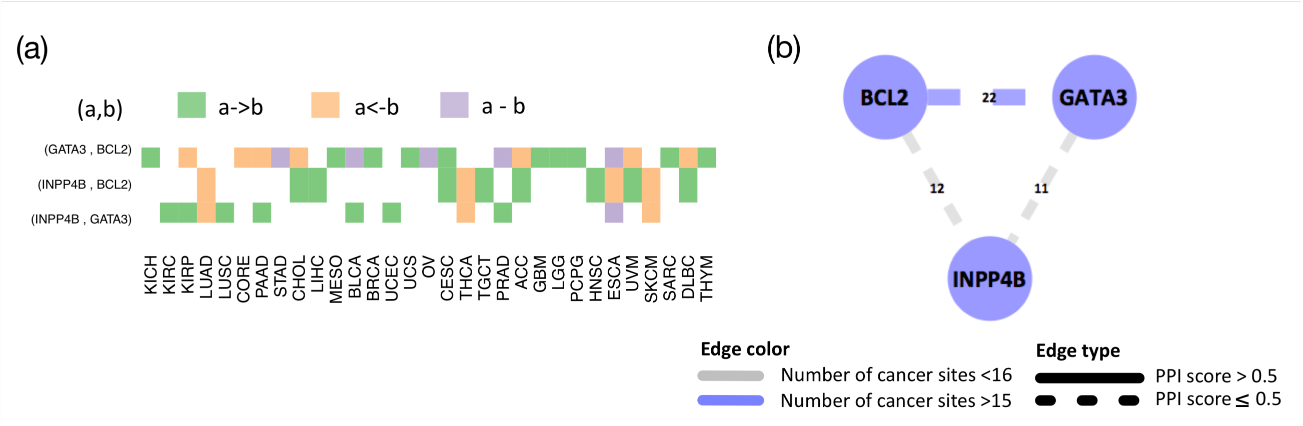

[Supplementary Figure S5] Cancer-specific networks for core reactive (A) and hormone signaling (breast) (B) pathways. (a) Heatmap depicting regulatory (-> or <-) and correlative (-) edges across all tumor lineages. (b) Network, where each of the edges is weighted and labeled by edge consistency (EC), defined as the number of tumor types that hold the particular edge.

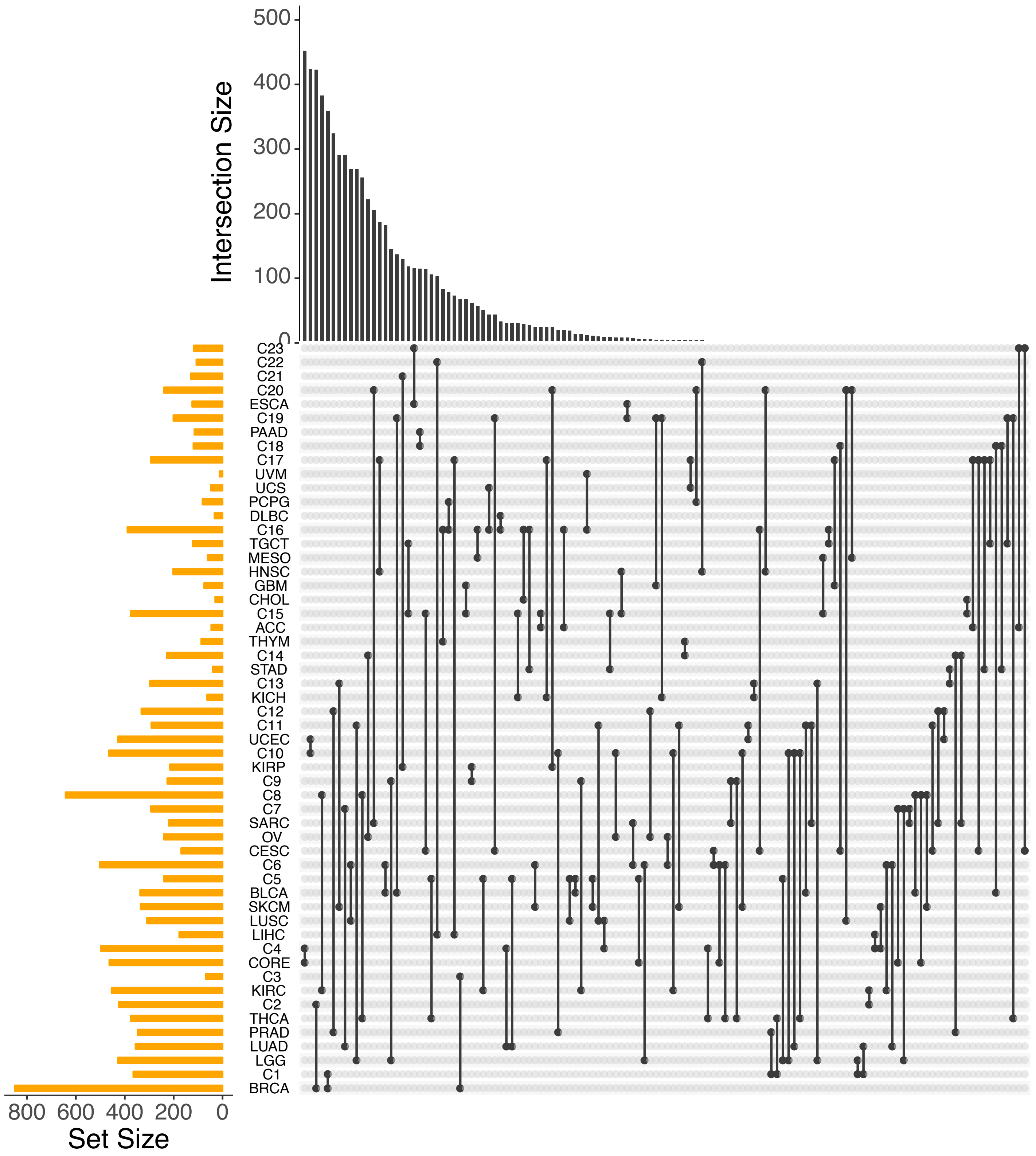

[Supplementary Figure S6] UpSet plot for two-way intersections between clusters obtained by PRECISE and tumor types

A

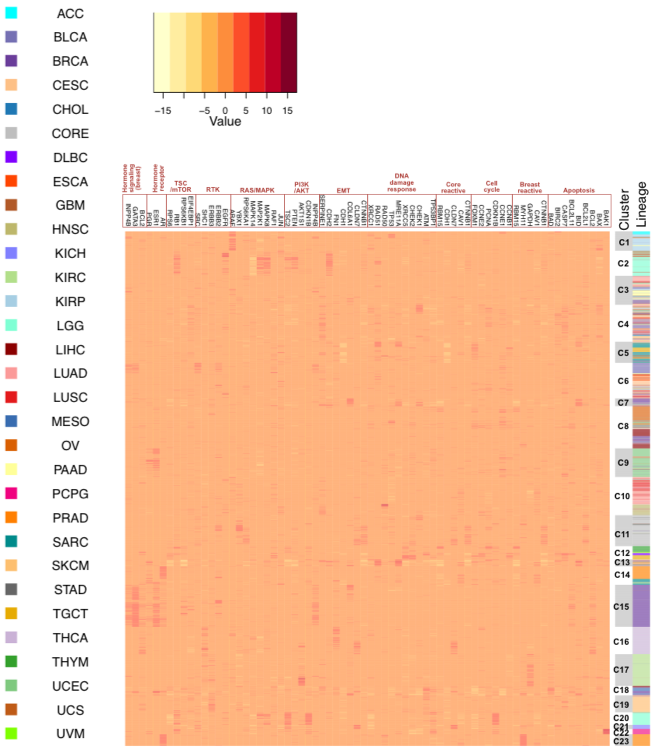

B

Cancer code	C1	C2	C3	C4	C5	C6	C7	C8	C9	C10	C11	C12	C13	C14	C15	C16	C17	C18	C19	C20	C21	C22	C23	Total
KICH	0	0	0	0	0	0	0	5	1	0	0	0	0	0	0	0	0	2	0	0	55	0	0	63
KIRC	1	0	21	2	4	1	4	0	0	0	0	0	2	0	0	0	419	0	0	0	0	0	0	454
KIRP	184	0	21	1	1	2	2	4	0	0	0	0	0	0	0	0	0	0	0	0	0	0	0	215
LUAD	1	0	88	41	1	16	2	4	1	200	0	0	2	0	0	0	0	0	0	0	0	0	0	356
LUSC	1	0	27	76	0	45	3	4	0	148	0	0	4	0	0	0	1	0	0	0	0	0	0	309
CORE	0	0	26	32	1	27	0	1	0	2	373	0	0	0	1	0	0	0	0	0	0	0	0	463
PAAD	11	0	81	2	1	11	0	1	0	0	3	0	4	0	0	0	0	1	0	0	0	0	0	115
STAD	0	0	1	3	0	3	0	2	1	0	30	0	0	0	0	0	0	0	0	0	0	0	0	40
CHOL	6	0	0	22	1	0	0	0	0	0	0	0	1	0	0	0	0	0	0	0	0	0	0	30
LIHC	0	0	0	8	1	0	0	144	4	0	0	0	1	0	0	0	0	19	0	0	0	0	0	177
MESO	0	0	0	1	0	0	0	0	0	0	0	0	0	0	0	0	0	52	0	0	0	0	0	53
BLCA	0	0	35	94	3	161	9	14	2	4	1	2	11	1	0	0	0	0	0	0	0	0	0	337
BRCA	1	0	42	12	0	3	68	96	10	5	2	0	3	0	555	1	1	51	0	0	0	0	0	850
UCEC	0	0	4	16	2	6	2	40	337	0	1	1	0	16	0	0	1	0	2	0	0	0	0	428
UCS	0	0	0	1	30	3	0	7	4	0	0	0	2	0	0	0	0	1	0	0	0	0	0	48
OV	0	3	7	7	1	2	0	211	7	0	0	0	0	1	1	0	0	0	0	0	0	0	0	240
CESC	0	0	0	33	2	101	0	11	4	0	0	0	17	1	0	0	0	0	0	0	0	0	0	169
THCA	3	0	4	0	1	1	2	0	1	2	0	0	0	0	0	362	0	0	0	0	0	0	0	376
TGCT	0	0	1	0	65	0	0	1	0	0	0	55	0	0	0	0	0	0	0	0	0	0	0	122
PRAD	8	0	0	1	2	0	1	2	0	0	0	0	7	169	0	0	0	0	0	0	0	0	157	347
ACC	40	1	2	2	1	0	0	0	0	0	0	0	0	0	0	0	0	0	0	0	0	0	0	46
GBM	0	67	0	0	0	0	4	0	0	0	0	0	0	0	0	0	0	0	1	3	0	0	0	75
LGG	0	260	0	0	0	0	0	0	1	0	0	0	1	0	0	0	0	2	0	164	0	0	0	428
PCPG	0	0	0	1	11	0	0	0	0	0	0	0	1	0	0	0	0	0	0	0	0	69	0	82
HNSC	0	0	1	42	2	6	1	5	1	144	0	0	0	0	0	0	0	0	0	0	0	0	0	202
ESCA	0	0	0	20	1	81	0	2	2	1	1	0	16	0	0	0	0	0	0	0	0	0	0	124
UVM	0	0	0	0	0	0	0	0	0	0	0	12	0	0	0	0	0	0	0	0	0	0	0	12
SKCM	1	2	18	71	2	0	4	2	3	2	0	1	9	1	0	0	0	0	219	0	0	0	0	335
SARC	0	0	2	1	146	3	0	0	1	0	0	0	10	57	0	0	0	0	0	0	0	0	0	220
DLBC	0	0	0	2	0	1	0	0	0	0	0	29	0	0	0	0	0	0	1	0	0	0	0	33
THYM	1	0	3	1	1	0	0	5	0	0	0	76	0	0	0	0	0	0	0	0	0	0	0	87
Total	258	333	384	492	280	473	102	561	380	508	411	176	91	246	557	363	422	128	223	167	55	69	157	6836

[Supplementary Figure S7] Pan-cancer stratification using RPPA data only. (A) Heatmap depicting protein levels after unsupervised hierarchical clustering of 6,836 cancer samples across 32 tumor types and proteins included in the 12 pathways. Protein levels are indicated on a low-to-high scale (yellow-to-red). 23 clusters are defined. Annotation bars are included in the vertical lines for the 23 clusters and tumor lineages. (B) Crosstab showing the number of tumor samples in each cluster.

A

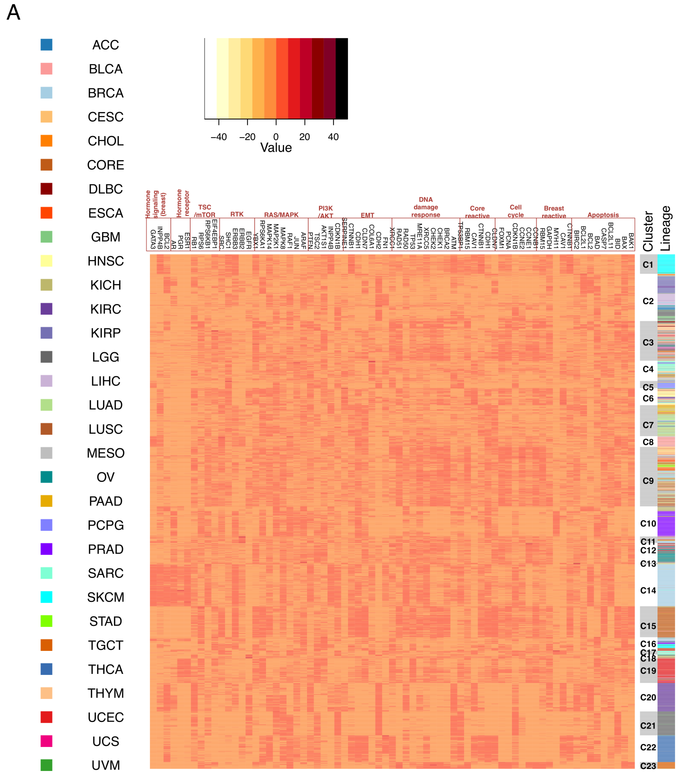

B

Cancer Code	C1	C2	C3	C4	C5	C6	C7	C8	C9	C10	C11	C12	C13	C14	C15	C16	C17	C18	C19	C20	C21	C22	C23
KICH	0	4	0	0	1	1	0	0	0	56	0	0	0	0	0	1	0	0	0	0	0	0	0
KIRC	0	30	2	13	0	24	4	0	4	8	0	0	0	0	0	3	0	0	0	366	0	0	0
KIRP	0	192	3	1	0	0	2	2	2	1	0	0	0	0	0	0	0	0	0	10	0	2	0
LUAD	1	1	12	5	4	9	250	1	58	0	3	1	0	0	5	0	0	0	2	0	0	2	2
LUSC	0	1	35	3	0	11	12	3	207	0	2	19	0	1	5	0	0	8	2	0	0	0	0
CORE	0	0	17	1	0	7	10	3	17	2	7	1	0	0	381	16	0	0	1	0	0	0	0
PAAD	0	1	4	26	2	0	73	1	4	1	0	0	0	1	2	0	0	0	0	0	0	0	0
STAD	0	0	0	0	0	0	1	0	36	0	2	0	0	0	0	0	0	1	0	0	0	0	0
CHOL	0	15	3	0	3	1	4	0	0	0	0	0	0	0	2	1	0	0	0	1	0	0	0
LIHC	0	154	7	1	0	1	0	0	2	0	0	2	0	0	0	5	0	0	5	0	0	0	0
MESO	0	7	0	36	0	6	1	0	1	0	0	0	0	0	0	0	0	0	2	0	0	0	0
BLCA	1	2	81	9	2	30	6	125	41	5	8	8	4	1	6	3	5	0	0	0	0	0	0
BRCA	2	6	57	11	0	2	16	3	111	7	43	7	0	548	0	31	0	3	3	0	0	0	0
UCEC	0	2	38	4	0	0	9	0	8	0	7	44	0	0	1	21	0	0	294	0	0	0	0
UCS	0	0	25	2	0	0	0	0	6	0	0	10	0	0	0	0	0	0	5	0	0	0	0
OV	0	11	45	3	0	1	4	0	2	1	2	164	1	0	1	2	0	0	3	0	0	0	0
CESC	0	4	25	0	0	4	2	1	111	0	5	0	7	1	4	1	0	1	3	0	0	0	0
THCA	0	0	1	3	0	0	6	0	0	0	0	2	0	0	0	14	0	0	1	0	0	349	0
TGCT	0	1	25	2	0	0	0	0	0	3	0	0	0	0	1	0	1	0	1	0	0	0	88
PRAD	0	0	0	6	0	0	4	0	1	314	1	0	0	0	0	19	0	0	1	1	0	0	0
ACC	1	38	3	1	1	1	1	0	0	0	0	0	0	0	0	0	0	0	0	0	0	0	0
GBM	0	47	1	3	0	0	3	0	3	0	0	0	0	0	0	1	0	11	0	0	6	0	0
LGG	0	100	0	1	1	0	0	0	0	0	0	0	0	0	0	0	0	15	0	0	311	0	0
PCPG	0	0	0	0	78	1	0	0	0	1	0	0	0	0	0	1	0	0	1	0	0	0	0
HNSC	0	1	3	16	0	100	1	1	62	0	1	2	5	0	1	0	0	8	1	0	0	0	0
ESCA	0	0	7	2	0	10	0	1	91	0	5	1	0	0	2	0	1	3	1	0	0	0	0
UVM	4	0	3	0	0	0	0	0	0	0	0	0	0	0	0	5	0	0	0	0	0	0	0
SKCM	250	3	10	4	1	4	1	0	9	0	0	0	2	1	0	49	0	0	1	0	0	0	0
SARC	0	8	15	110	1	15	1	2	7	1	0	2	1	3	0	5	46	0	0	1	0	2	0
DLBC	0	0	33	0	0	0	0	0	0	0	0	0	0	0	0	0	0	0	0	0	0	0	0
THYM	0	0	72	6	0	0	0	0	7	0	0	1	0	1	0	0	0	0	0	0	0	0	0
Total	259	628	527	269	94	228	411	143	790	400	86	264	20	557	411	178	53	50	327	379	317	355	90

[Supplementary Figure S8] Pan-cancer stratification using mRNA expression data only. (A) Heatmap depicting mRNA expression levels after unsupervised hierarchical clustering of 6,836 cancer samples across 32 tumor types and genes included in the 12 pathways. Gene expression levels are indicated on a low-to-high scale. 23 clusters are defined. Annotation bars are included in the vertical lines for the 23 clusters and tumor lineages. (B) Crosstab showing the number of tumor samples in each cluster.

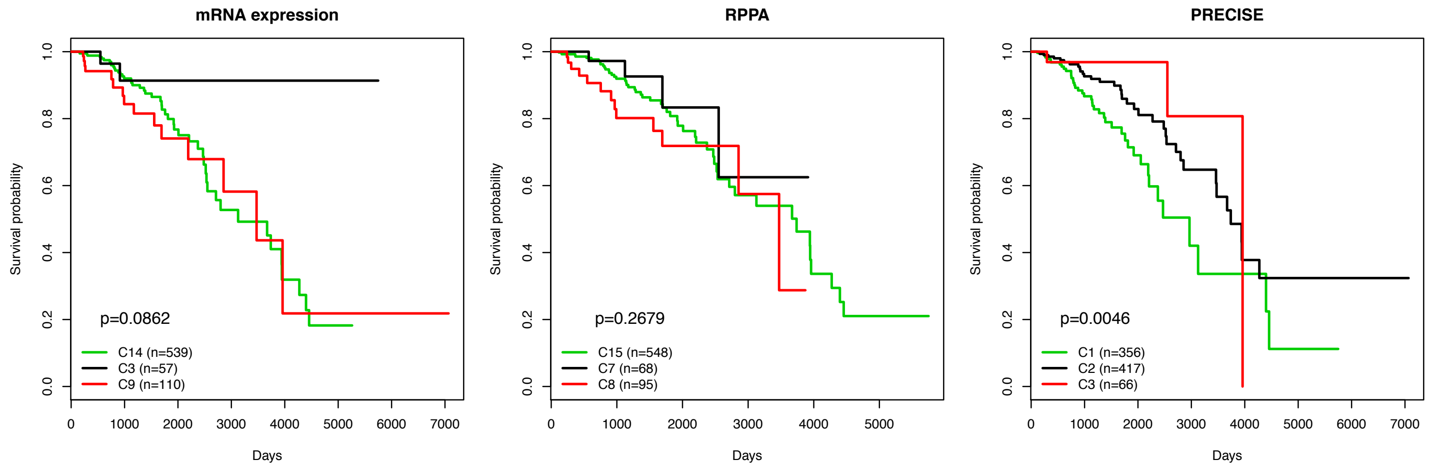

[Supplementary Figure S9] Kaplan-Meier curves for clusters obtained from mRNA expression, RPPA, and PRECISE scores for BRCA. The sample sizes are computed after clinical data are matched.

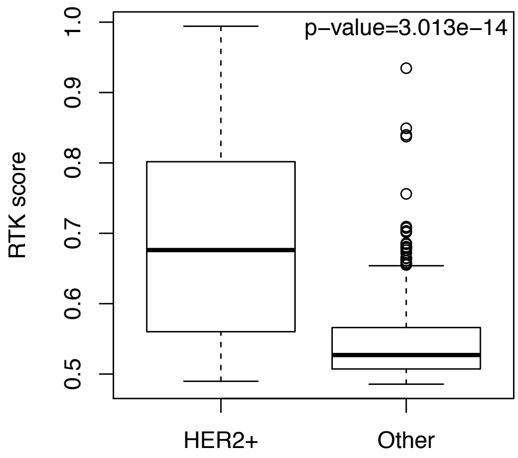

[Supplementary Figure S10] Boxplots of PRECISE RTK scores for HER2 + versus other BRCA patients.

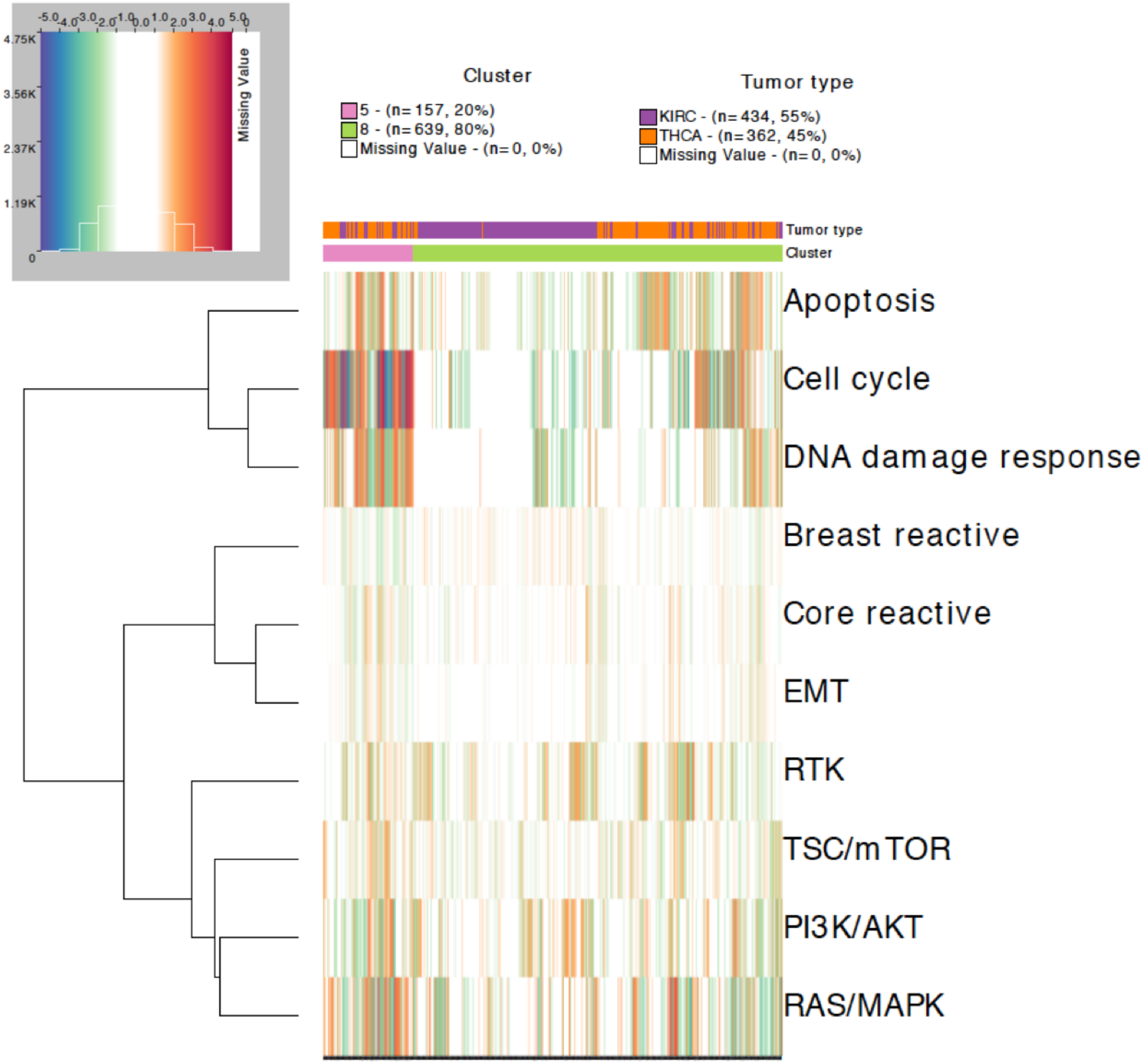

[Supplementary Figure S11] Heatmap depicting combined activated and suppressed PRECISE scores for clusters identified on KIRC and THCA patients. The combined scores are signed by PRECISE statuses, -1 (suppressed), 1 (activated), or 0 (neutral). The signed scores are indicated on a suppressed-neutral-activated scale (blue-white-red). Annotation bars are in the horizontal lines for clusters C5 and C8 and tumor types.

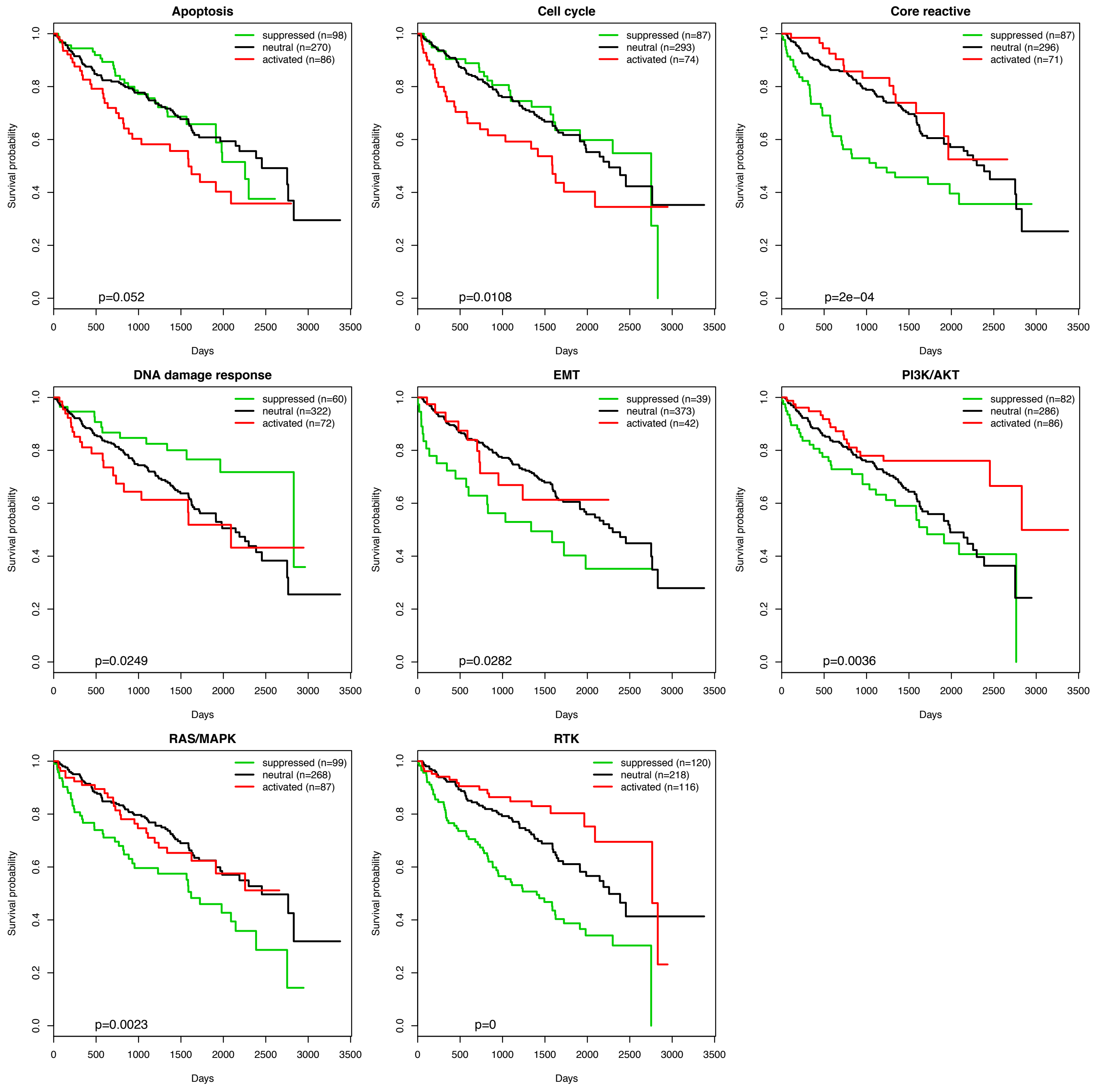

[Supplementary Figure S12] Kaplan-Meier curves for the three groups of KIRC patients, defined by PRECISE scores as suppressed (green), neutral (black), and activated (red) for pathways with FDR < 0.1.

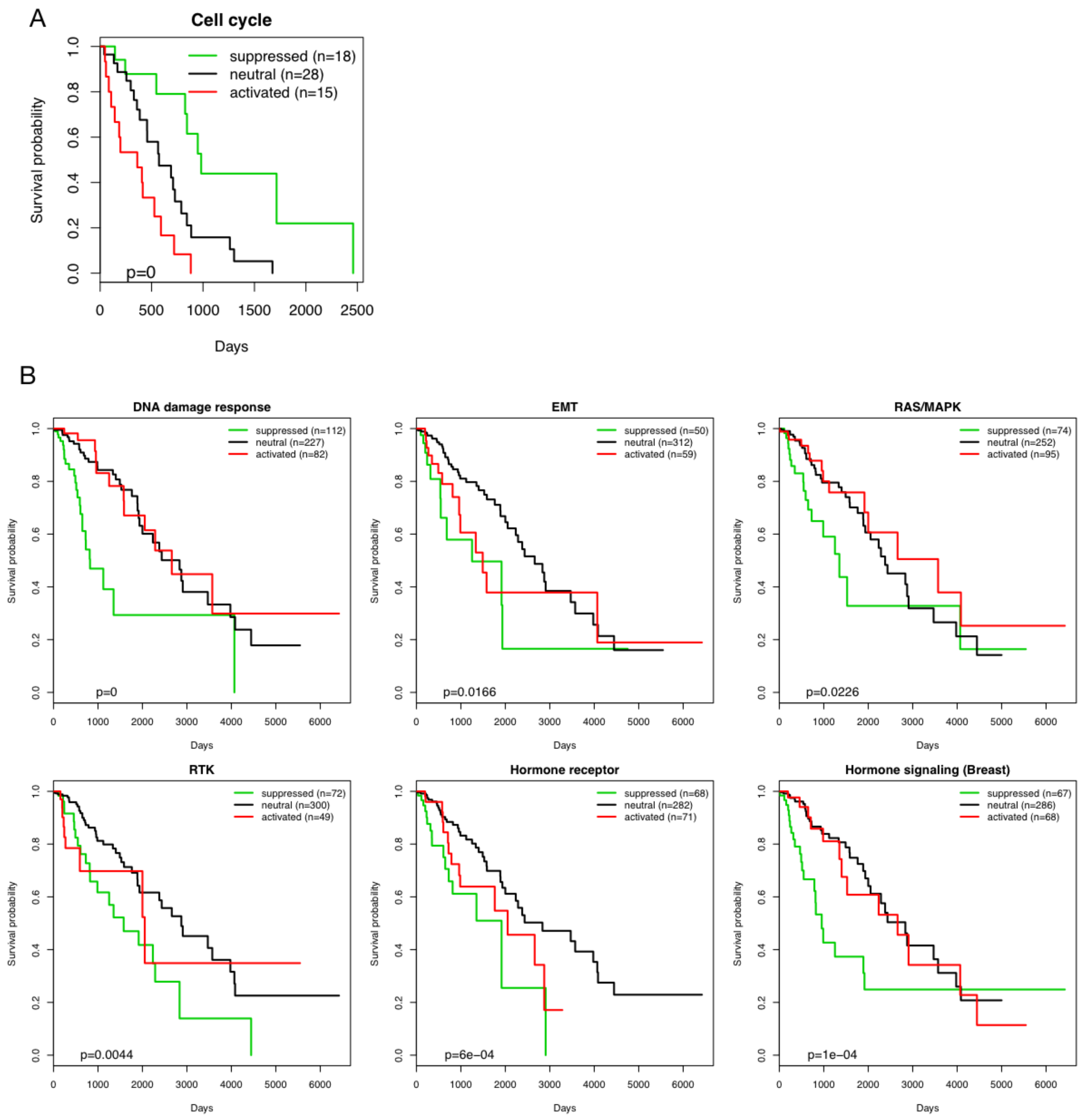

[Supplementary Figure S13] Kaplan-Meier curves for the three groups of MESO (A) and LGG (B) patients, defined by PRECISE scores as suppressed (green), neutral (black), and activated (red) for pathways with FDR < 0.1.

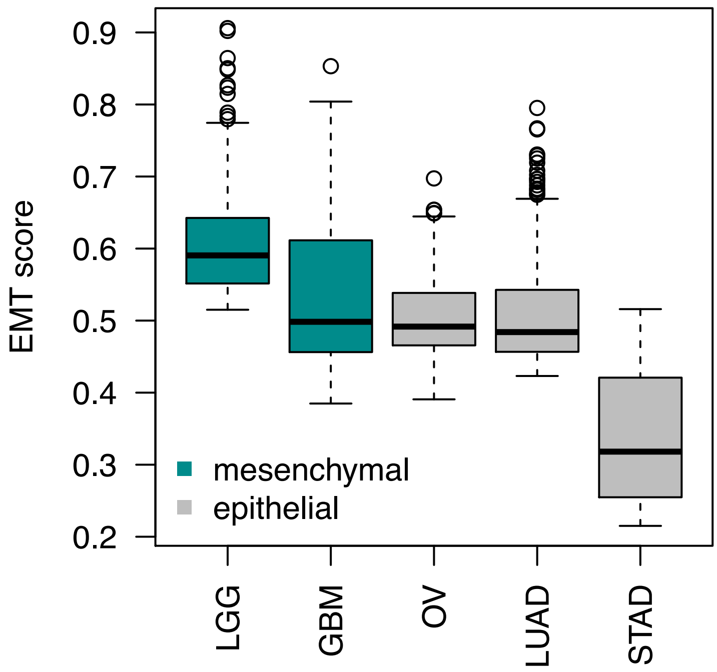

[Supplementary Figure S14] Boxplots of PRECISE EMT scores across mesenchymal tumors (LGG and GBM) and epithelial tumors (OV, LUAD, and STAD).

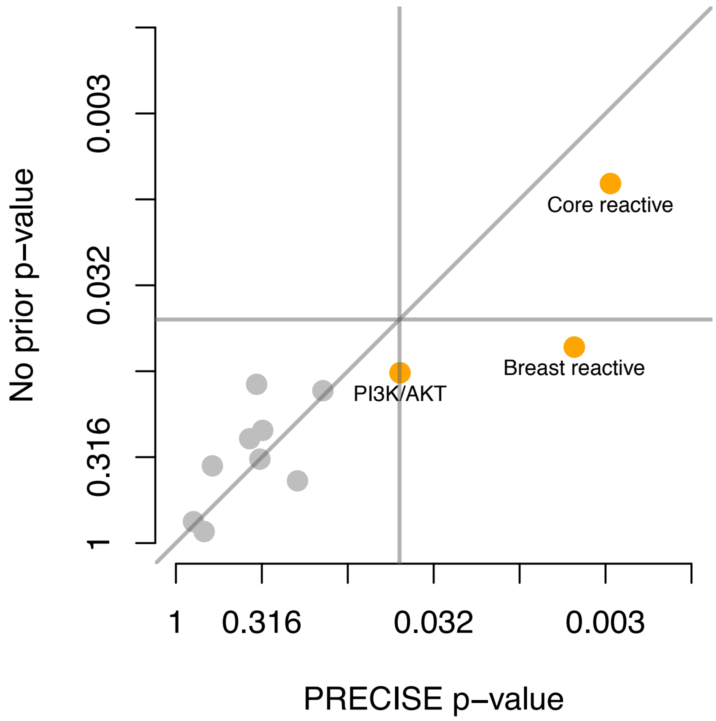

[Supplementary Figure S15] Scatterplot of –log10 p-values of PRECISE versus PRECISE with no prior for BRCA patients. The vertical and horizontal lines indicate p-values at 0.05.

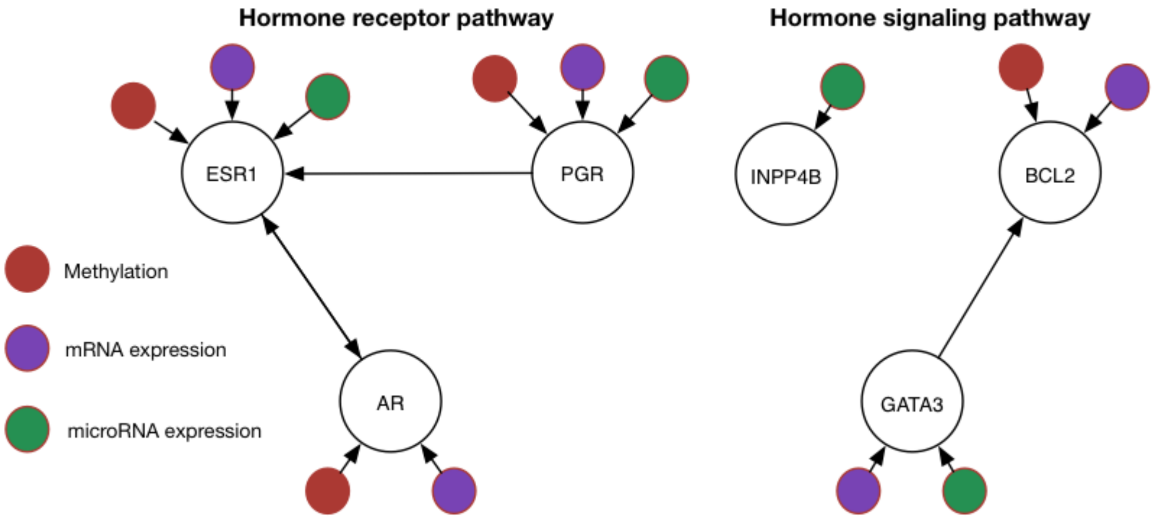

[Supplementary Figure S16] PRECISE Hormone receptor and Hormone signaling pathways for BRCA patients. For the same pathways, PRECISE with no prior and no upstream data provided empty networks.

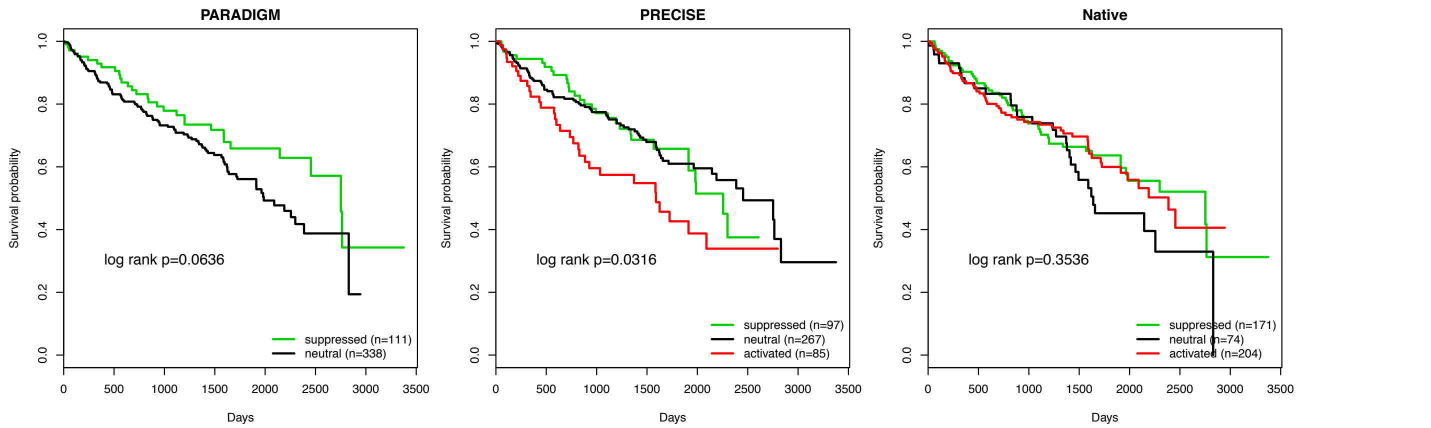

[Supplementary Figure S17] Kaplan-Meier curves of PARADIAM, PRECISE, and Native scores of apoptosis pathway for KIRC patients.

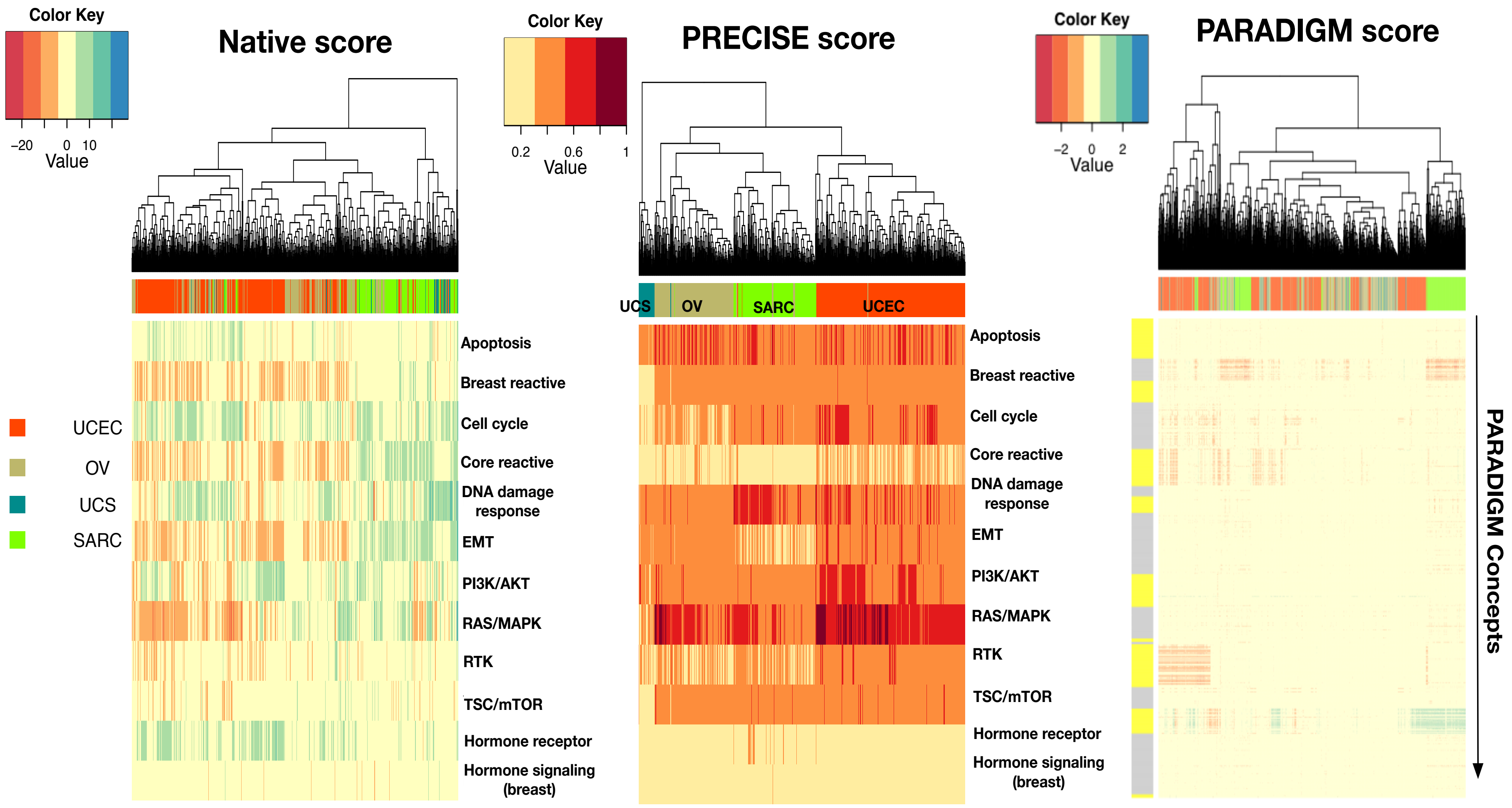

[Supplementary Figure S18] Heatmaps for Native, PRECISE and PARADIGM scores for UCEC, OV, UCS and SARC patients.

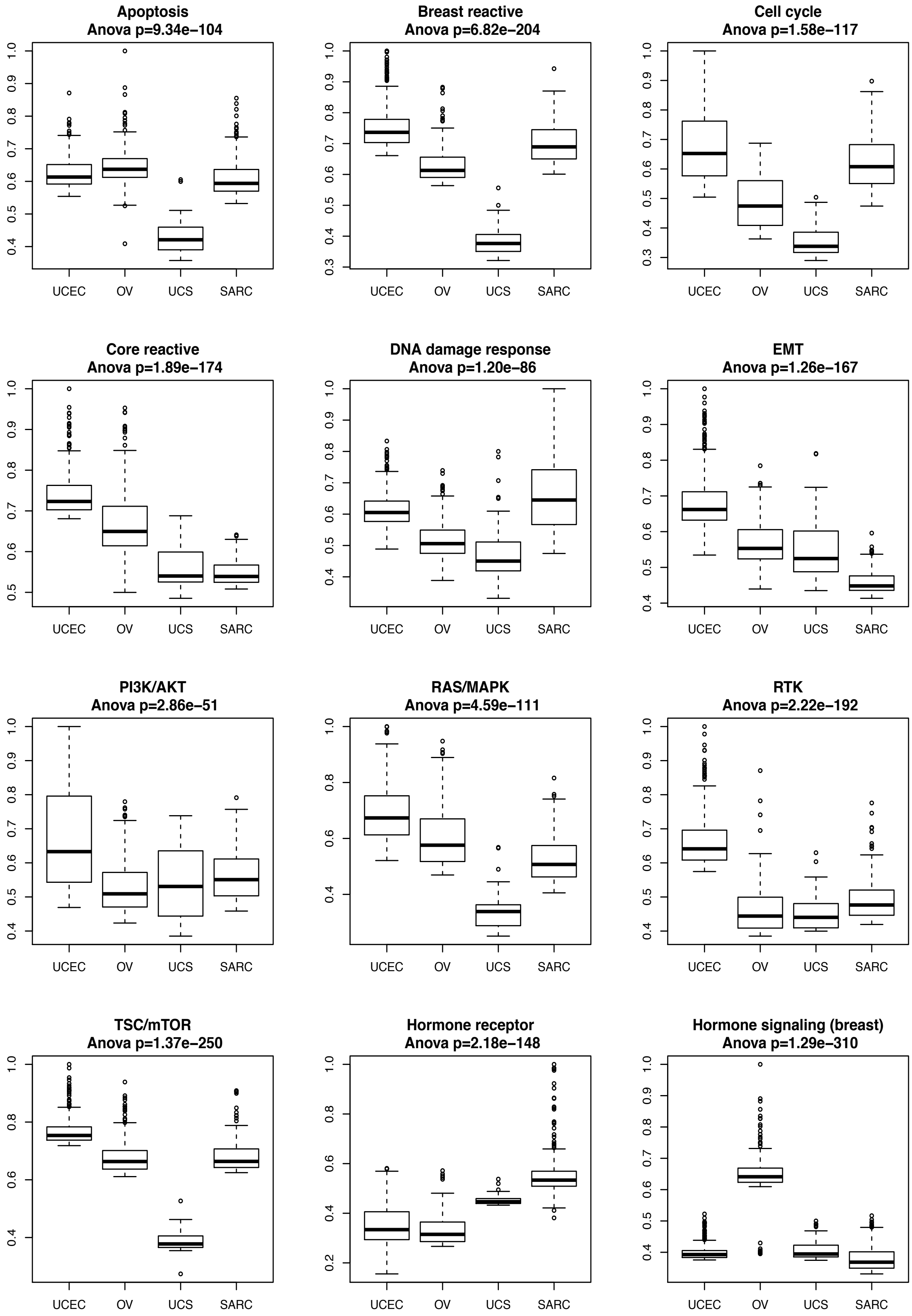

[Supplementary Figure S19] Boxplot of PRECISE pathway activities, showing how UCEC, OV, UCS and SARC are different.

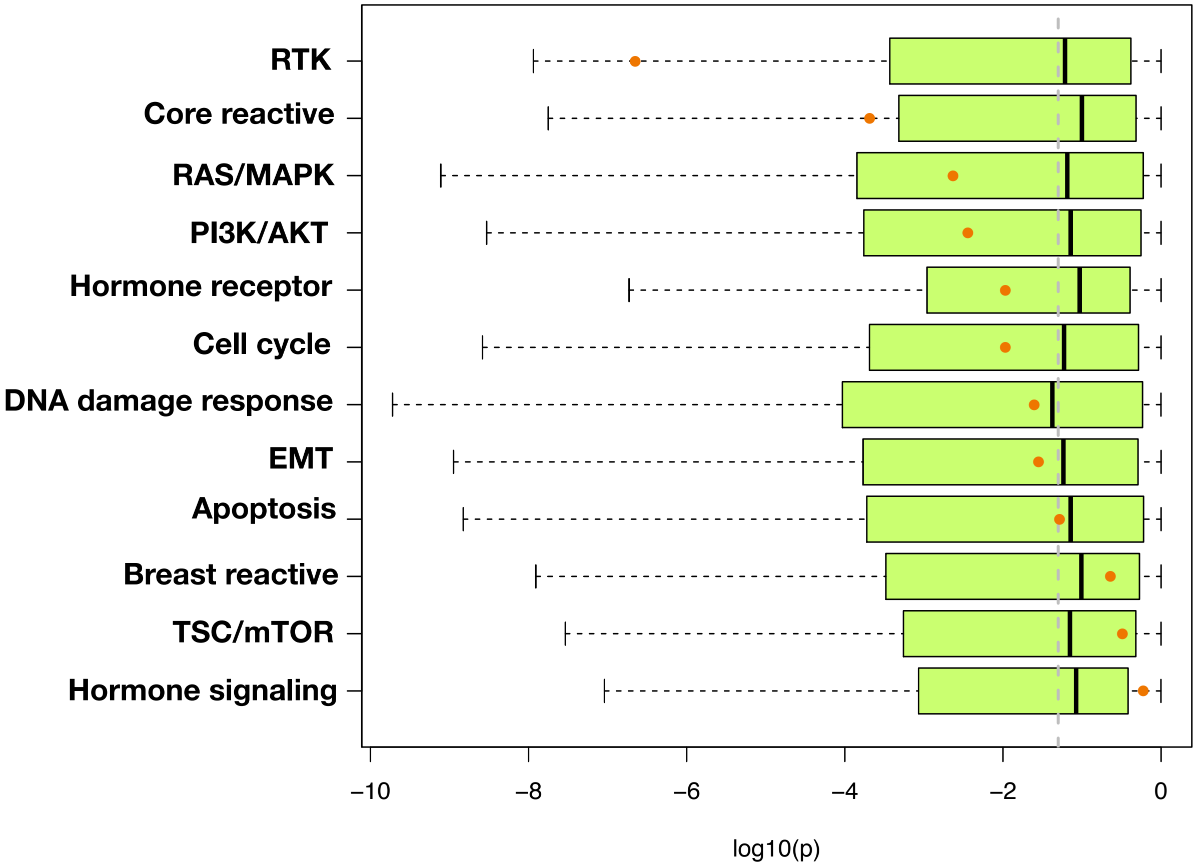

[Supplementary Figure S20] Boxplots of the p-values of associations of 1000 random signatures with overall survival across 12 pathways. Orange dots stand for p-value from PRECISE statuses.

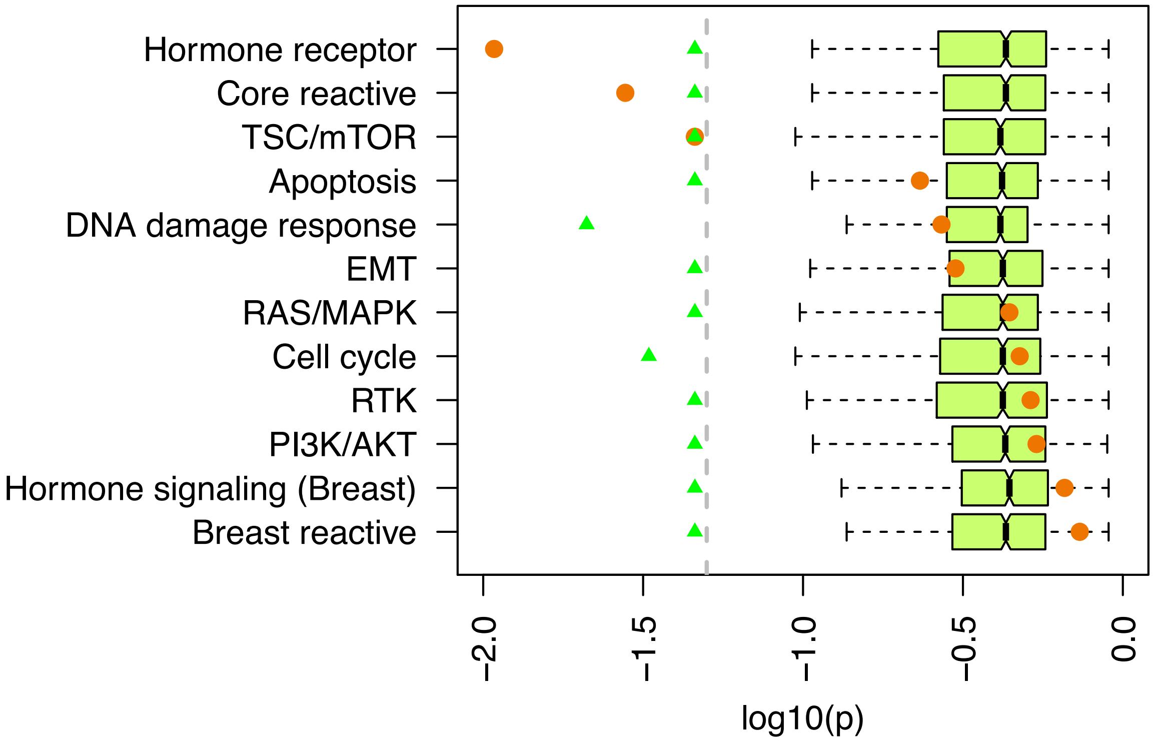

[Supplementary Figure S21] Boxplots of the p-values of associations of 1000 random signatures with overall survival across 12 pathways for DLBC patients. Orange circles stand for p-value from PRECISE statuses and green triangles are the top 50 p-values among the 1000 random signatures. The dashed line displays the location of log10(0.05).

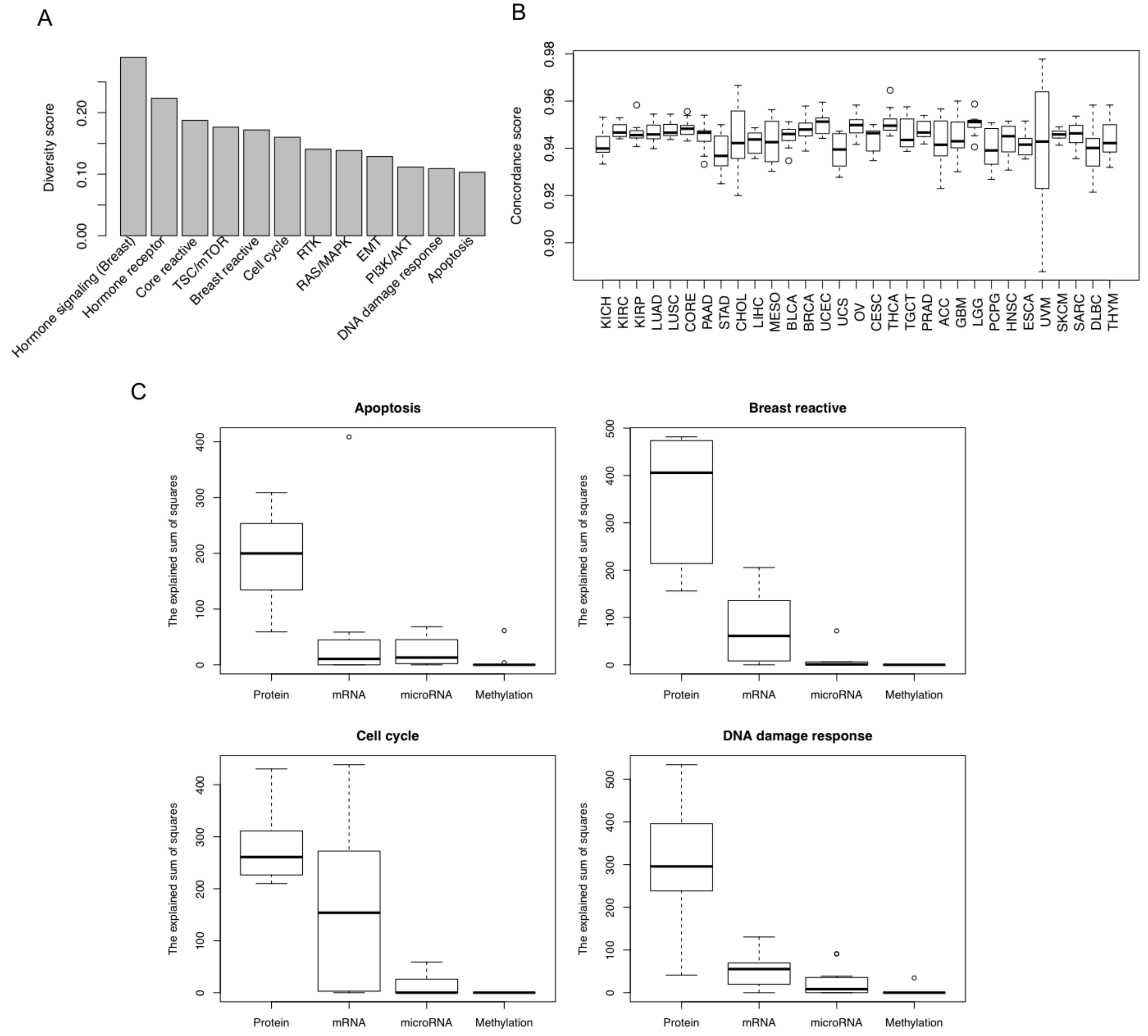

[Supplementary Figure S22] (A) Diversity score, which is defined by standard deviation of connectivity scores (CS) across all cancer lineages; (B) The distribution of concordance scores across 12 pathways for all cancer lineages; (C) The explained sum of squares for each protein in apoptosis, breast reactive, cell cycle, and DNA damage response pathways for BRCA patients.

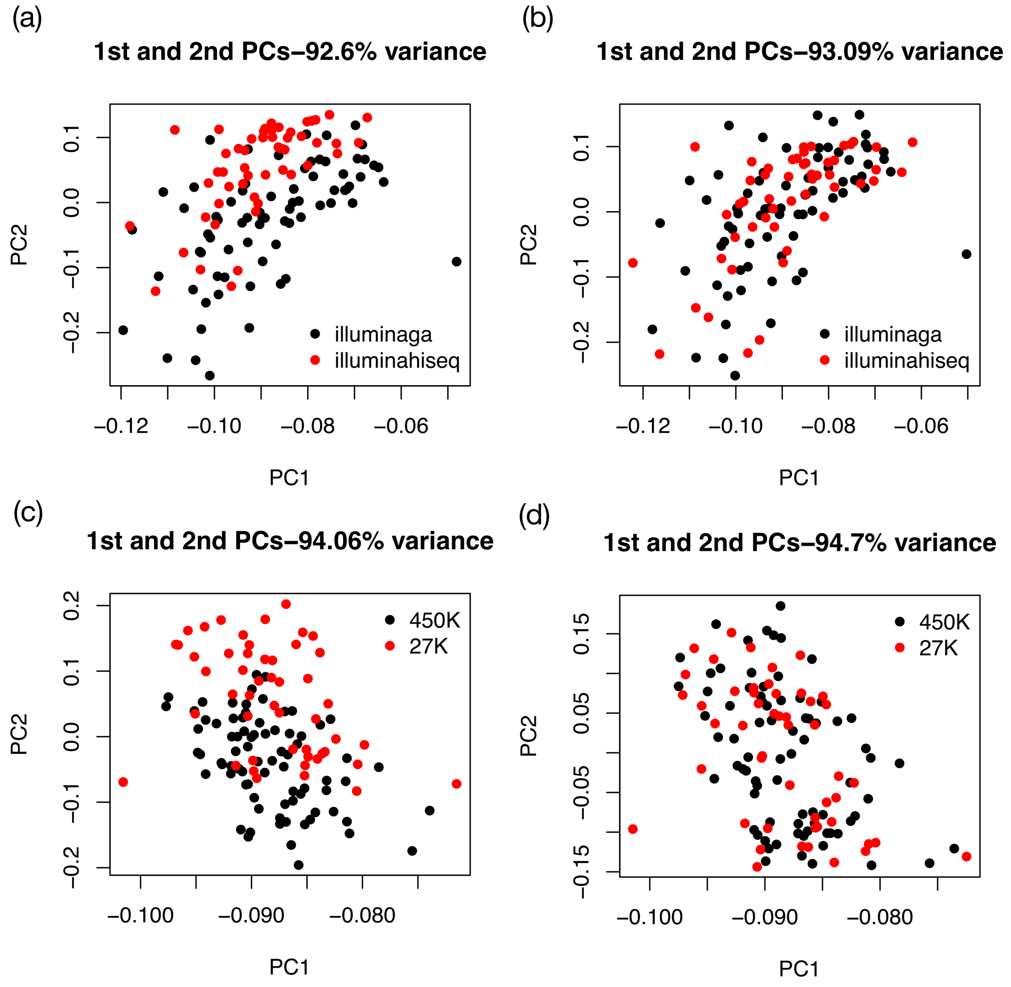

[Supplementary Figure S23] PCA plots of 124 READ samples. (a) microRNA data before adjusting for the batch effect; (b) microRNA data after adjusting for the batch effect; (c) Methylation data before adjusting for the batch effect; (d) Methylation data after adjusting for the batch effect.]

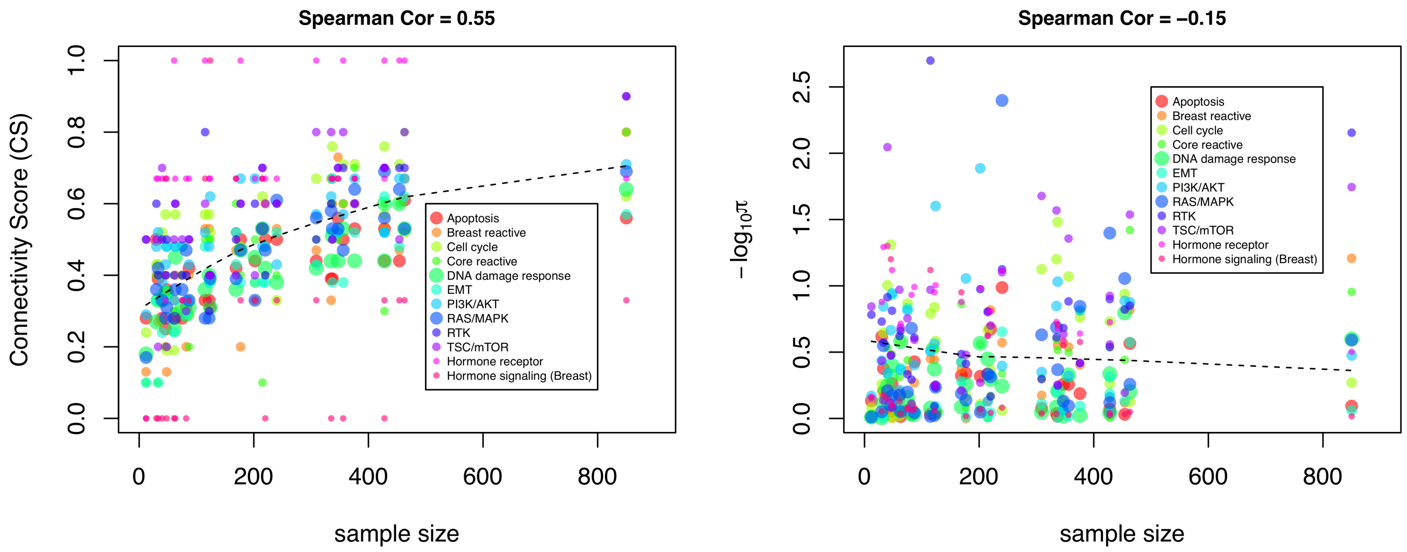

[Supplementary Figure S24] Scatter plot of sample size versus connectivity score (CS) (left) and $-\log_{10} p_{cs}$ (right), where the Spearman correlation is 0.55 and -0.15, respectively. The colors and sizes of the dots correspond to pathways and the sizes (no. of nodes).

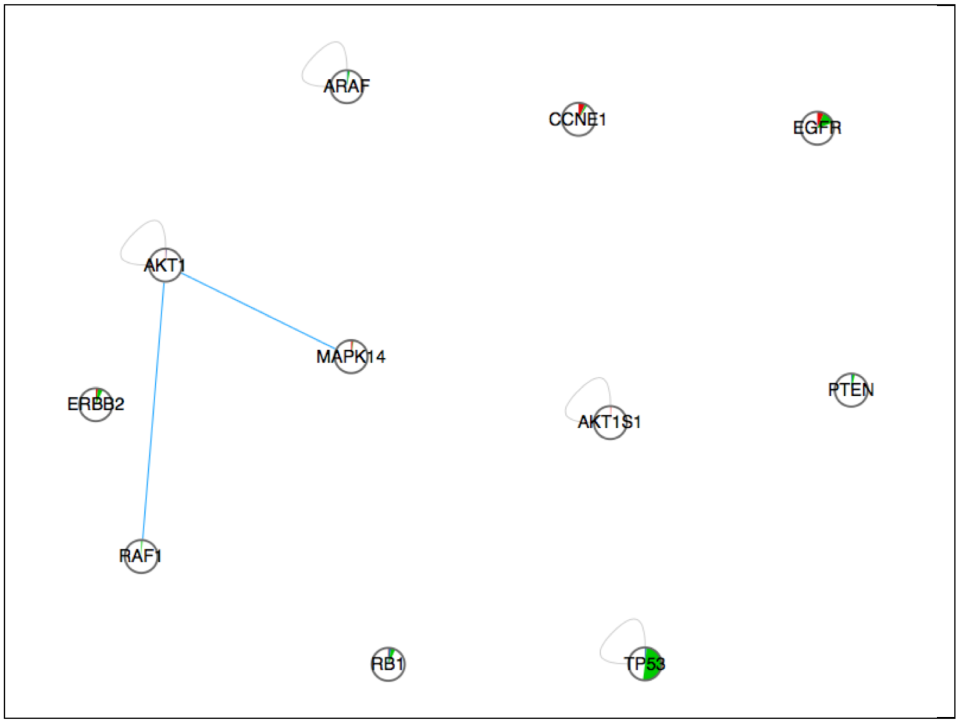

[Supplementary Figure S25] OncoPPI sub-network for the 12 genes.

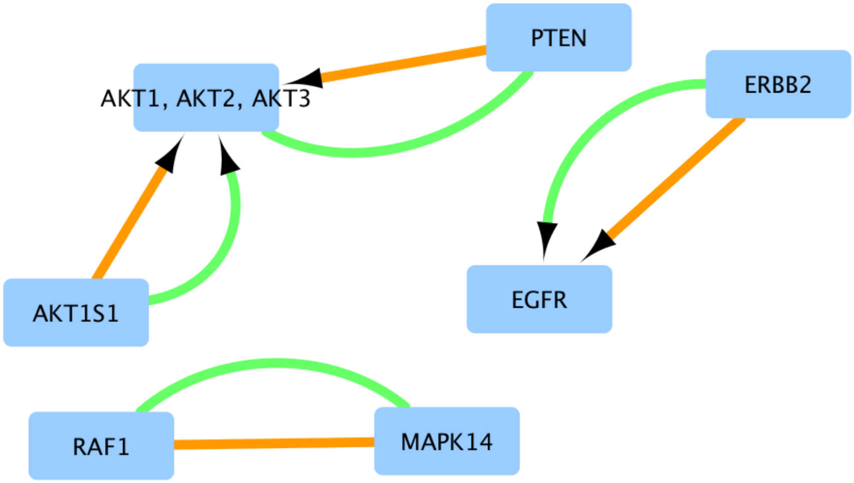

[Supplementary Figure S26] PRECISE network for 12 genes that are included in the OncoPPI data (only genes that have edges are displayed). The orange and green edges represent LUAD and LUSC, respectively.

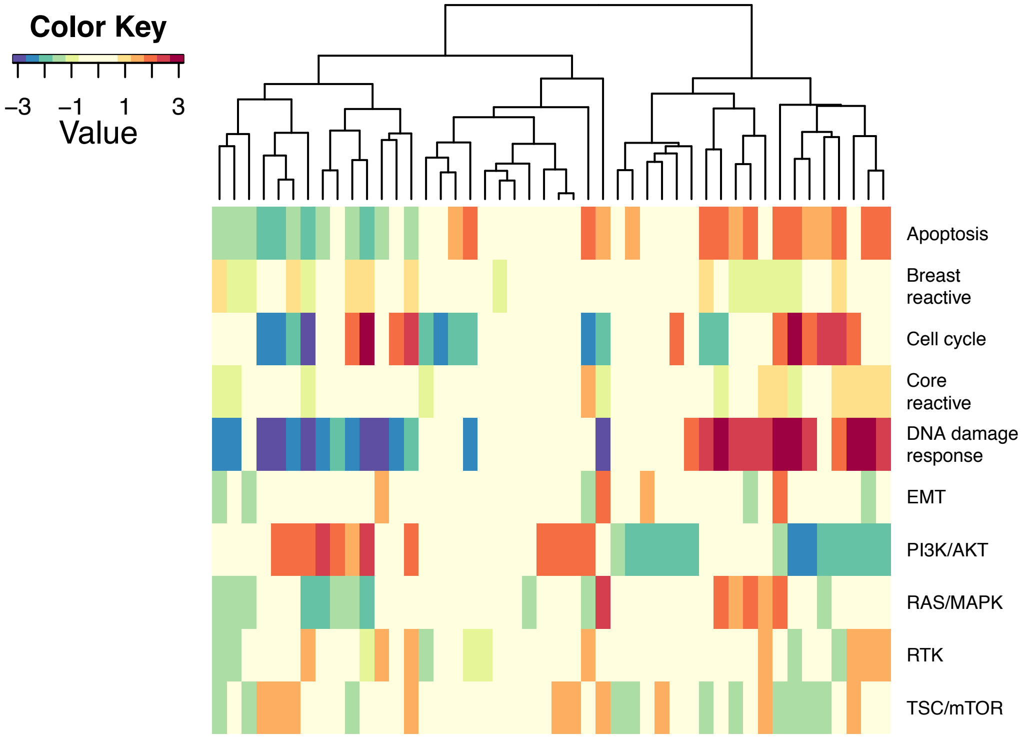

[Supplementary Figure S27] Heatmap depicting combined activated and suppressed PRECISE scores for ACC patients. The combined scores are signed by PRECISE statuses, -1 (suppressed),1 (activated), or 0 (neutral).

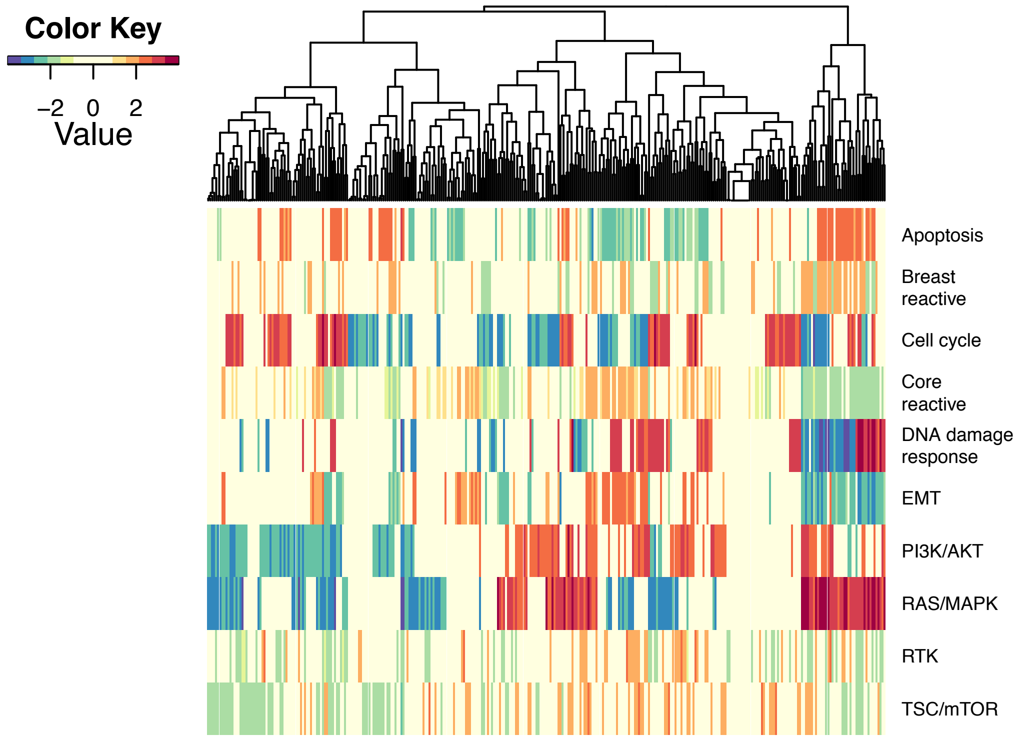

[Supplementary Figure S28] Heatmap depicting combined activated and suppressed PRECISE scores for BLCA patients. The combined scores are signed by PRECISE statuses, -1 (suppressed),1 (activated), or 0 (neutral).

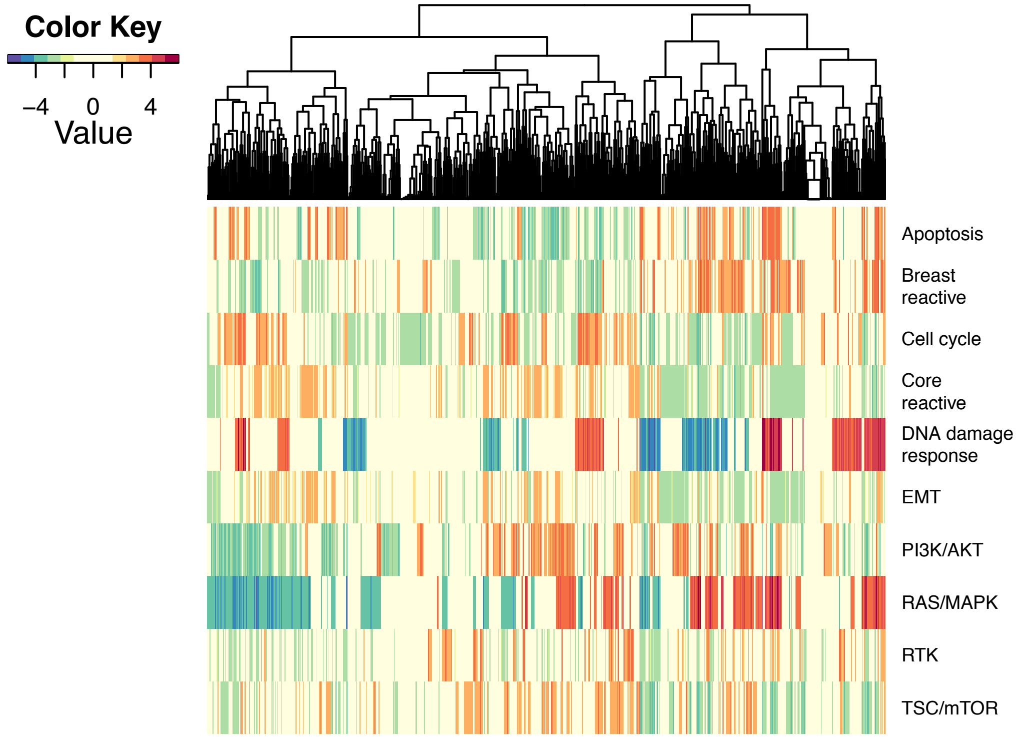

[Supplementary Figure S29] Heatmap depicting combined activated and suppressed PRECISE scores for BRCA patients. The combined scores are signed by PRECISE statuses, -1 (suppressed),1 (activated), or 0 (neutral).

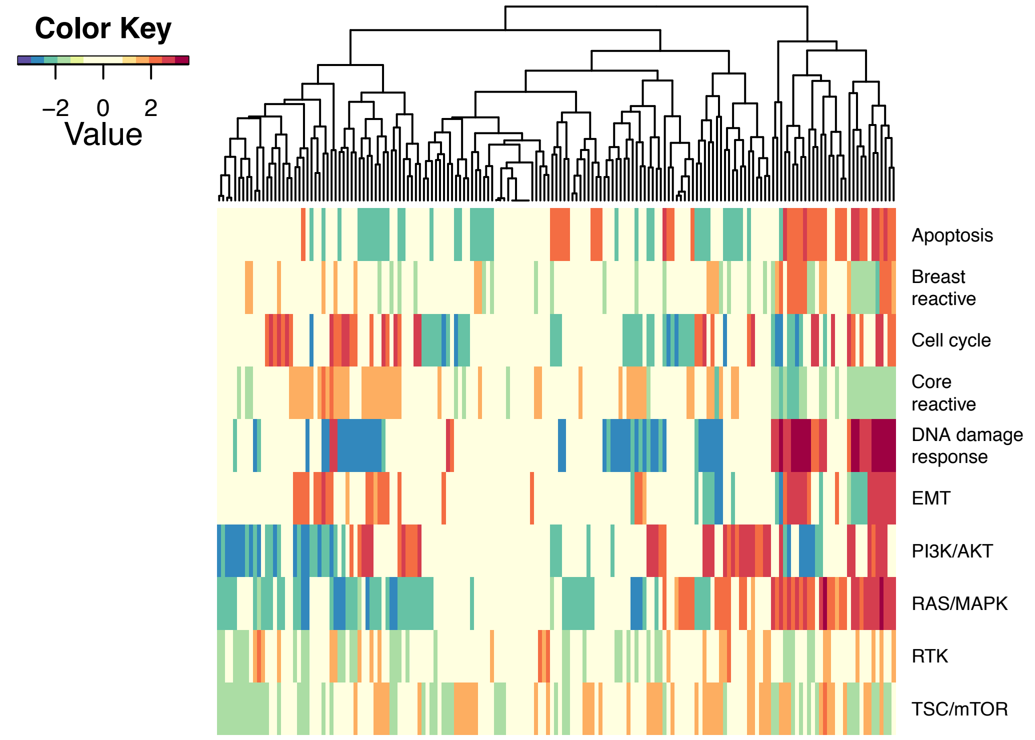

[Supplementary Figure S30] Heatmap depicting combined activated and suppressed PRECISE scores for CESC patients. The combined scores are signed by PRECISE statuses, -1 (suppressed),1 (activated), or 0 (neutral).

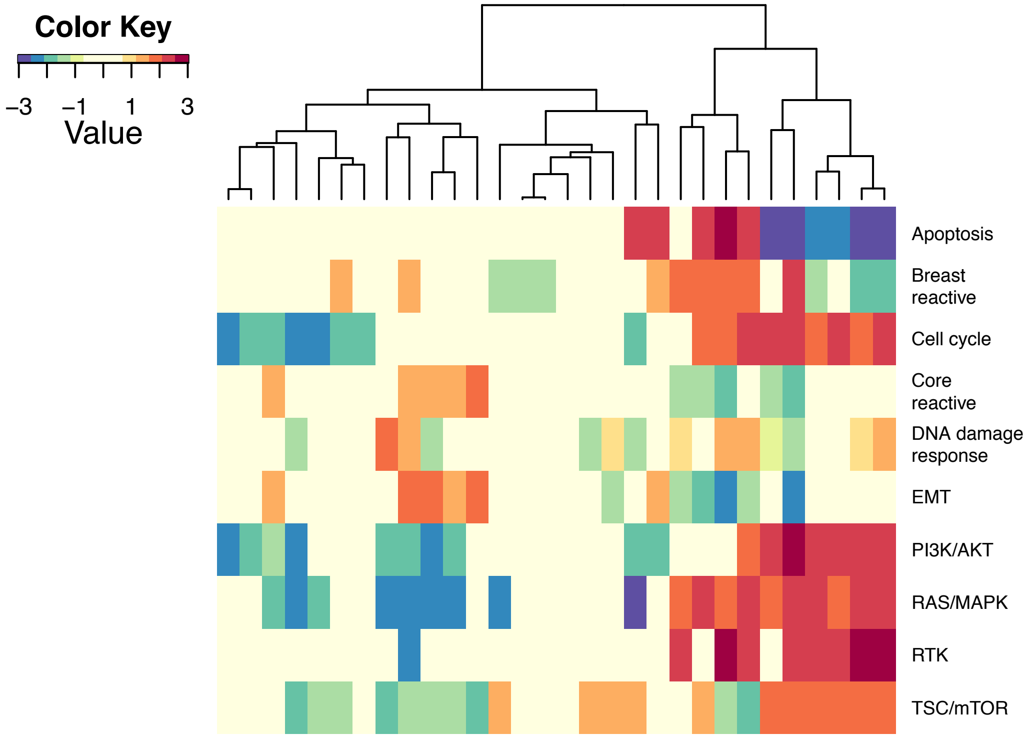

[Supplementary Figure S31] Heatmap depicting combined activated and suppressed PRECISE scores for CHOL patients. The combined scores are signed by PRECISE statuses, -1 (suppressed),1 (activated), or 0 (neutral).

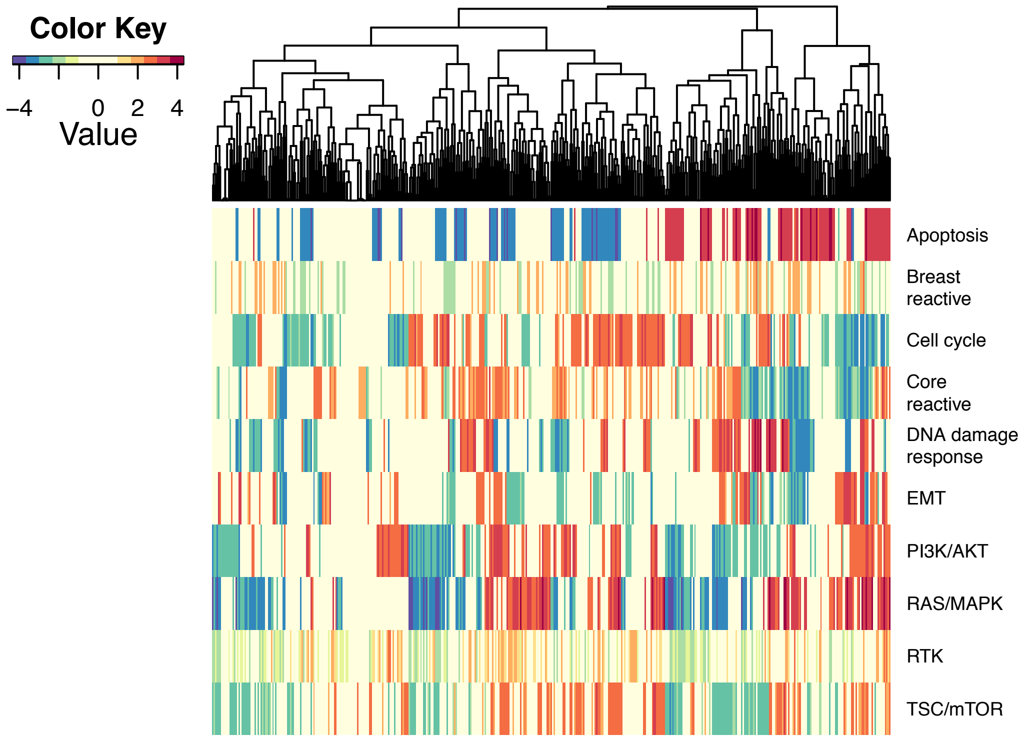

[Supplementary Figure S32] Heatmap depicting combined activated and suppressed PRECISE scores for CORE patients. The combined scores are signed by PRECISE statuses, -1 (suppressed),1 (activated), or 0 (neutral).

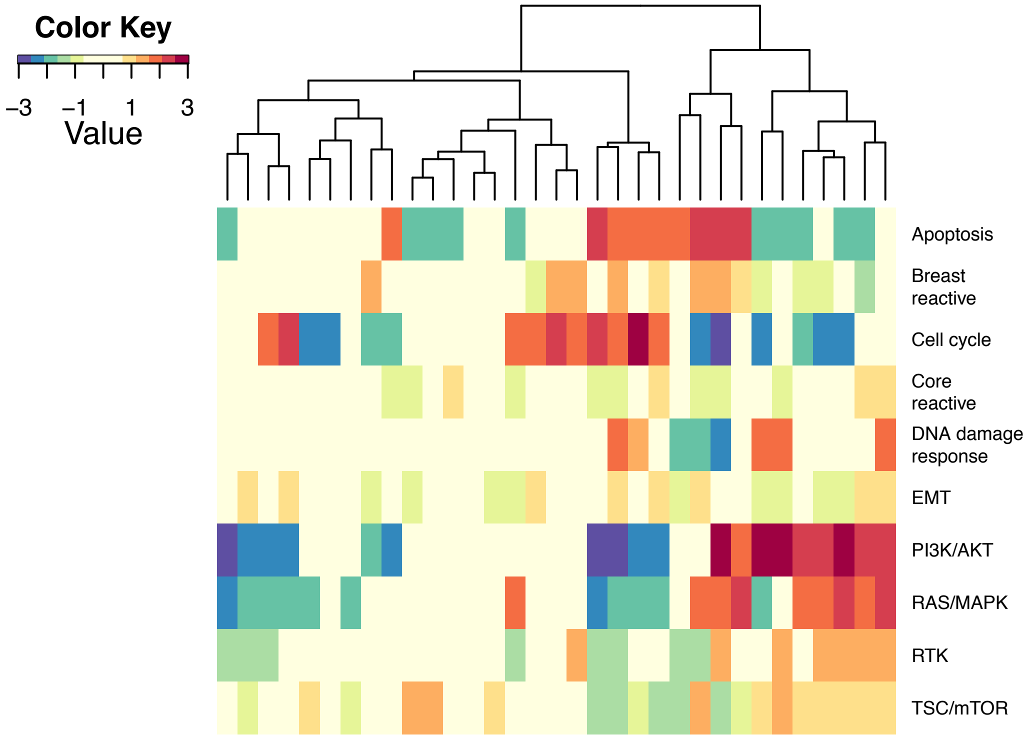

[Supplementary Figure S33] Heatmap depicting combined activated and suppressed PRECISE scores for DLBC patients. The combined scores are signed by PRECISE statuses, -1 (suppressed),1 (activated), or 0 (neutral).

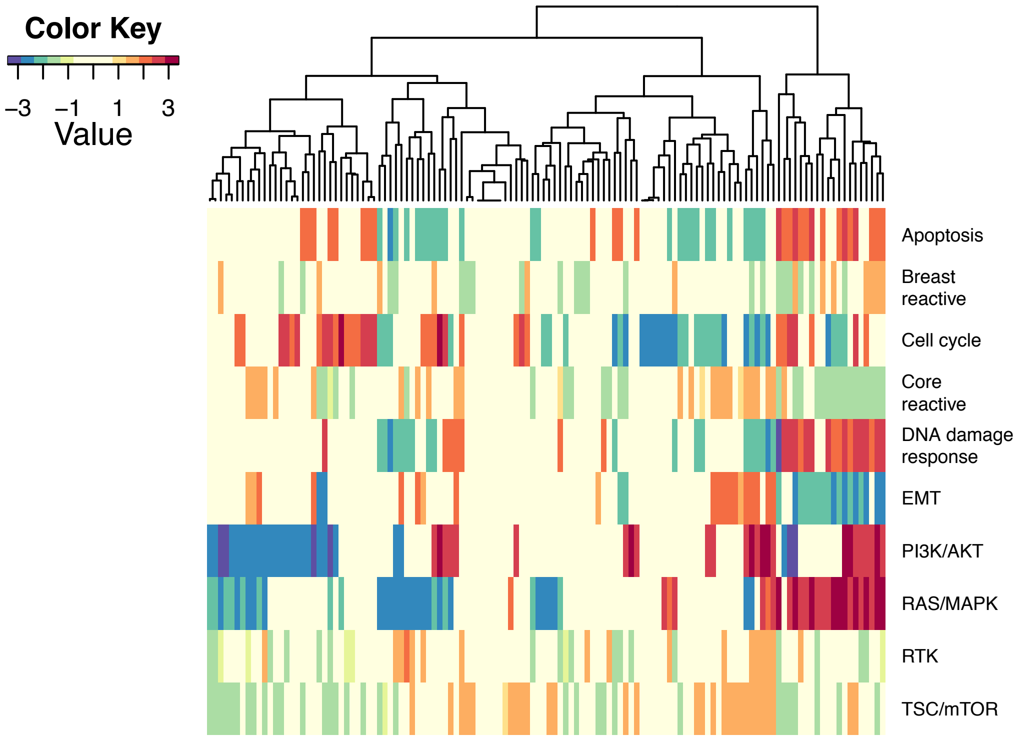

[Supplementary Figure S34] Heatmap depicting combined activated and suppressed PRECISE scores for ESCA patients. The combined scores are signed by PRECISE statuses, -1 (suppressed),1 (activated), or 0 (neutral).

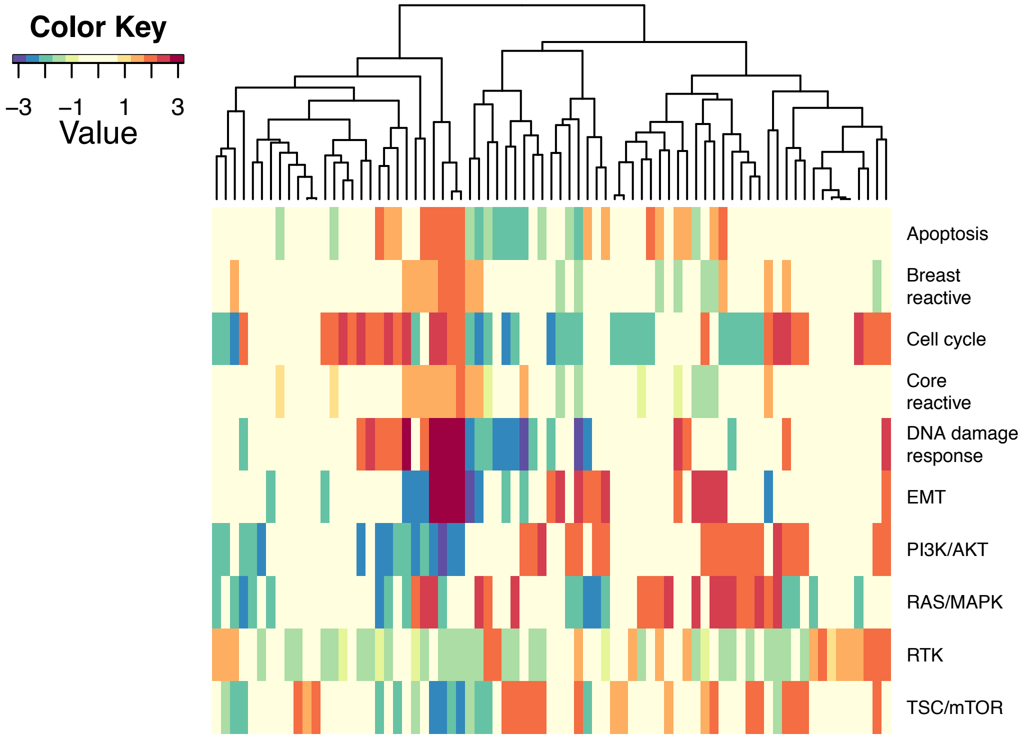

[Supplementary Figure S35] Heatmap depicting combined activated and suppressed PRECISE scores for GBM patients. The combined scores are signed by PRECISE statuses, -1 (suppressed),1 (activated), or 0 (neutral).

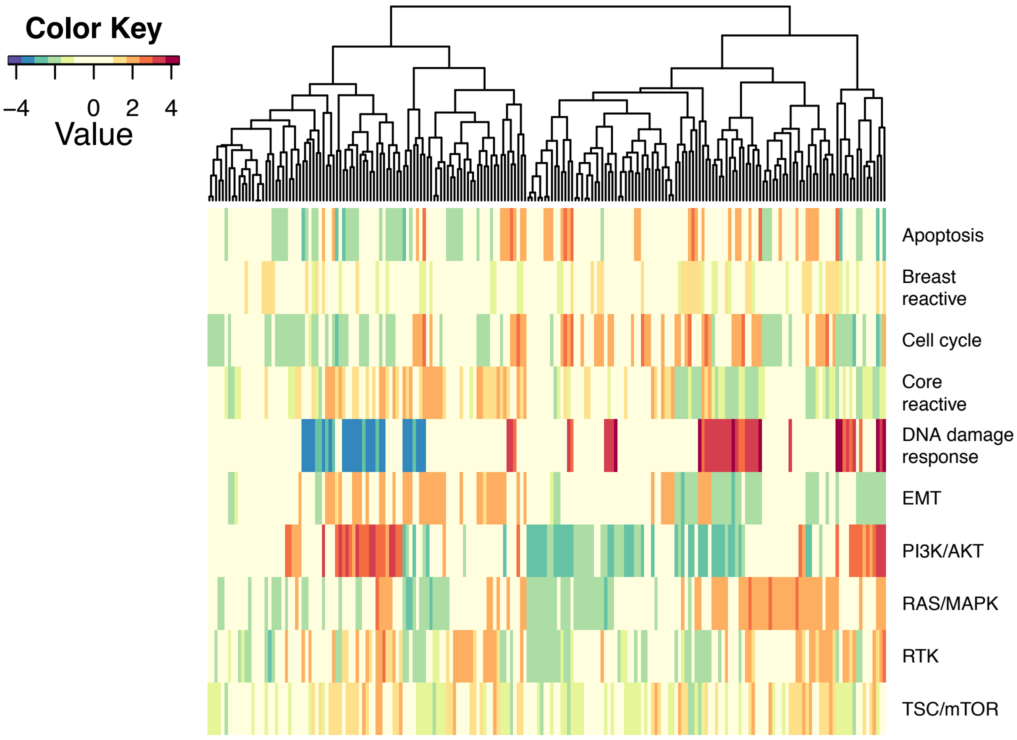

[Supplementary Figure S36] Heatmap depicting combined activated and suppressed PRECISE scores for HNSC patients. The combined scores are signed by PRECISE statuses, -1 (suppressed),1 (activated), or 0 (neutral).

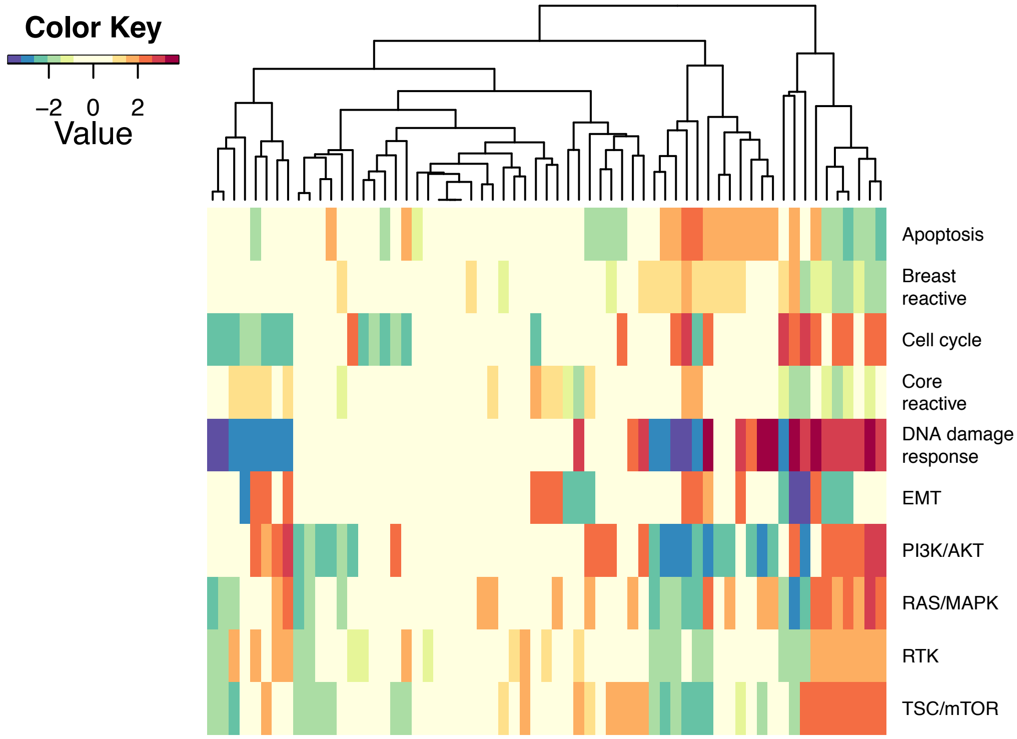

[Supplementary Figure S37] Heatmap depicting combined activated and suppressed PRECISE scores for KICH patients. The combined scores are signed by PRECISE statuses, -1 (suppressed),1 (activated), or 0 (neutral).

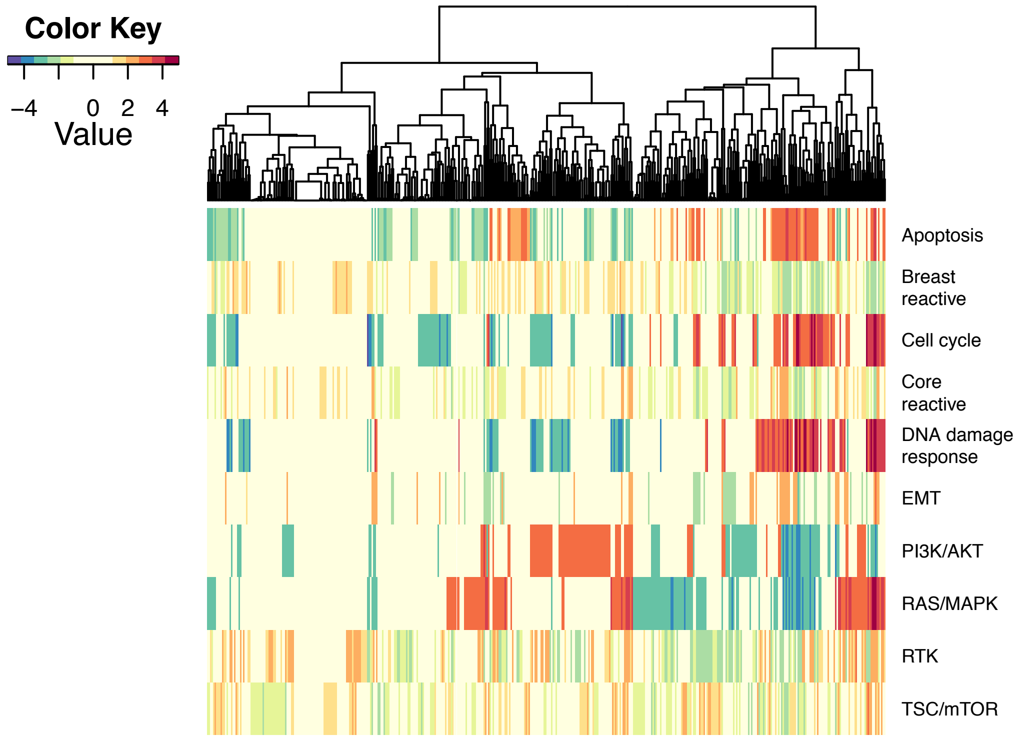

[Supplementary Figure S38] Heatmap depicting combined activated and suppressed PRECISE scores for KIRC patients. The combined scores are signed by PRECISE statuses, -1 (suppressed),1 (activated), or 0 (neutral).

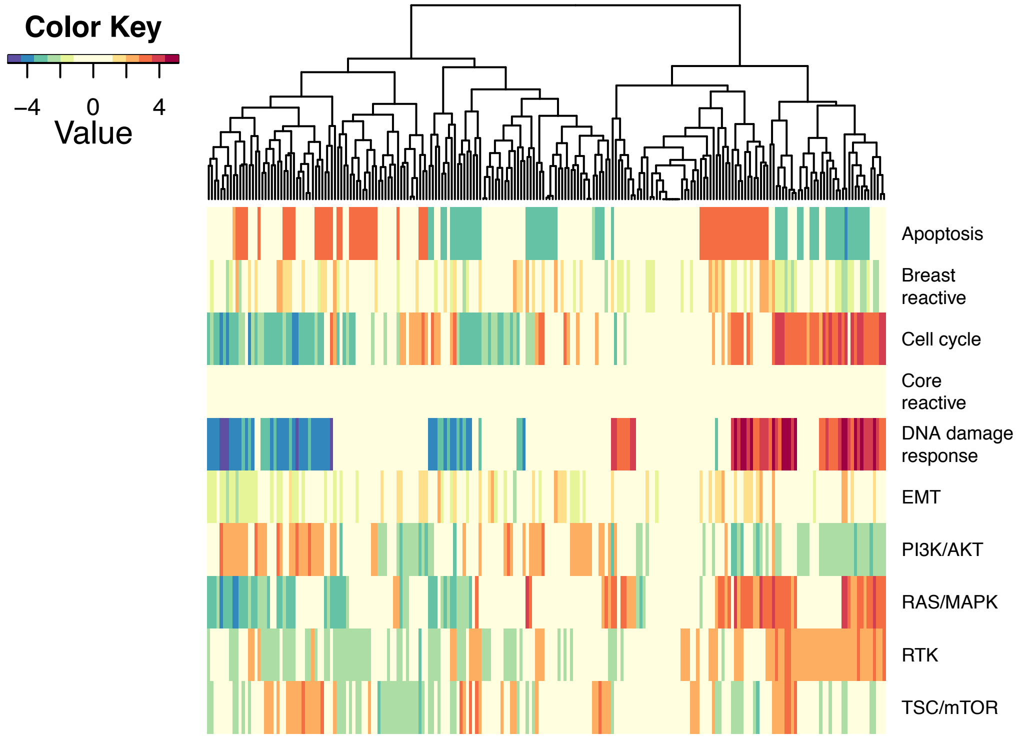

[Supplementary Figure S39] Heatmap depicting combined activated and suppressed PRECISE scores for KIRP patients. The combined scores are signed by PRECISE statuses, -1 (suppressed),1 (activated), or 0 (neutral).

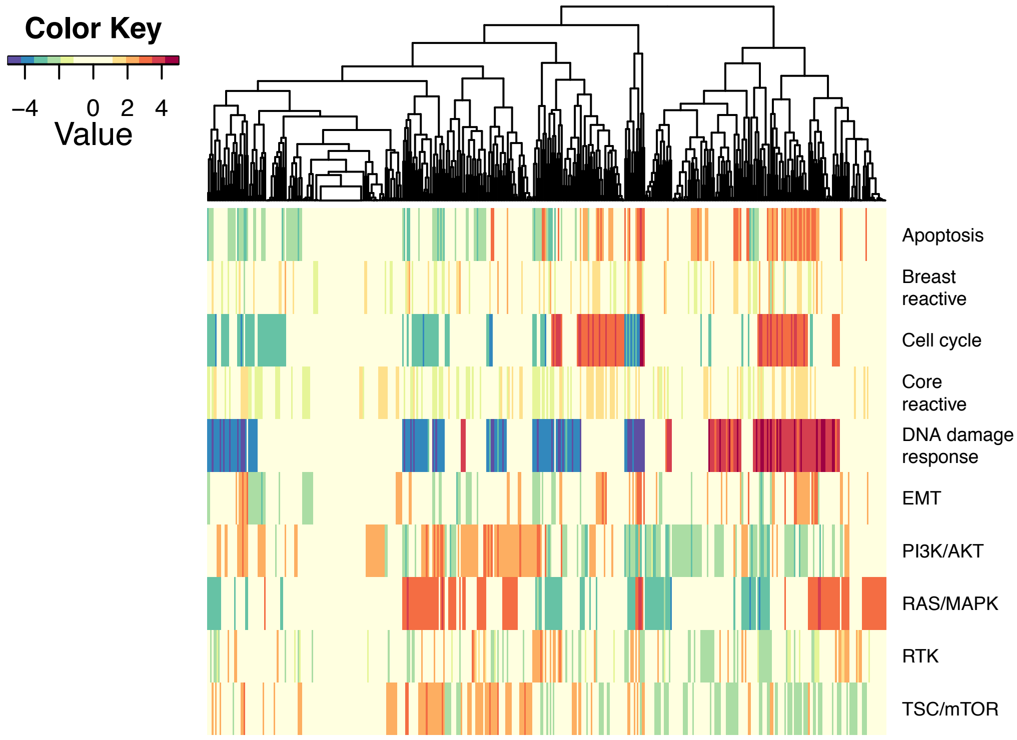

[Supplementary Figure S40] Heatmap depicting combined activated and suppressed PRECISE scores for LGG patients. The combined scores are signed by PRECISE statuses, -1 (suppressed),1 (activated), or 0 (neutral).

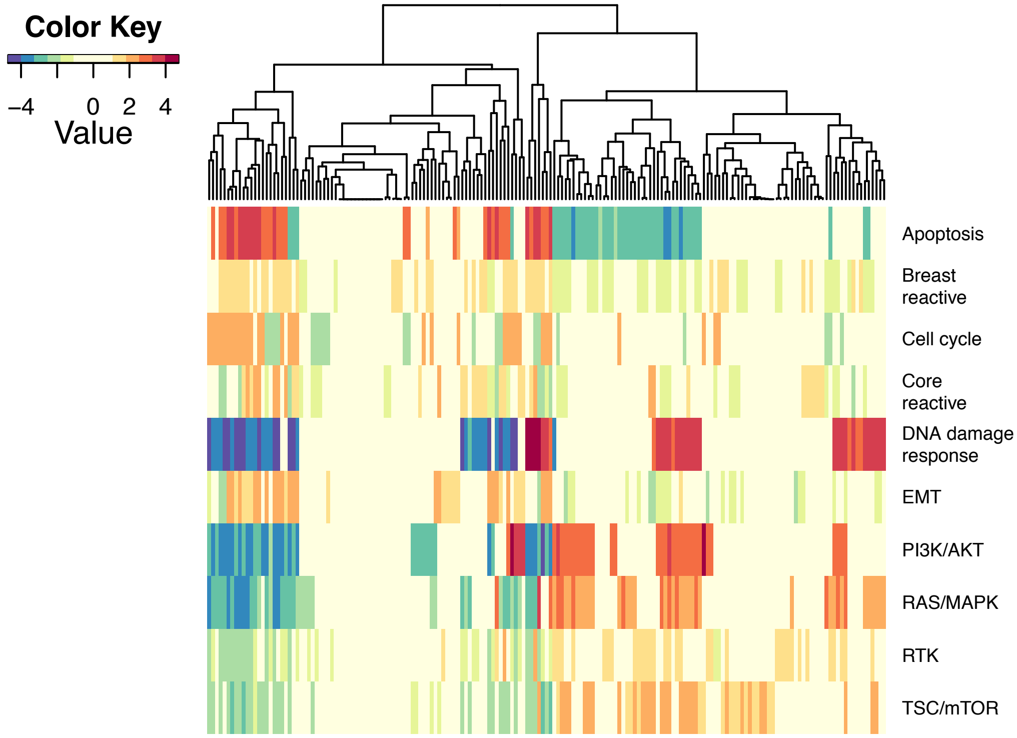

[Supplementary Figure S41] Heatmap depicting combined activated and suppressed PRECISE scores for LIHC patients. The combined scores are signed by PRECISE statuses, -1 (suppressed),1 (activated), or 0 (neutral).

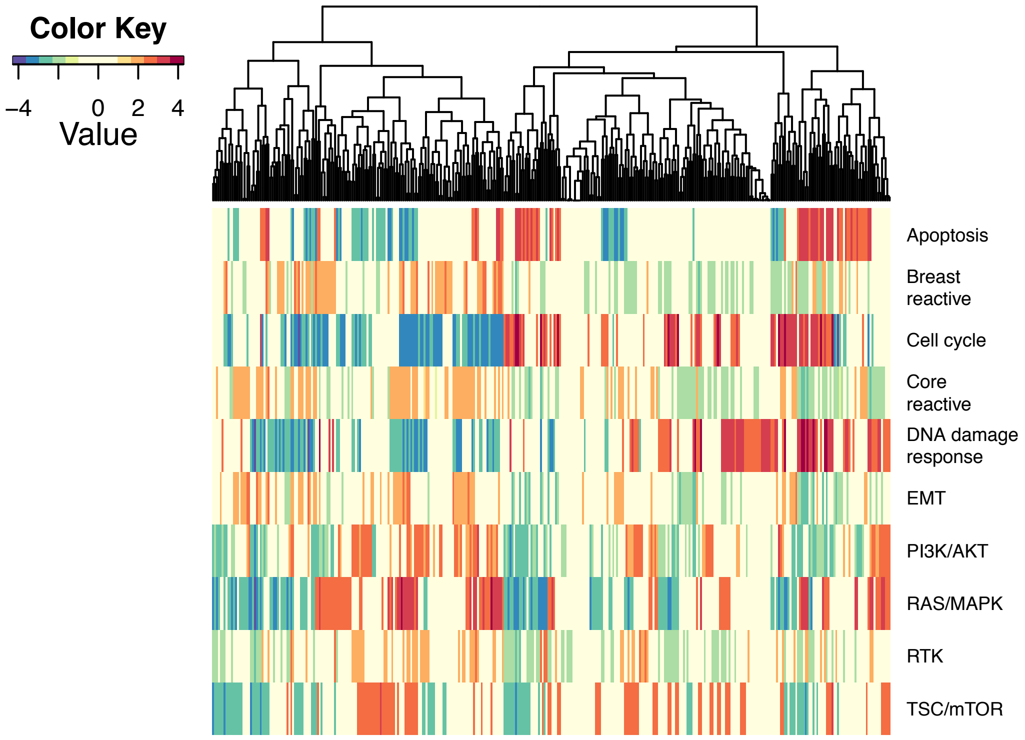

[Supplementary Figure S42] Heatmap depicting combined activated and suppressed PRECISE scores for LUAD patients. The combined scores are signed by PRECISE statuses, -1 (suppressed),1 (activated), or 0 (neutral).

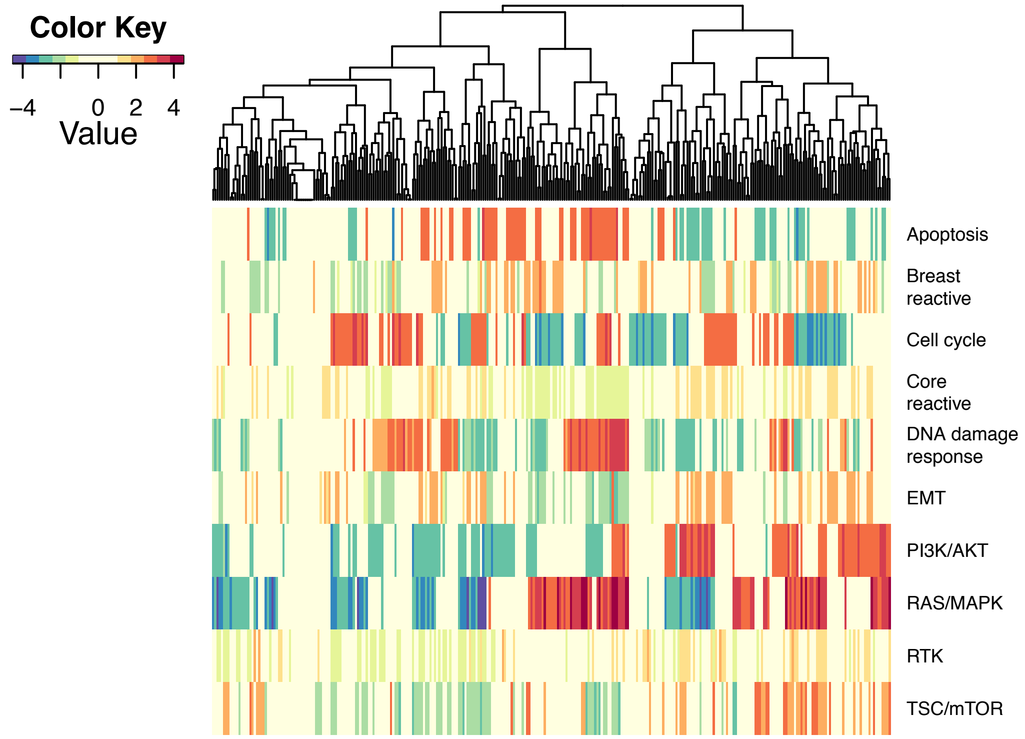

[Supplementary Figure S43] Heatmap depicting combined activated and suppressed PRECISE scores for LUSC patients. The combined scores are signed by PRECISE statuses, -1 (suppressed),1 (activated), or 0 (neutral).

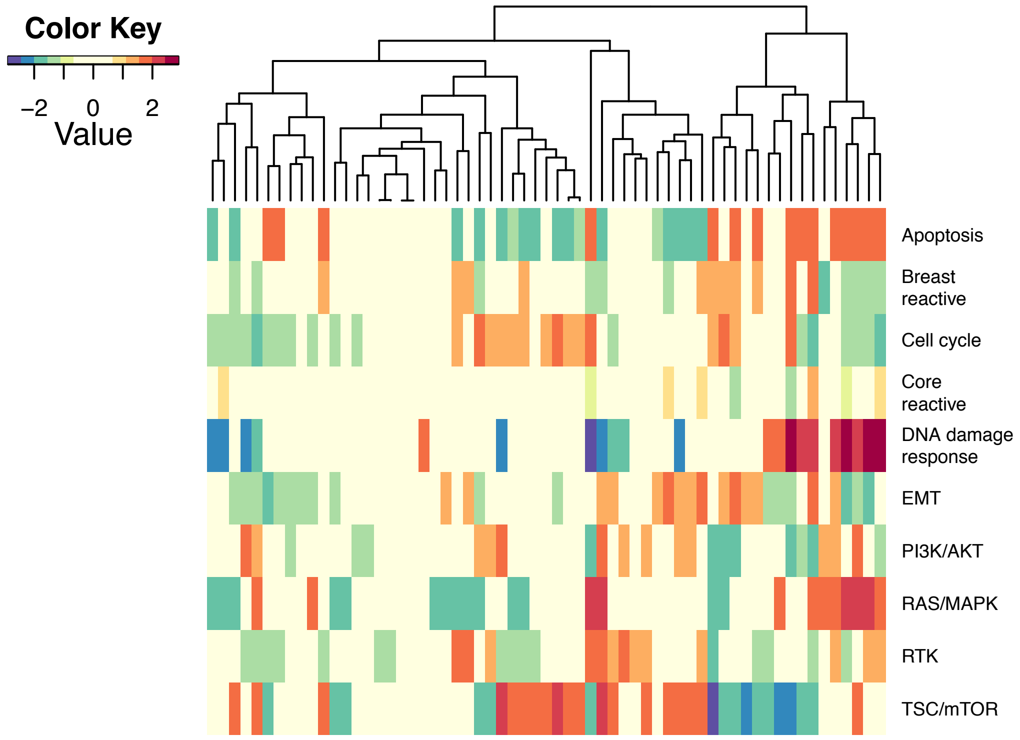

[Supplementary Figure S44] Heatmap depicting combined activated and suppressed PRECISE scores for MESO patients. The combined scores are signed by PRECISE statuses, -1 (suppressed),1 (activated), or 0 (neutral).

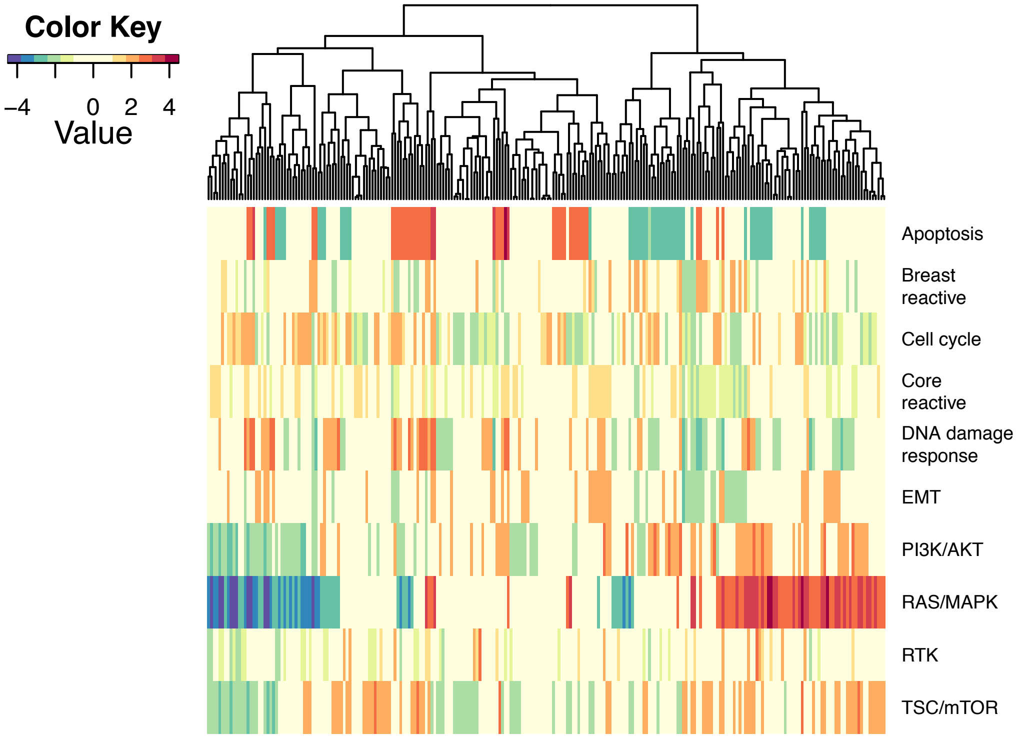

[Supplementary Figure S45] Heatmap depicting combined activated and suppressed PRECISE scores for OV patients. The combined scores are signed by PRECISE statuses, -1 (suppressed),1 (activated), or 0 (neutral).

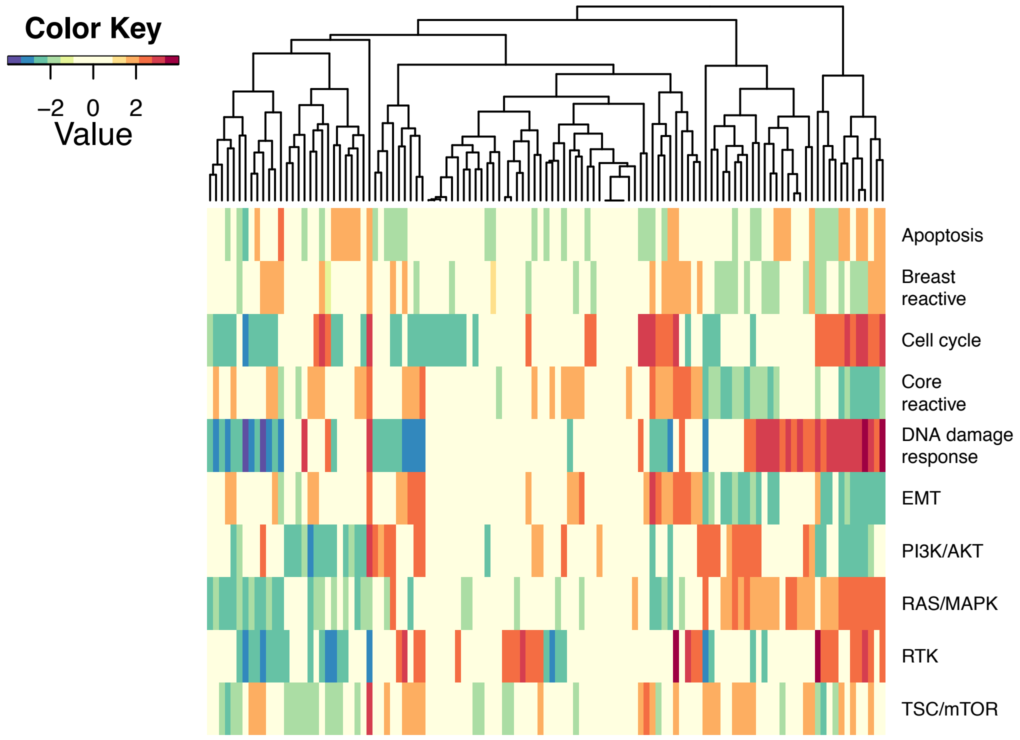

[Supplementary Figure S46] Heatmap depicting combined activated and suppressed PRECISE scores for PAAD patients. The combined scores are signed by PRECISE statuses, -1 (suppressed),1 (activated), or 0 (neutral).

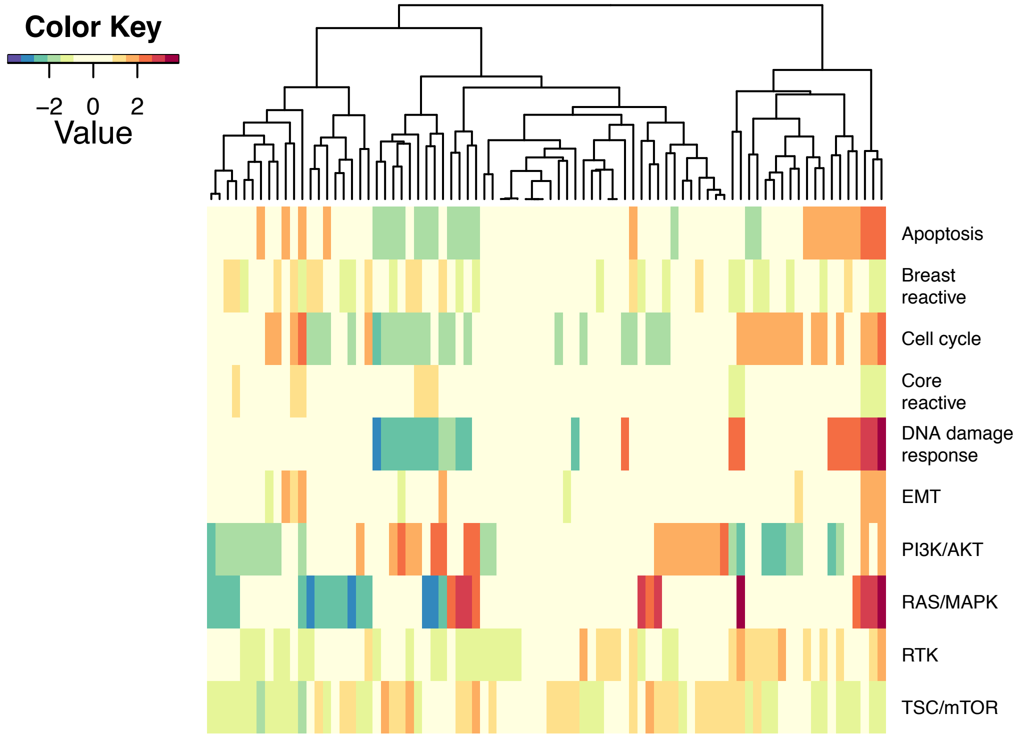

[Supplementary Figure S47] Heatmap depicting combined activated and suppressed PRECISE scores for PCPG patients. The combined scores are signed by PRECISE statuses, -1 (suppressed),1 (activated), or 0 (neutral).

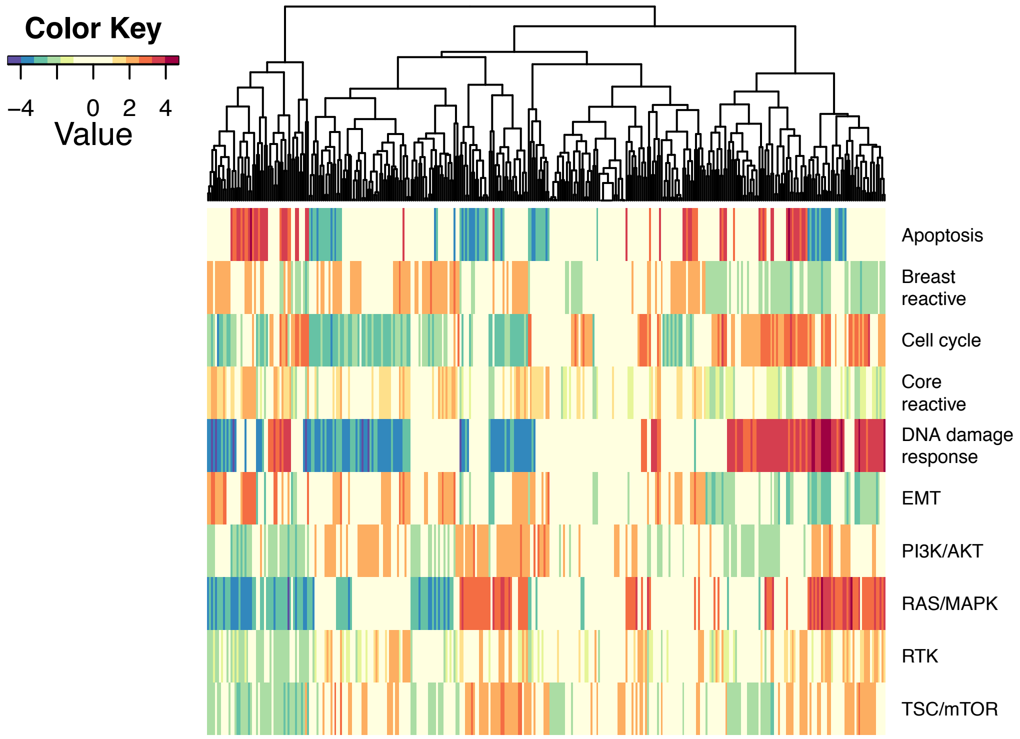

[Supplementary Figure S48] Heatmap depicting combined activated and suppressed PRECISE scores for PRAD patients. The combined scores are signed by PRECISE statuses, -1 (suppressed),1 (activated), or 0 (neutral).

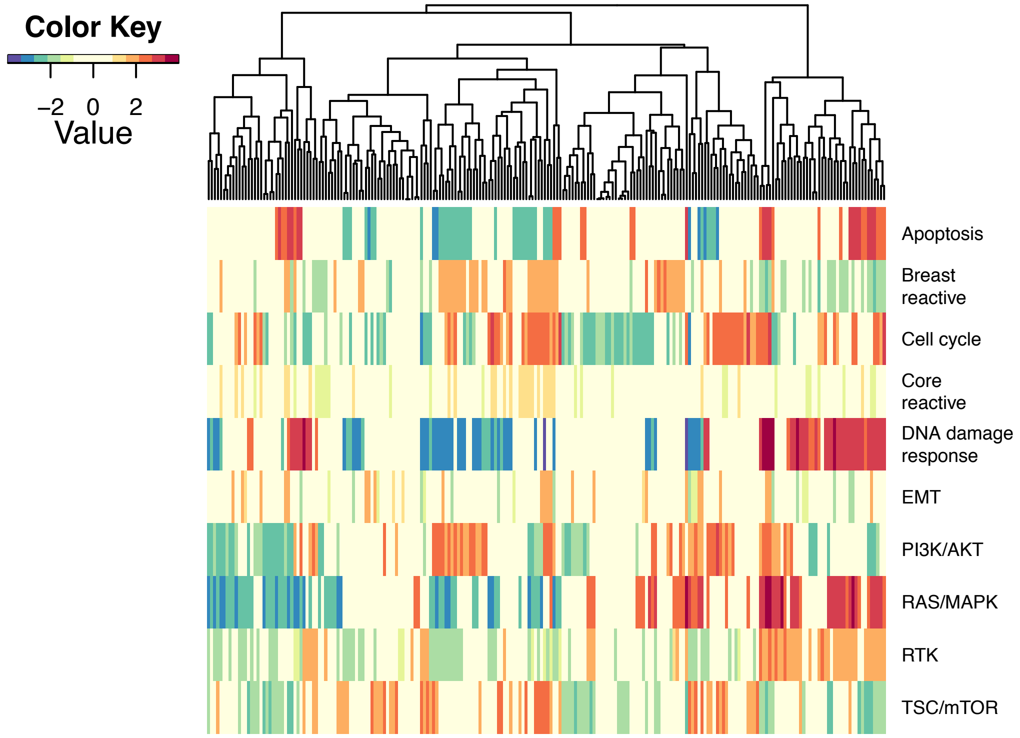

[Supplementary Figure S49] Heatmap depicting combined activated and suppressed PRECISE scores for SARC patients. The combined scores are signed by PRECISE statuses, -1 (suppressed),1 (activated), or 0 (neutral).

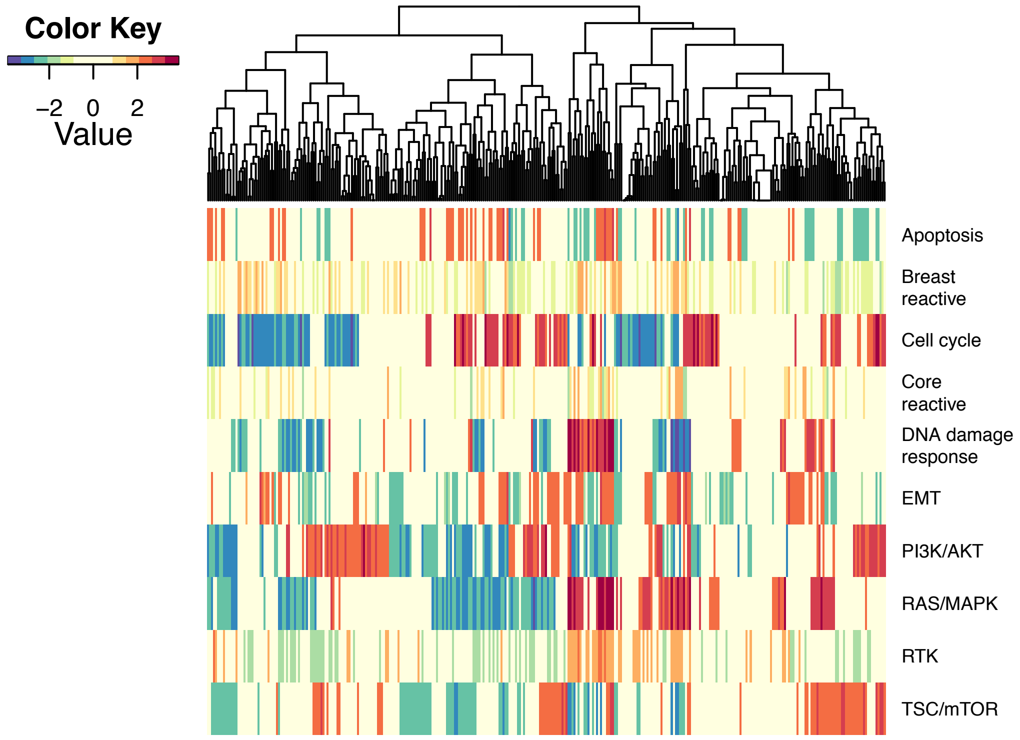

[Supplementary Figure S50] Heatmap depicting combined activated and suppressed PRECISE scores for SKCM patients. The combined scores are signed by PRECISE statuses, -1 (suppressed),1 (activated), or 0 (neutral).

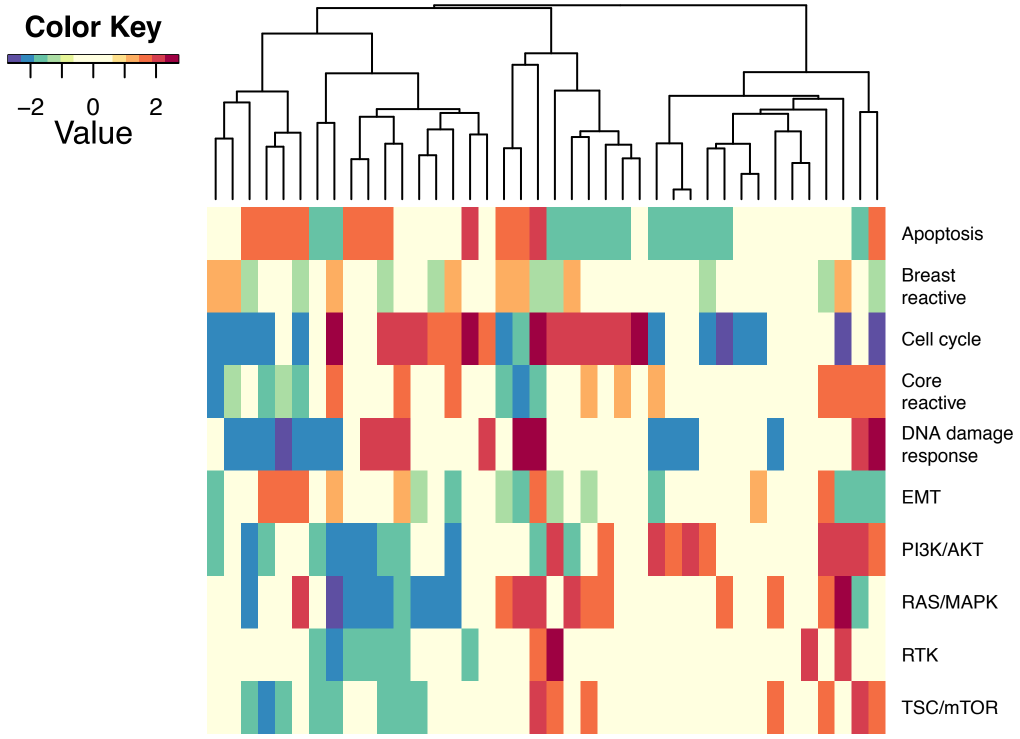

[Supplementary Figure S51] Heatmap depicting combined activated and suppressed PRECISE scores for STAD patients. The combined scores are signed by PRECISE statuses, -1 (suppressed),1 (activated), or 0 (neutral).

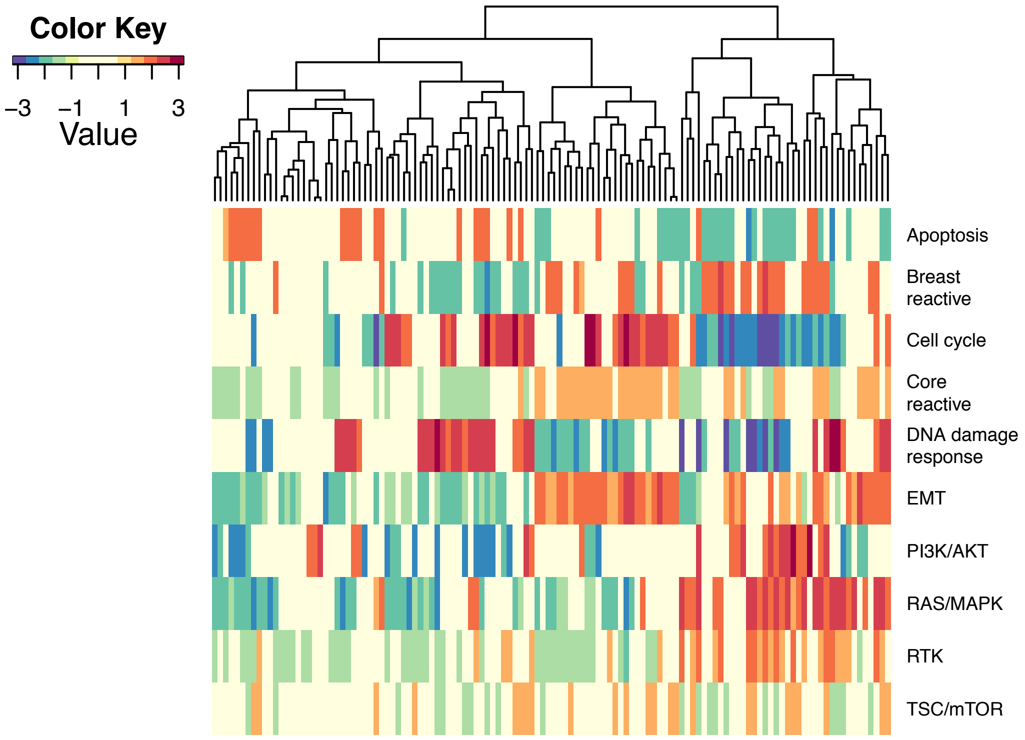

[Supplementary Figure S52] Heatmap depicting combined activated and suppressed PRECISE scores for TGCT patients. The combined scores are signed by PRECISE statuses, -1 (suppressed),1 (activated), or 0 (neutral).

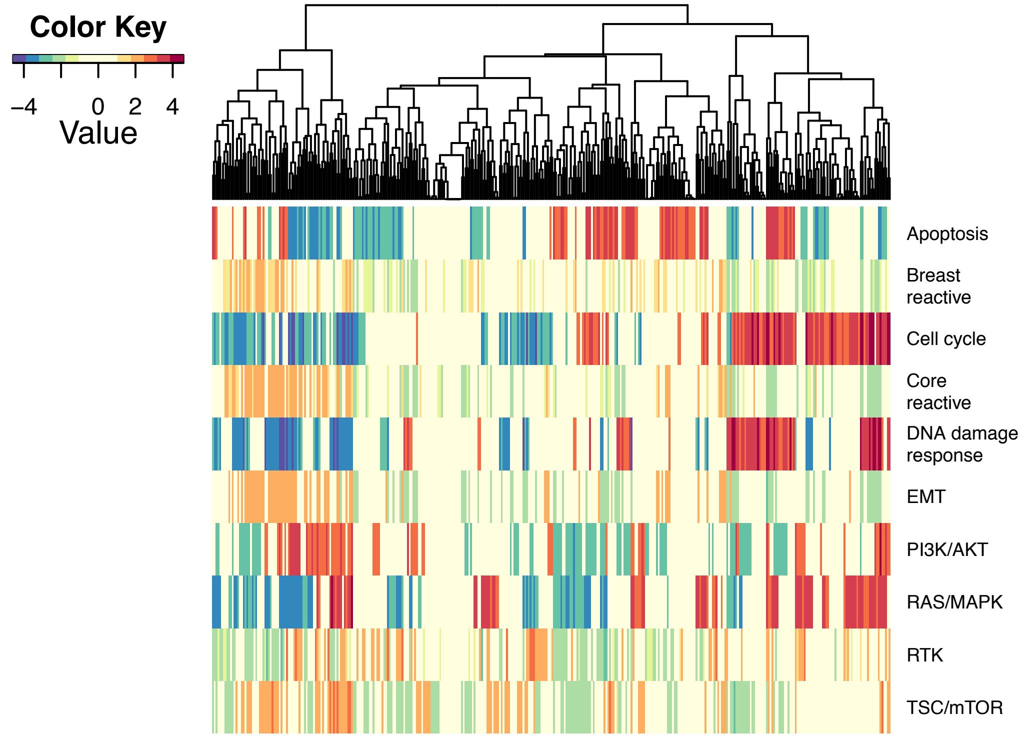

[Supplementary Figure S53] Heatmap depicting combined activated and suppressed PRECISE scores for THCA patients. The combined scores are signed by PRECISE statuses, -1 (suppressed),1 (activated), or 0 (neutral).

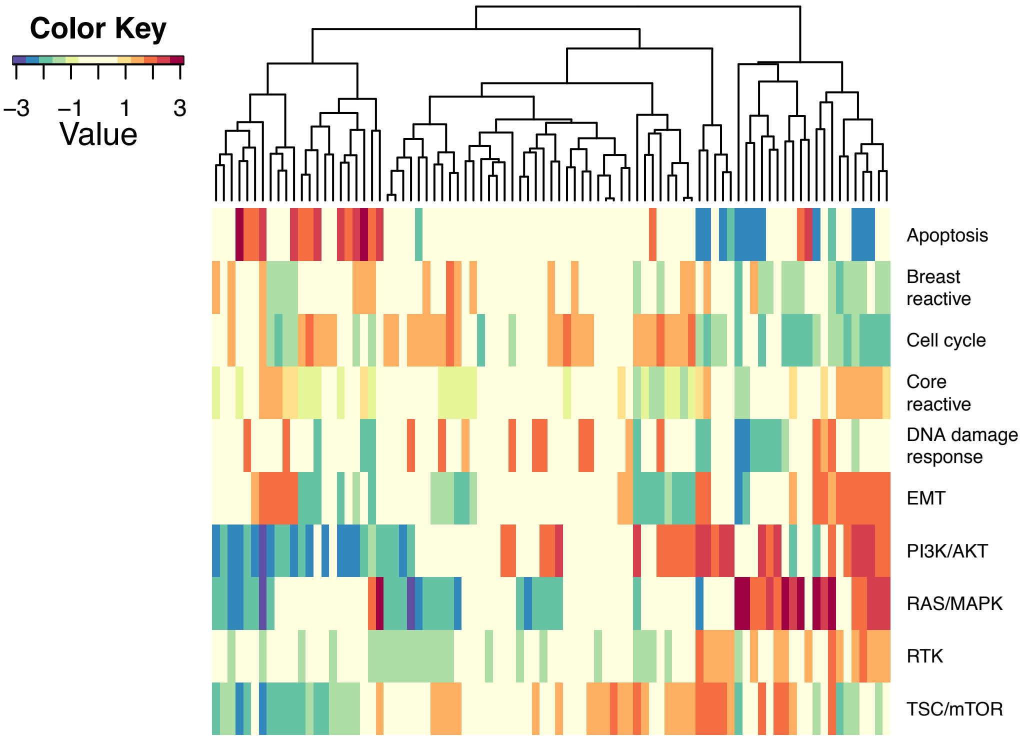

[Supplementary Figure S54] Heatmap depicting combined activated and suppressed PRECISE scores for THYM patients. The combined scores are signed by PRECISE statuses, -1 (suppressed),1 (activated), or 0 (neutral).

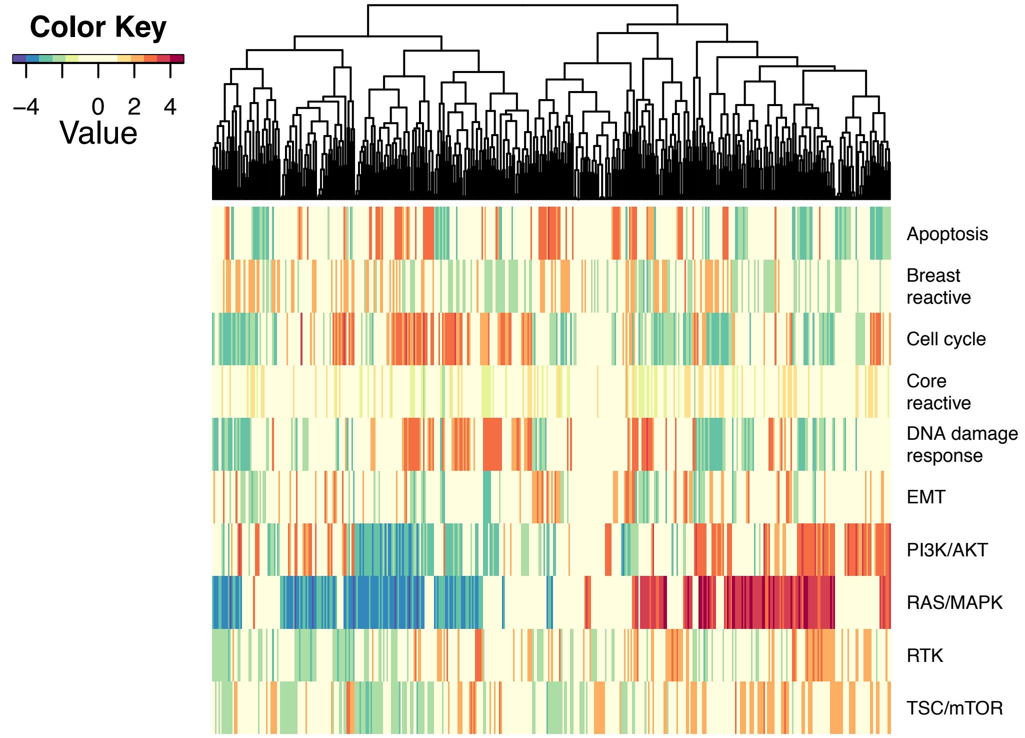

[Supplementary Figure S55] Heatmap depicting combined activated and suppressed PRECISE scores for UCEC patients. The combined scores are signed by PRECISE statuses, -1 (suppressed),1 (activated), or 0 (neutral).

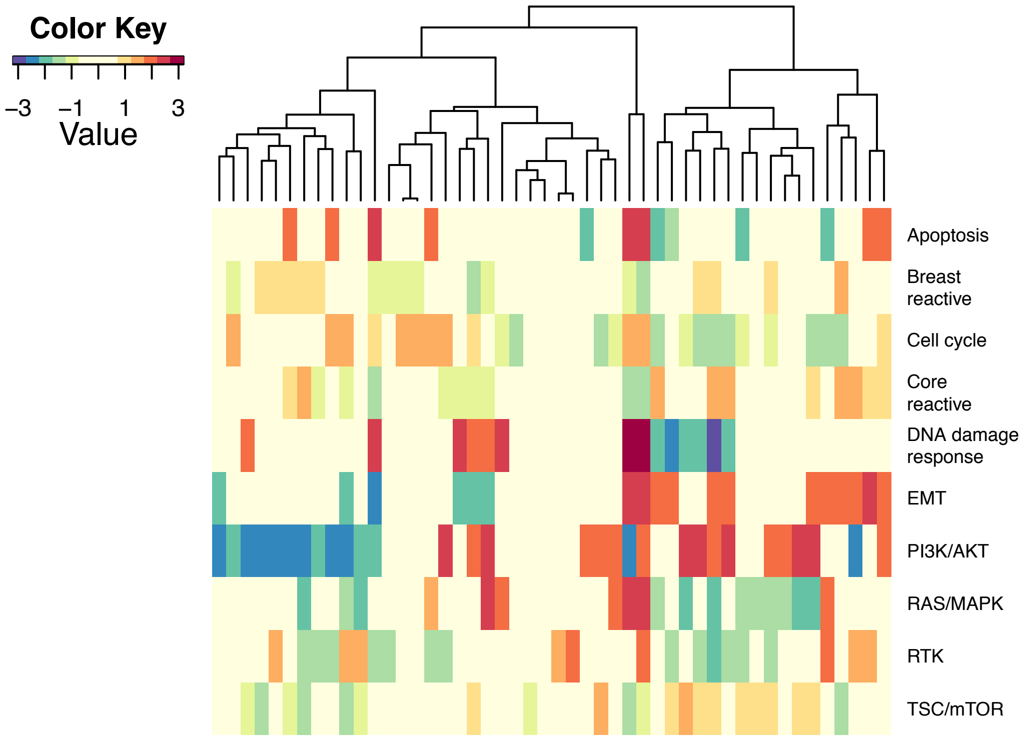

[Supplementary Figure S56] Heatmap depicting combined activated and suppressed PRECISE scores for UCS patients. The combined scores are signed by PRECISE statuses, -1 (suppressed),1 (activated), or 0 (neutral).

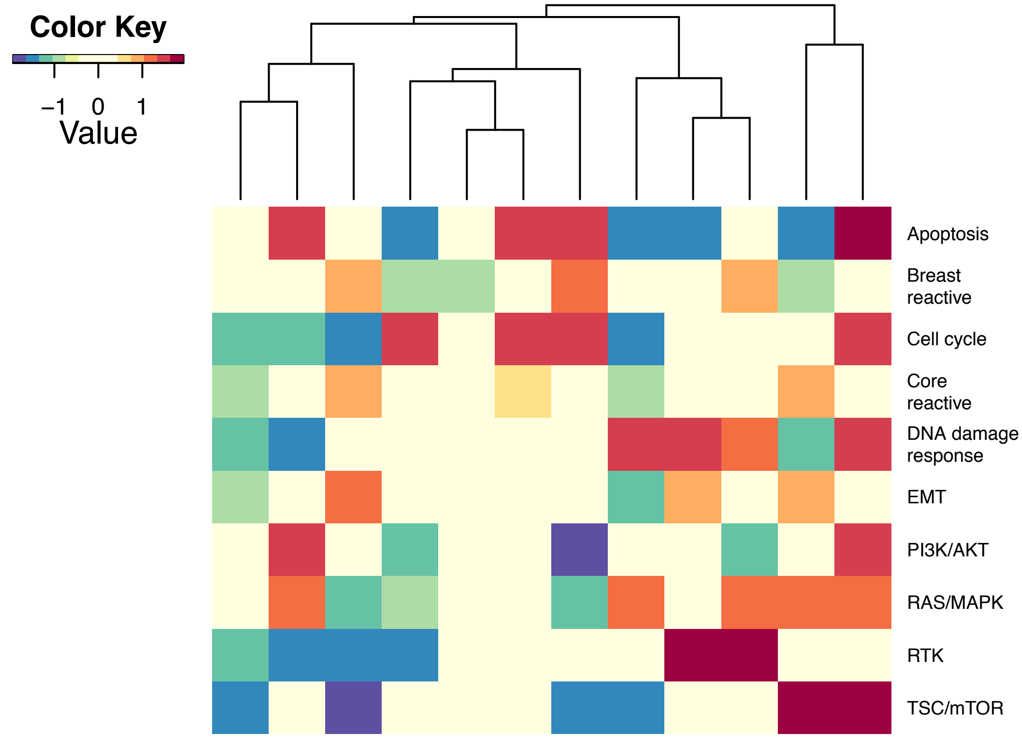

[Supplementary Figure S57] Heatmap depicting combined activated and suppressed PRECISE scores for UVM patients. The combined scores are signed by PRECISE statuses, -1 (suppressed),1 (activated), or 0 (neutral).

Supplementary Table S.1 Pathways and gene/protein names

	Pathway	Genes
1	Apoptosis	BAK1, BAX, BID, BCL2L11, CASP7, BAD, BCL2, BCL2L1, BIRC2
2	Breast reactive	CAV1, MYH11, RAB11A, RAB11B, CTNNB1, GAPDH, RBM15
3	Cell cycle	CDK1, CCNB1, CCNE1, CCNE2, CDKN1B, PCNA, FOXM1
4	Core reactive	CAV1, CTNNB1, RBM15, CDH1, CLDN7
5	DNA damage response	TP53BP1, ATM, BRCA2, CHEK1, CHEK2, XRCC5, MRE11A, TP53, RAD50, RAD51, XRCC1
6	EMT	FN1, CDH2, COL6A1, CLDN7, CDH1, CTNNB1, SERPINE1
7	PI3K/AKT	AKT1, AKT2, AKT3, GSK3A, GSK3B, CDKN1B, AKT1S1, TSC2, INPP4B, PTEN
8	RAS/MAPK	ARAF, JUN, RAF1, MAPK8, MAPK1, MAPK3, MAP2K1, MAPK14, RPS6KA1, YBX1
9	RTK	EGFR, ERBB2, ERBB3, SHC1, SRC
10	TSC/mTOR	EIF4EBP1, RPS6KB1, MTOR, RPS6, RB1
11	Hormone receptor	ESR1, PGR, AR
12	Hormone signaling (Breast)	INPP4B, GATA3, BCL2

Supplementary Table S2. Connectivity scores (permutation p-value) of the integrated cancer-specific networks. The scores that have permutation p-value<0.1 are in red color.

Cancer code	n[1]	Apoptosis	Breast reactive	Cell cycle	Core reactive	DNA damage response	EMT	PI3K/AKT	RAS/MAPK	RTK	TSC/mTOR	Hormone receptor	Hormone signaling (Breast)
KICH	63	0.28 (0.967)	0.4 (0.722)	0.57 (0.263)	0.3 (0.848)	0.45 (0.263)	0.48 (0.418)	0.48 (0.43)	0.36 (0.888)	0.6 (0.218)	0.6 (0.207)	0.67 (0.193)	0 (0.921)
KIRC	454	0.44 (0.932)	0.47 (0.762)	0.71 (0.115)	0.6 (0.362)	0.6 (0.16)	0.48 (0.739)	0.67 (0.13)	0.64 (0.088)	0.7 (0.151)	0.5 (0.639)	1 (0)	0.33 (0.929)
KIRP	215	0.53 (0.462)	0.53 (0.744)	0.62 (0.216)	0.1 (0.999)	0.53 (0.356)	0.38 (0.972)	0.48 (0.744)	0.53 (0.464)	0.7 (0.156)	0.7 (0.158)	0.67 (0.237)	0.67 (0.239)
LUAD	356	0.5 (0.554)	0.6 (0.289)	0.71 (0.085)	0.6 (0.317)	0.53 (0.47)	0.38 (0.963)	0.57 (0.478)	0.47 (0.803)	0.7 (0.131)	0.8 (0.044)	1 (0)	0 (0.939)
LUSC	309	0.44 (0.84)	0.47 (0.67)	0.67 (0.075)	0.5 (0.498)	0.42 (0.898)	0.43 (0.773)	0.57 (0.447)	0.56 (0.234)	0.5 (0.506)	0.8 (0.021)	1 (0)	0.33 (0.919)
CORE	463	0.61 (0.272)	0.67 (0.152)	0.52 (0.751)	0.8 (0.038)	0.53 (0.628)	0.62 (0.266)	0.67 (0.133)	0.53 (0.553)	0.7 (0.141)	0.8 (0.029)	1 (0)	0.33 (0.891)
PAAD	115	0.33 (0.924)	0.53 (0.355)	0.57 (0.162)	0.5 (0.29)	0.36 (0.835)	0.48 (0.306)	0.52 (0.312)	0.28 (0.965)	0.8 (0.002)	0.6 (0.107)	1 (0)	0.67 (0.076)
STAD	40	0.28 (0.753)	0.27 (0.669)	0.52 (0.099)	0.4 (0.293)	0.33 (0.57)	0.24 (0.775)	0.38 (0.455)	0.33 (0.618)	0.5 (0.122)	0.7 (0.009)	0.67 (0.05)	0 (0.714)
CHOL	30	0.5 (0.241)	0.4 (0.621)	0.43 (0.552)	0.4 (0.611)	0.27 (0.99)	0.33 (0.942)	0.48 (0.367)	0.36 (0.891)	0.6 (0.186)	0.5 (0.367)	0.67 (0.132)	0 (0.885)
LIHC	177	0.5 (0.456)	0.2 (0.998)	0.48 (0.673)	0.4 (0.83)	0.47 (0.553)	0.38 (0.968)	0.67 (0.088)	0.47 (0.728)	0.6 (0.387)	0.6 (0.331)	1 (0)	0.33 (0.96)
MESO	61	0.28 (0.675)	0.33 (0.622)	0.19 (0.957)	0.3 (0.488)	0.25 (0.864)	0.24 (0.769)	0.33 (0.612)	0.28 (0.666)	0.4 (0.239)	0.5 (0.107)	1 (0)	0 (0.747)
BLCA	337	0.39 (0.964)	0.6 (0.281)	0.76 (0.033)	0.6 (0.305)	0.51 (0.622)	0.38 (0.984)	0.67 (0.093)	0.53 (0.429)	0.5 (0.566)	0.5 (0.556)	0.67 (0.21)	0.67 (0.197)
BRCA	850	0.56 (0.807)	0.8 (0.062)	0.62 (0.538)	0.8 (0.111)	0.64 (0.252)	0.57 (0.869)	0.71 (0.335)	0.69 (0.256)	0.9 (0.007)	0.9 (0.018)	0.67 (0.314)	0.33 (0.964)
UCEC	428	0.44 (0.898)	0.53 (0.691)	0.57 (0.547)	0.6 (0.287)	0.44 (0.91)	0.52 (0.649)	0.52 (0.69)	0.69 (0.04)	0.7 (0.126)	0.7 (0.119)	0.67 (0.188)	0 (0.913)
UCS	48	0.36 (0.547)	0.13 (0.996)	0.19 (0.988)	0.4 (0.329)	0.35 (0.399)	0.48 (0.117)	0.48 (0.114)	0.31 (0.676)	0.5 (0.154)	0.2 (0.824)	0.67 (0.076)	0 (0.795)
OV	240	0.5 (0.103)	0.53 (0.268)	0.33 (0.871)	0.4 (0.458)	0.38 (0.57)	0.52 (0.222)	0.43 (0.402)	0.61 (0.004)	0.6 (0.075)	0.6 (0.079)	0.67 (0.077)	0.33 (0.829)
CESC	169	0.42 (0.475)	0.47 (0.423)	0.52 (0.373)	0.6 (0.137)	0.36 (0.819)	0.43 (0.525)	0.52 (0.376)	0.39 (0.65)	0.5 (0.327)	0.4 (0.614)	0.67 (0.132)	0.67 (0.112)
THCA	376	0.53 (0.65)	0.6 (0.395)	0.71 (0.173)	0.7 (0.156)	0.44 (0.95)	0.52 (0.806)	0.67 (0.153)	0.64 (0.143)	0.6 (0.382)	0.6 (0.382)	0.67 (0.229)	0.67 (0.265)
TGCT	122	0.33 (0.895)	0.53 (0.36)	0.57 (0.145)	0.5 (0.304)	0.4 (0.425)	0.38 (0.831)	0.48 (0.273)	0.28 (0.949)	0.3 (0.808)	0.4 (0.567)	0.67 (0.112)	0.33 (0.878)
PRAD	347	0.56 (0.553)	0.73 (0.267)	0.52 (0.864)	0.5 (0.728)	0.44 (0.959)	0.52 (0.852)	0.52 (0.865)	0.53 (0.739)	0.7 (0.208)	0.7 (0.232)	0.67 (0.26)	0.67 (0.281)
ACC	46	0.25 (0.879)	0.2 (0.884)	0.57 (0.049)	0.2 (0.806)	0.35 (0.478)	0.33 (0.698)	0.43 (0.216)	0.28 (0.795)	0.4 (0.336)	0.4 (0.332)	0.33 (0.759)	0.67 (0.063)
GBM	75	0.28 (0.866)	0.4 (0.435)	0.48 (0.136)	0.4 (0.364)	0.31 (0.697)	0.48 (0.148)	0.48 (0.153)	0.36 (0.632)	0.4 (0.465)	0.5 (0.176)	0.33 (0.855)	0.33 (0.838)
LGG	428	0.53 (0.852)	0.53 (0.896)	0.76 (0.186)	0.3 (0.986)	0.6 (0.46)	0.62 (0.488)	0.52 (0.897)	0.56 (0.76)	0.7 (0.29)	0.7 (0.277)	1 (0)	0.33 (0.947)
PCPG	82	0.31 (0.853)	0.2 (0.961)	0.33 (0.883)	0.3 (0.765)	0.29 (0.932)	0.29 (0.88)	0.33 (0.893)	0.47 (0.209)	0.4 (0.509)	0.3 (0.775)	0.67 (0.117)	0 (0.866)
HNSC	202	0.44 (0.481)	0.33 (0.884)	0.38 (0.779)	0.5 (0.275)	0.45 (0.271)	0.38 (0.809)	0.67 (0.013)	0.33 (0.896)	0.6 (0.106)	0.4 (0.537)	0.67 (0.104)	0.33 (0.85)
ESCA	124	0.31 (0.905)	0.4 (0.567)	0.52 (0.261)	0.5 (0.295)	0.31 (0.901)	0.38 (0.809)	0.62 (0.025)	0.39 (0.582)	0.4 (0.587)	0.4 (0.556)	0.67 (0.099)	1 (0)
UVM	12	0.28 (0.736)	0.13 (0.989)	0.24 (0.831)	0.1 (0.924)	0.18 (0.977)	0.1 (0.991)	0.29 (0.676)	0.17 (0.976)	0.5 (0.165)	0.5 (0.143)	0 (0.687)	0 (0.702)
SKCM	335	0.39 (0.919)	0.33 (0.98)	0.67 (0.063)	0.6 (0.254)	0.44 (0.82)	0.52 (0.629)	0.62 (0.142)	0.58 (0.206)	0.6 (0.244)	0.8 (0.027)	0.67 (0.187)	0 (0.926)
SARC	220	0.5 (0.212)	0.6 (0.152)	0.48 (0.432)	0.4 (0.677)	0.42 (0.508)	0.38 (0.903)	0.48 (0.467)	0.47 (0.486)	0.4 (0.683)	0.6 (0.199)	0.67 (0.155)	0 (0.906)
DLBC	33	0.39 (0.42)	0.27 (0.67)	0.43 (0.226)	0.1 (0.908)	0.33 (0.761)	0.1 (0.986)	0.52 (0.143)	0.42 (0.283)	0.4 (0.268)	0.2 (0.73)	0.67 (0.051)	0 (0.67)
THYM	87	0.42 (0.374)	0.4 (0.539)	0.29 (0.894)	0.2 (0.931)	0.31 (0.928)	0.33 (0.894)	0.43 (0.396)	0.33 (0.906)	0.5 (0.246)	0.5 (0.255)	0.67 (0.102)	0.33 (0.853)

[1] Sample size after matching RPPA, DNA methylation, mRNA expression, and microRNA expression data.

Supplementary Table S3. Major findings for pan-cancer shared connectivity across lineages

	Pathway	Directed (->) or undirected (-) Edges with EC>20 (number of lineages)
PPI score >0.5	Apoptosis	BCL2-BCL2L11 (27), BAD-BID (23), BAK2-BID (22)
	Cell cycle	**CCNB1 -> CCNE1 (19)**, CCNB1-FOXM1 (26), CCNB1-PCNA (26)
	Core reactive	CDH1-CTNNB1 (28), CDH1-CLDN7 (24)
	DNA damage response	RAD51-MRE11A (30), TP53BP1-XRCC5 (28), MRE11A-CHEK2 (26), XRCC5-ATM (25)
	EMT	SERPINE1-FN1 (28), CDH1-CTNNB1 (27), CDH1-CLDN7 (25)
	PI3K/AKT	AKT1/AKT2/AKT3 - GSK3A/GSK3B (29), GSK3A/GSK3B –TSC2(24) **, AKT1S1 -> AKT1/AKT2/AKT3 (19)**
	RAS/MAPK	**MAPK8 -> JUN (22)**, MAPK2K1-RAF1(28)
	RTK	**ERBB2 -> EGFR (22)**
	TSC/MTOR	MTOR-RPS6 (28), EIF4EBP1-RPS6 (23)
	Hormone receptor	AR-ESR1 (28), **PGR->ESR1 (20)**
New findings (PPI score<=0.5)	Breast reactive	CAV1-MYH11(22), CTNNB1-RBM15(22), RAB11A/RAB11B-RBM15(21)
	PI3K/AKT	GSK3A/GSK3B-AKT1S1 (26), GSK3A/GSK3B-CDKN1B(23)
	TSC/MTOR	EIF4EBP1-RB1 (24)
	Hormone signaling	BCL2-GATA3 (22)

Supplementary Table S4. The 31 pathological disease types in rows, and their relationship to the 23 subtypes defined by clustering the activated and suppressed PRECISE network score matrix.

Cancer code	C1	C2	C3	C4	C5	C6	C7	C8	C9	C10	C11	C12	C13	C14	C15	C16	C17	C18	C19	C20	C21	C22	C23	Total
KICH	0	0	0	0	0	0	0	0	0	0	0	0	3	0	31	0	24	0	5	0	0	0	0	63
KIRC	0	1	0	0	51	1	0	383	14	4	0	0	0	0	0	0	0	0	0	0	0	0	0	454
KIRP	0	0	0	0	0	0	0	0	61	0	0	0	0	0	0	0	0	0	0	24	130	0	0	215
LUAD	1	0	0	31	31	1	290	0	0	2	0	0	0	0	0	0	0	0	0	0	0	0	0	356
LUSC	0	0	0	9	19	269	0	0	0	0	10	0	0	0	0	0	0	0	0	2	0	0	0	309
CORE	0	0	0	452	6	3	1	1	0	0	0	0	0	0	0	0	0	0	0	0	0	0	0	463
PAAD	0	0	0	0	0	0	0	0	0	0	0	0	0	0	0	0	0	115	0	0	0	0	0	115
STAD	0	0	0	0	0	0	0	0	0	0	0	0	1	0	9	28	1	1	0	0	0	0	0	40
CHOL	0	0	0	0	0	0	0	0	0	0	0	0	0	0	1	29	0	0	0	0	0	0	0	30
LIHC	0	0	0	1	0	0	0	0	0	0	0	0	0	0	0	0	73	0	0	0	0	103	0	177
MESO	0	0	0	0	0	0	0	0	0	0	0	0	0	0	2	57	0	0	0	2	0	0	0	61
BLCA	0	0	0	0	14	182	0	1	0	0	2	0	0	0	0	0	0	1	137	0	0	0	0	337
BRCA	359	423	68	0	0	0	0	0	0	0	0	0	0	0	0	0	0	0	0	0	0	0	0	850
UCEC	0	0	0	0	0	0	0	0	0	424	3	1	0	0	0	0	0	0	0	0	0	0	0	428
UCS	0	0	0	0	0	0	0	0	0	0	0	0	0	0	0	44	4	0	0	0	0	0	0	48
OV	0	0	0	0	0	4	0	0	0	8	0	6	0	222	0	0	0	0	0	0	0	0	0	240
CESC	0	0	0	0	0	3	0	0	0	0	1	0	0	0	114	3	1	2	44	0	0	0	1	169
THCA	2	0	0	3	106	3	0	256	3	2	0	0	0	0	0	0	0	0	1	0	0	0	0	376
TGCT	0	0	0	0	0	0	0	0	0	0	0	0	0	0	118	2	1	0	1	0	0	0	0	122
PRAD	2	0	0	0	0	0	0	0	0	20	0	324	0	1	0	0	0	0	0	0	0	0	0	347
ACC	0	0	0	0	0	0	0	0	0	0	0	0	0	0	24	20	1	0	0	0	0	0	1	46
GBM	0	0	0	0	0	0	0	0	0	0	0	0	0	0	68	0	2	0	5	0	0	0	0	75
LGG	1	0	0	0	2	6	1	0	145	2	269	0	2	0	0	0	0	0	0	0	0	0	0	428
PCPG	0	0	0	0	0	0	0	0	0	0	0	0	0	0	0	78	0	0	0	4	0	0	0	82
HNSC	0	0	0	0	0	0	0	0	0	0	0	0	0	0	8	0	187	0	0	3	0	4	0	202
ESCA	0	0	0	0	0	0	0	0	0	0	0	0	0	0	0	0	0	0	8	0	0	0	116	124
UVM	0	0	0	0	0	0	0	0	0	0	0	0	0	0	0	12	0	0	0	0	0	0	0	12
SKCM	0	0	0	1	11	24	0	1	0	3	4	0	291	0	0		0	0	0	0	0	0	0	335
SARC	0	0	0	0	0	7	1	0	3	0	2	1	0	1	0	0	0	0	0	205	0	0	0	220
DLBC	0	0	0	0	0	0	0	0	0	0	0	0	0	0	0	33	0	0	0	0	0	0	0	33
THYM	0	0	0	0	0	0	0	0	0	0	0	0	0	4	0	83	0	0	0	0	0	0	0	87
Total	365	424	68	497	240	503	293	642	226	465	291	332	297	228	375	389	294	119	201	240	130	107	118	6844

Supplementary Table S5. The proportion of the samples with the combined suppressed and activated PRECISE score > 2.521 (the third quantile) to the number of samples in a cluster. The proportions greater than 0.5 are in red.

Cluster	Sample size	Enriched tumor type	Apoptosis	Breast reactive	Cell cycle	Core reactive	DNA damage response	EMT	PI3K/AKT	RAS/MAPK	RTK	TSC/mTOR	Hormone receptor	Hormone signaling (Breast)
C1	365	BRCA	1	0.2356	0.3425	0.0137	1	0.0137	0.9425	1	0.2027	0.9918	0	0
C2	424	BRCA	0.9976	0.4976	0.5283	0.2972	1	0.2689	0.9811	1	0.0943	1	0	0
C3	68	BRCA	1	0.9706	0.7059	0.7353	1	0.5735	0.9853	1	0.3088	1	0	0
C4	497	CORE	0.996	0	0.4165	0.1811	0.8732	0.2314	0.4326	0.8974	0.002	0.1529	0.0563	0
C5	240	THCA, KIRC	0.8792	0	0.95	0	0.9625	0.1042	0.7792	0.9958	0.0208	0.1292	0	0
C6	503	LUSC, BLCA	0.2068	0	0.5487	0	0.2843	0	0.3837	0.66	0	0.0358	0	0
C7	293	LUAD	0.8498	0.0068	0.7338	0	0.5529	0.0068	0.1365	0.4334	0	0.1945	0	0
C8	642	KIRC, THCA	0.6308	0	0.7212	0	0.8692	0.0031	0.9019	1	0.0187	0.0016	0	0
C9	226	KIRP, LGG	0.7124	0	0.9558	0	1	0.0973	0.3717	0.9027	0.0973	0.1239	0	0
C10	465	UCEC	0.3312	0.0108	0.286	0	0.3183	0.1032	0.3978	0.9763	0.0667	0.0473	0	0
C11	291	LGG	0.2818	0	0.9759	0	0.9966	0.0653	0.1478	0.8041	0	0.0103	0	0
C12	332	PRAD	0.997	0.0181	0.3675	0	0.8795	0.1355	0.0181	0.747	0	0.0392	0	0
C13	297	SKCM	0.0404	0	0.4848	0	0.2559	0.0471	0.367	0.4108	0	0.1481	0	0
C14	228	OV	0.4123	0	0	0	0.0351	0	0.0614	0.6711	0	0.0175	0	0
C15	375	[illegible]ICH, CESC, TGCT, ACC, GBM	0.0027	0	0.104	0	0.1307	0.008	0.08	0.0213	0	0	0	0
C16	389	[illegible]IOL, MESO, ACC, PCPG, THYM	0.0206	0	0.0051	0	0.018	0	0.0334	0.0694	0.0077	0.0026	0.0026	0
C17	294	KICH, LIHC, HNSC	0.0136	0	0.0204	0	0.7755	0.0204	0.2007	0.0102	0	0	0	0
C18	119	PAAD	0	0	0.1513	0	0.3782	0.0504	0.0588	0.0168	0.4286	0.0084	0	0
C19	201	BLCA, CESC	0.0697	0	0.4627	0	0.7711	0.2338	0.3831	0.6468	0	0	0	0
C20	240	KIRP, SARC	0.2583	0	0.0792	0	0.5833	0	0.0083	0.4167	0	0.0125	0	0
C21	130	KIRP	0.9692	0	0.4923	0	0.5846	0	0.2	0.3154	0.1077	0.1	0	0
C22	107	LIHC	0.785	0	0	0	0.972	0	0.8224	0.3832	0	0.0374	0	0
C23	118	ESCA	0	0	0.1102	0	0.0169	0.0085	0.3983	0.1949	0	0	0	0

Supplementary Table S6. Proportions (%) of mutations for each gene to the total number of mutations across all clusters

	C1	C2	C3	C4	C5	C6	C7	C8	C9	C10	C11	C12	C13	C14	C15	C16	C17	C18	C19	C20	C21	C22	C23
TP53	7.56	5.5	0.69	9.97	2.41	11.68	7.9	0.34	2.75	2.06	2.06	0	4.12	4.12	4.12	2.75	11	4.81	5.5	3.09	0	1.37	6.19
APC	0	0	0	**82.46**	1.32	3.51	0.44	0.44	0	4.82	0	0.88	1.75	0	0	0.88	0.44	0	0.88	1.32	0	0.88	0
TTN	1.04	2.08	0	11.98	2.08	12.5	4.69	4.17	3.12	11.98	1.04	1.04	19.27	2.6	3.12	1.56	4.17	2.6	5.73	2.08	0.52	0.52	2.08
ARID1A	2.05	2.74	1.37	6.85	0.68	19.86	4.79	2.05	2.05	26.71	3.42	0.68	1.37	0	2.74	3.42	4.11	2.74	8.22	0.68	0	0.68	2.74
MUC16	1.05	1.05	0	9.47	3.16	13.68	17.89	0	1.05	10.53	0	1.05	13.68	1.05	1.05	2.11	6.32	3.16	6.32	3.16	1.05	2.11	1.05
CSMD3	0	0	0	8.99	3.37	16.85	20.22	1.12	0	8.99	1.12	2.25	14.61	0	2.25	3.37	7.87	0	3.37	1.12	1.12	0	3.37
KMT2C	8.79	2.2	3.3	5.49	0	15.38	3.3	5.49	0	5.49	0	4.4	2.2	0	9.89	8.79	5.49	1.1	13.19	1.1	0	1.1	3.3
NF1	0	4.6	2.3	5.75	1.15	9.2	5.75	2.3	5.75	9.2	6.9	0	18.39	2.3	9.2	4.6	2.3	2.3	5.75	1.15	0	0	1.15
KMT2D	0	0	0	3.19	4.26	30.85	2.13	0	0	4.26	3.19	3.19	0	0	5.32	4.26	11.7	1.06	21.28	1.06	0	0	4.26
PTEN	3.66	2.44	0	6.1	3.66	6.1	1.22	2.44	0	47.56	1.22	0	6.1	0	4.88	3.66	2.44	0	6.1	0	0	1.22	1.22
FAT1	2.99	0	0	0	0	23.88	1.49	0	0	22.39	0	0	0	0	2.99	0	34.33	2.99	7.46	1.49	0	0	0
ATRX	0	0	0	1.43	2.86	2.86	5.71	0	18.57	10	35.71	0	1.43	0	4.29	1.43	0	1.43	1.43	11.43	0	1.43	0
RB1	6.15	4.62	0	3.08	1.54	30.77	7.69	0	0	7.69	0	0	6.15	1.54	4.62	1.54	1.54	0	16.92	4.62	0	0	1.54
SYNE1	0	4.84	0	9.68	3.23	20.97	6.45	1.61	1.61	11.29	0	1.61	11.29	1.61	6.45	1.61	6.45	0	8.06	1.61	0	0	1.61
PBRM1	0	0	0	4.48	1.49	2.99	0	**59.7**	2.99	7.46	0	0	7.46	1.49	0	5.97	1.49	0	2.99	0	1.49	0	0
DNAH5	0	3.33	0	8.33	0	10	5	0	0	13.33	0	0	31.67	1.67	1.67	3.33	8.33	0	3.33	6.67	0	0	3.33
RYR2	3.7	0	0	11.11	1.85	18.52	20.37	0	0	14.81	0	0	9.26	0	1.85	0	3.7	1.85	5.56	1.85	0	0	5.56
CDKN2A	0	0	0	0	0	8.47	0	1.69	0	0	1.69	1.69	18.64	0	0	0	45.76	13.56	1.69	1.69	1.69	3.39	0
FAT4	1.85	1.85	1.85	16.67	1.85	14.81	3.7	0	0	22.22	0	1.85	12.96	0	7.41	3.7	0	3.7	3.7	0	0	0	1.85
LRP1B	1.85	0	0	11.11	3.7	5.56	12.96	1.85	0	12.96	3.7	0	29.63	0	1.85	1.85	5.56	3.7	0	0	0	0	3.7

Supplementary Table S7. P-values for PRECISE scores using log-rank test for survival times. P-values with FDR<0.1 are displayed in red.

Cancer	Apoptosis	Breast reactive	Cell cycle	Core reactive	DNA damage response	EMT	PI3K/AKT	RAS/MAPK	RTK	TSC/mTOR	Hormone receptor	Hormone signaling (Breast)
KICH	0.2314	0.8269	0.1425	0.0354	0.8774	0.0017	0.7846	0.3415	0.0129	0.6407	0.9783	0.4518
KIRC	0.052	0.2289	0.0108	2.00E-04	0.0249	0.0282	0.0036	0.0023	0	0.3242	0.0107	0.5959
KIRP	0.1521	0.0416	0.0616	0.5576	0.6528	0.0753	0.4411	0.1201	0.1476	0.0056	0.7284	0.7465
LUAD	0.6559	0.426	0.3732	0.7086	0.7247	0.6532	0.8184	0.7839	0.3257	0.7434	0.6985	0.617
LUSC	0.7155	0.014	0.0264	0.9094	0.0846	0.9278	0.67	0.3806	0.0072	0.0552	0.2367	0.2477
CORE	0.0683	0.9057	0.0137	0.3177	0.5142	0.8225	0.6712	0.9755	0.2737	0.2815	0.7514	0.6666
PAAD	0.3835	0.964	0.0623	0.8045	0.0927	0.1209	0.4123	0.4089	0.1807	0.1549	0.4118	0.886
STAD	0.8908	0.7339	0.5906	0.901	0.9256	0.3358	0.0546	0.2864	0.0014	0.7815	0.8814	0.8585
CHOL	0.9304	0.3312	0.9649	0.866	0.4955	0.9521	0.5874	0.7658	0.9231	0.3263	0.4257	0.2358
LIHC	0.2996	0.1273	0.3664	0.2174	0.5993	0.5857	0.2066	0.1163	0.103	0.2747	0.7015	0.0263
MESO	0.1886	0.0785	0	0.0331	0.3363	0.4698	0.6395	0.4056	0.2227	0.5781	0.9423	0.4059
BLCA	0.5588	0.4582	0.8317	0.9166	0.4377	0.5955	0.9656	0.315	0.8503	0.9127	0.0325	0.3071
BRCA	0.3723	0.0048	0.7901	0.003	0.6133	0.1962	0.0497	0.1395	0.685	0.3126	0.3248	0.3391
UCEC	0.1395	0.9202	0.9413	0.4095	0.5071	0.0462	0.5318	0.8339	0.9261	0.958	0.0045	0.3623
UCS	0.5575	0.1573	0.4309	0.5448	0.9625	0.9164	0.4289	0.7348	0.4544	0.6756	0.6732	0.3808
OV	0.2346	0.1303	0.5309	0.1228	0.8325	0.0289	0.2693	0.1218	0.1551	0.5947	0.468	0.6204
CESC	0.2867	0.9858	0.2263	0.1712	0.2005	0.4852	0.1254	0.7191	0.6462	0.8741	0.7699	0.795
THCA	0.5813	0.1983	0.3093	0.1001	0.6968	0.0182	0.7186	0.1636	0.749	0.0066	0.963	0.0717
TGCT	0.084	0.6198	0.8769	0.4875	0.9002	0.9102	0.4004	0.3698	0.8745	0.7056	0.6676	0.4013
PRAD	0.837	0.4996	0.521	0.3899	0.3556	0.2224	0.5224	0.5873	0.8712	0.8072	0.9418	0.6094
ACC	0.234	0.0393	0.0017	0.2335	0.2138	0.0062	0.2238	0.3823	0.7832	0.2685	0.3451	0.7815
GBM	0.3173	0.9952	0.4727	0.8009	0.4452	0.3696	0.2673	0.6373	0.9712	0.0364	0.0659	0.5608
LGG	0.08	0.72	0.1453	0.6379	0	0.0166	0.464	0.0226	0.0044	0.6219	6.00E-04	1.00E-04
PCPG	5.00E-04	0.4524	0.2856	0.0352	0.2569	0.869	0.5205	0.5994	0.5373	0.2339	0.6189	0.2681
HNSC	0.919	0.2822	0.9191	0.6057	0.0388	0.5106	0.3167	0.2119	0.7566	0.5758	0.459	0.1059
ESCA	0.4792	0.9405	0.8352	0.12	0.3824	0.4761	0.0811	0.2893	0.3769	0.4289	0.041	0.7565
UVM	0.5809	0.6186	0.5809	0.5649	0.6989	0.4617	0.6707	0.6705	0.3834	0.3337	0.2549	0.4573
SKCM	0.0035	0.572	0.5185	0.6016	0.0039	0.0681	0.984	0.9391	0.1428	0.2575	0.9936	0.1698
SARC	0.7528	0.7169	0.3616	0.7743	0.4877	0.956	0.3294	0.0407	0.2895	0.1719	0.5406	0.5826
DLBC	0.232	0.7333	0.4759	0.0278	0.271	0.2998	0.5372	0.442	0.5147	0.0459	0.0108	0.6573
THYM	0.1011	0.4579	0.096	0.9448	0.0538	0.4323	0.1613	0.6244	0.3424	0.9481	0.2526	0.0594

Supplementary Table S8. Pathway Signatures

Disease	Pathway	p-value	FDR adjusted p-value	P-value from random signatures
ACC	Cell cycle	1.68E-03	2.02E-02	0.089
ACC	EMT	6.19E-03	3.71E-02	0.117
BLCA	Hormone receptor	3.25E-02	3.91E-01	0.016
BRCA	Breast reactive	4.80E-03	2.88E-02	0.125
BRCA	Core reactive	2.96E-03	2.88E-02	0.096
CORE	Cell cycle	1.37E-02	1.64E-01	0.039
DLBC	Core reactive	2.78E-02	1.67E-01	0.028
DLBC	TSC/mTOR	4.59E-02	1.84E-01	0.031
DLBC	Hormone receptor	1.08E-02	1.30E-01	0
KICH	EMT	1.70E-03	2.04E-02	0.006
KICH	RTK	1.29E-02	7.77E-02	0.045
KIRC	RTK	2.24E-07	2.69E-06	0.037
KIRC	Apoptosis	5.20E-02	6.93E-02	0.48
KIRC	Cell cycle	1.08E-02	2.15E-02	0.4
KIRC	Core reactive	2.06E-04	1.24E-03	0.222
KIRC	DNA damage response	2.49E-02	4.24E-02	0.476
KIRC	EMT	2.82E-02	4.24E-02	0.471
KIRC	PI3K/AKT	3.60E-03	1.08E-02	0.36
KIRC	RAS/MAPK	2.34E-03	9.35E-03	0.356
KIRC	Hormone receptor	1.07E-02	2.15E-02	0.317
KIRP	TSC/mTOR	5.60E-03	6.71E-02	0.039
LGG	DNA damage response	1.72E-07	2.06E-06	0
LGG	Hormone signaling (Breast)	6.13E-05	3.68E-04	0.019
LGG	EMT	1.66E-02	3.98E-02	0.159
LGG	RAS/MAPK	2.26E-02	4.51E-02	0.143
LGG	RTK	4.39E-03	1.32E-02	0.131
LGG	Hormone receptor	5.63E-04	2.25E-03	0.058
LIHC	Hormone signaling (Breast)	2.63E-02	3.16E-01	0.009
LUSC	Breast reactive	1.40E-02	8.39E-02	0.026
LUSC	RTK	7.16E-03	8.39E-02	0.015
MESO	Cell cycle	3.52E-05	4.23E-04	0
PCPG	Apoptosis	4.64E-04	5.57E-03	0
PCPG	Core reactive	3.52E-02	2.11E-01	0
SKCM	Apoptosis	3.46E-03	2.33E-02	0.016
SKCM	DNA damage response	3.88E-03	2.33E-02	0.006
STAD	RTK	1.35E-03	1.63E-02	0.003
TGCT	Apoptosis	8.40E-02	9.10E-01	0.024
THCA	TSC/mTOR	6.62E-03	7.95E-02	0.011
UCEC	Hormone receptor	4.47E-03	5.37E-02	0.015

Supplementary Table S9. The estimated TSC/mTOR network for LUAD patients using PRECISE algorithm. The first two columns represent source and target node names with the types of edges in the third column. The last three columns display proportions of the edges that appears in the PRECISE networks obtained from 100 subsampled dataset (80% of the samples).

**Node A**	**Node B**	**Type**	**A-B**	**A->B**	**B->A**
RPS6KB1	EIF4EBP1	Bi-directed (A-B)	100	0	0
RPS6	EIF4EBP1	Bi-directed (A-B)	100	0	0
RB1	EIF4EBP1	Bi-directed (A-B)	100	0	0
MTOR	RPS6KB1	Bi-directed (A-B)	100	0	0
RPS6	RPS6KB1	Bi-directed (A-B)	100	0	0
RB1	RPS6KB1	Bi-directed (A-B)	48	23	0
RPS6	MTOR	Bi-directed (A-B)	100	0	0
RPS6	RB1	Directed (A->B)	0	88	0
